# Reliability of phase-specific outcome measurements in change-of-direction tests using a motorized resistance device

**DOI:** 10.3389/fspor.2023.1212414

**Published:** 2023-10-23

**Authors:** Frederic Westheim, Øyvind Gløersen, Damian Harper, Håkon Laugsand, Ola Eriksrud

**Affiliations:** ^1^ Biomechanics Laboratory, Department of Physical Performance, Norwegian School of Sport Sciences, Oslo, Norway; ^2^ School of Sport and Health Sciences, Institute of Coaching and Performance, University of Central Lancashire, Preston, United Kingdom

**Keywords:** phase analysis, reliability, motorized resistance technology, deceleration, re-acceleration

## Abstract

This study aims to determine test-retest reliability of phase-specific information during initial acceleration, deceleration, and re-acceleration phases of different change-of-direction (CoD) tests using a motorized resistance device (MRD). A total of 21 participants (16 males and five females, with mean age of 22.3 ± 3.9 years, body mass of 75.2 ± 6.9 kg, height of 177.9 ± 6.8 cm) completed the modified 505 (m505), 10-0-5, and 15-0-5 CoD tests on four different test sessions while exposed to an external load (3 kg) provided by the MRD. Outcome variables included overall and phase-specific kinetic (force, power, and impulse) and kinematic (time, distance, velocity, and acceleration/deceleration) data during the initial acceleration, deceleration, and re-acceleration phases. The deceleration and re-acceleration phases were further divided into two subphases, namely, early and late subphases, using 50% of maximum velocity. Reliability was assessed using an intraclass correlation coefficient (ICC), coefficient of variation (CV), typical error (TE), and minimal detectable change (MDC). Good to excellent ICC values (>0.75) and acceptable (<10%) to good (<5%) CV values were observed for most outcome measurements. Specifically, 80.1% (822 out of 1,026) of all variables showed good or better relative reliability (i.e., ICC ≥ 0.75), while 97.0% (995 out of 1,026) of all variables showed acceptable or better absolute reliability (i.e., CV < 10%). In conclusion, the present study demonstrates that the MRD can obtain reliable phase-specific outcome measurements across different CoD tests, providing coaches and researchers with new opportunities to advance our understanding of CoD ability and inform more advanced CoD training prescriptions.

## 1. Introduction

Change of direction (CoD) is frequently performed in many field and court-based sports (1) and crucial to many decisive on-field performance actions such as preventing and creating goal scoring opportunities (2). Given such importance, the assessment and monitoring of a player's CoD performance is a routine part of many performance teams' testing battery. The CoD performance requires “rapid and systematically coordinated force application during the braking, plant and propulsive phases of the movement while maintaining optimal body positioning” (3). Therefore, horizontal acceleration and deceleration are fundamental locomotor skills underpinning performance in many CoD maneuvers. Consequently, CoD testing methods should quantify the different phases (initial acceleration, deceleration, and re-acceleration) to help advance the understanding of the CoD performance and to inform more specific CoD training prescription for players.

Currently, a myriad of different tests are used to quantify CoD based on different movement patterns (i.e., sprint and side shuffle), angle of turn(s), number of turns, and duration (4). These differences make comparisons between tests difficult as CoD is a task-specific skill based on the angle of turn and entry velocity (4, 5). Furthermore, in most CoD tests, overall time is often used as the primary outcome measurement, which has some inherent problems. Firstly, the overall time does not quantify the initial acceleration, deceleration, and re-acceleration phases. Secondly, longer tests are not possibly representative of CoD but rather anaerobic capacity and linear sprint ability (6). In fact, even in shorter tests, such as the modified 505 (m505) which consists of two 5 m sprints with a 180-degree turn, superior sprint capacity can still mask the CoD ability (4, 7). In an attempt to mitigate this shortcoming, indirect measures such as CoD deficit have been developed to better quantify and isolate the CoD component (8), which in essence is an attempt to quantify deceleration and re-acceleration. However, the CoD deficit does not enable accurate identification of CoD phases, which is vitally important considering that some athletes have been shown to pace their run-up (initial acceleration to deceleration) based on the demand of the CoD (9). This is also in agreement with anecdotal field-based observations of the authors and colleagues.

Based on the shortcomings of the CoD testing mentioned above, it has been advocated that the CoD tests should directly quantify what happens during the tests and provide continuous measurements (4). Specifically, measurements of the velocity of the center of mass (COM) during the CoD testing have also been advocated (4). Such measurements can be obtained in a laboratory setting (i.e., motion capture). However, this is not practical, and in many cases, not feasible for coaches and other practitioners in the applied setting. Field-based technologies such as global navigation satellite systems (GNSS), local positioning systems (LPS) (10, 11) and laser devices (12) have been used to obtain instantaneous velocity data. However, GNSS and LPS have a limited validity and reliability for short CoD tests (10, 11, 13) with the laser-based system demonstrating highly inconsistent phase-specific measurements during a 90-degree CoD (12).

A recent development of motorized resistance technology may provide an opportunity to obtain continuous outcome measurements of how athletes are moving during the CoD test in both laboratory and field-based environments (14). Specifically, a motorized resistance device (MRD) quantifies time, continuous position, and force exerted on the machine while performing a CoD action. Furthermore, load can be prescribed not only in an absolute manner but also in a phase-specific manner since loads in one phase (i.e., initial acceleration to deceleration) can be set to be different than in another phase (i.e., re-acceleration). Continuous velocity measurements of one MRD have recently been validated against a three-dimensional optical motion analysis system (14), but reliability has not been established to date. With both valid and reliable continuous outcome measurements for CoD tests, more detailed insights can be obtained to direct individualized training prescription. For example, more detailed insights into the deceleration phase, as introduced by Harper and co-workers, with data from a MRD can be explored (15). Furthermore, since momentum could have a significant effect on the CoD performance (16), continuous velocity measurements would allow for the exploration of change in momentum capabilities during CoD, as previously advocated by Nimphius and co-authors (4).

Accordingly, the aim of this study was to assess test–retest reliability of overall and phase-specific information (time, velocity, and distance) with specific deceleration and re-acceleration analysis of time, distance, velocity, acceleration/deceleration, force, power, and impulse for different CoD tests.

## 2. Methods

### 2.1. Subjects

A total of 16 male (age, 23.0 ± 3.7 years; body mass, 77.3 ± 6.8 kg; height, 179.9 ± 3.7 cm) and five female participants (age, 20.0 ± 0.0 years; body mass, 68.6 ± 3.7 kg; height, 171.4 ± 9.5 cm) with experience in soccer (*n* = 8), handball (*n* = 8), and floorball (*n* = 5) completed the study. 19 of the 21 participants completed all four test sessions, whereas one male and one female participant completed only two and three test sessions, respectively (due to the COVID-19 pandemic). Inclusion criteria were familiarity with ball sports CoD movements and no musculoskeletal injury or illness at time of testing that would prevent maximum effort for all test sessions. This study was approved by the Local Ethical Committee and the National Data Protection Agency for Research (reference number: 148213) and conducted in accordance with the Declaration of Helsinki. Prior to participation, all participants provided a written informed consent after being given detailed verbal and written explanation of the purpose, procedures, and risks associated with their participation.

### 2.2. Procedures

Anthropometric measurements (height and body mass) were obtained prior to a standardized warm-up, which included dynamic lower extremity mobility exercises, jogging (forward and backward), butt kicks, front kicks, high knee lifts, side shuffle, carioca, unilateral anterior–posterior and lateral jumps, three progressive sprints (80%, 90%–95% of subjective maximal) with the last sprint having assistance, and two m505 tests on each limb both with and without MRD. In total, the warm-up lasted approximately 25 min. The same warm-up was used for all four sessions, and all participants were instructed to standardize their training 2 days prior to testing. There were seven days (median, interquartile range: 7 days) between test sessions. All participants were tested on the same time of the day (morning or afternoon) based on the first test session using the same footwear.

**Figure 1:**
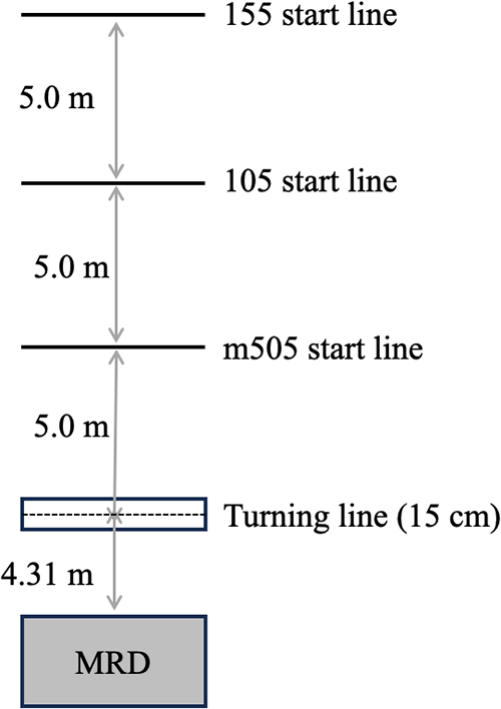
Laboratory set-up with placement of motorized resistance device and position of starting lines and CoD zone for the m505, 105, and 155 tests.

All test sessions took place in an indoor sports hall at the Norwegian School of Sport Sciences with the order and loads used for the different tests being consistent across sessions. Specifically, three different CoD tests were performed with increasing approach distance (5, 10, and 15 m). The m505 test was performed first, then the 10-0-5 (105), and lastly the 15-0-5 (155, the same as the traditional 505 test). During these three tests, the participants performed two successful trials on each limb in an alternate order (e.g., left, right, left, right). Thus, a total of 12 tests (m505L, m505R, 105L, 105R, 155L, and 155R) were performed. The procedures of the m505 test under loaded conditions were previously described in detail (14) but were summarized here for clarity as additional tests were used. A line defining the turning point was 15 cm wide and marked with three cones on each side of a 1.2-m-wide corridor. From the turning point, cones were placed at a distance of 5, 10, and 15 m to define the starting points (Figure 1). All tests were performed under externally loaded conditions provided by an MRD with an assisted start, which meant that the first phase (1a) was sprinting toward, while the second phase (1b) was sprinting away from the MRD past the 5 m mark. The same start position (two-point start) was used at a distance of 5, 10, and 15 m away from the turning point. The fiber cord from the MRD was attached to the participant using a carabiner onto a pulley (Cyclone 52, Purmotion, USA), which in turn was attached to a belt with two carabiners (1080 Vest and 1080 MAP AS, Oslo, Norway, USA). When turning off the left foot, the carabiners were attached over the right hip and for right foot turns vice versa. This was to ensure that the fiber cord from the resistance device was not in conflict with the CoD movement. As the initial acceleration was toward the MRD, a greater demand was placed on both the deceleration and re-acceleration. Left and right foot turns were performed in a randomized order and maintained for all test sessions. A successful trial was defined as a full effort with the final foot contact hitting the 15-cm-wide line between the cones defining the turning point. A minimum of 2-min rest period was given between trials. The trial with the best overall time from each limb during each CoD test was used for further analysis.

### 2.3. Equipment

A portable MRD (1080 Sprint; 1080 Motion, Lidingö, Sweden) was used to provide external resistance and measure time, distance as well as average and maximum velocity (*V*_avg_ and *V*_max_), pulling force (*F*_avg_ and *F*_max_), and power (*P*_avg_ and *P*_max_). The 1080 Sprint has a servo motor (2,000 RPM OMRON G5 Series Motor; OMRON Corp., Kyoto, Japan) that is attached to a carbon fiber spool around which a fiber cord is wrapped. The device was positioned on a table at a height of 75 cm to approximately align with the hip height of the participants. Both assisted and resisted loads were set to 3 kg. The auto-start function of the MRD was used (onset of measurement with a speed of >0.2 m·s^−1^) (17).

### 2.4. Data analysis

All CoD tests were quantified based on time and velocity. Specifically, CoD tests were divided into phase 1a (initial acceleration to deceleration) and phase 1b (re-acceleration), based on when the velocity changed direction (*V*_0_) (14). Consequently, the overall time and phase times (1a and 1b) as well as the maximum and average velocity of phases 1a and 1b were measured.

**Figure 2:**
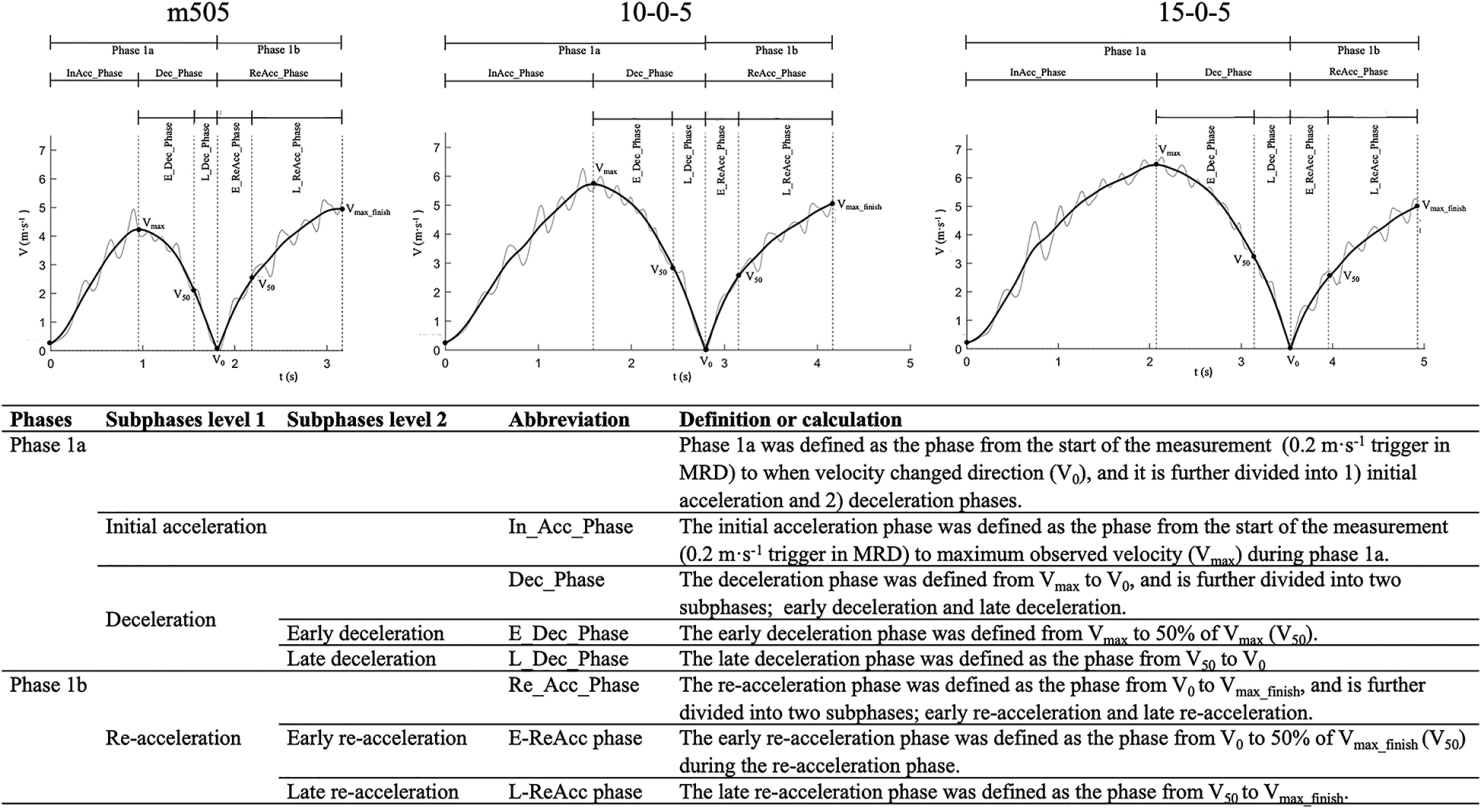
Phase definitions with figures (top row) of the m505, 105, and 155 tests. The table in the bottom rows provides an overview of the phases and subphases with abbreviations used for the outcome measurements and definitions..

In order to provide a more detailed description of phase 1a, the deceleration phase was analyzed based on the methods first described by Harper and co-authors (15) but summarized here for clarity. Similar to the radar device software used in the study by Harper et al. (2020), a fourth-order two-way Butterworth low-pass filter with cut-off frequency of 1.5 Hz was used. Specifically, data on the position, velocity, and force were filtered in this manner, while acceleration was calculated from the filtered velocity with a finite difference using a five-point stencil using MATLAB R2021a (The MathWorks Inc., Natick, MA, USA). From the filtered velocity, the maximum velocity (*V*_max_) during phase 1a was identified. The deceleration phase was then defined from *V*_max_ to when the velocity changed direction *V*_0_. This phase was then further divided into early deceleration phase [*V*_max_ to 50% *V*_max_ (*V*_50_)] and late deceleration phase (*V*_50_ to *V*_0_). During these two phases, different time and distance variables were quantified. Then, based on the change in velocity during the deceleration phase, the pulling force exerted on the participant from the MRD, air resistance (based on the surface area from height measurement), 19-degree centigrade and atmospheric pressure at sea level, force, power, and impulse were calculated for the overall, early, and late deceleration phases. For a description of deceleration phases, see Figure 2.

Contrary to the methods used by Harper and co-authors (15) where only the deceleration phase was used for analysis, we also analyzed the re-acceleration phase using the same methods as described for the deceleration phase. Specifically, based on the same filtered data, we identified *V*_max_finish_—defined as *V*_0_ to the finish line (i.e., end of test and thus measurements). As an athlete will not reach their maximum velocity during a 5-m acceleration, the point of the maximum velocity for the participants during the re-acceleration coincided with the end of the test if the athlete continues to maximally accelerate throughout the re-acceleration phase. Then, the re-acceleration phase was divided into two phases, namely, early phase and late phase. The early phase was defined from *V*_0_ to reaching 50% of *V*_max_finish_ (*V*_50_), while the late re-acceleration phase was defined from *V*_50_ to *V*_max_finish_. The filtered instantaneous velocity–time data were used to calculate force, power, and impulse for both the early and late re-acceleration and deceleration phases. The re-acceleration and deceleration phases are illustrated in Figure 2, while the complete summary of all phase-specific outcome measurements is presented in Table 1.

**Table 1:** Descriptions and definitions of the outcome variables.

Phase	Variable category	Variable	Abbreviation	Definitions or calculations
1a				Phase 1a was defined as the phase from the start of the measurement (0.2 m·s^−1^ trigger in MRD) to when velocity changed direction (V_0_), and it is further divided into (1) initial acceleration and (2) deceleration phases.
1b				The re-acceleration phase was defined as the phase from V_0_ to V_max_ and is further divided into two subphases; early re-acceleration and late re-acceleration.
1a and 1b	Kinematic	Time (s)	Time	Time to complete the test from start to finish.
1a	Kinematic	Time (s)	Time	Phase 1a was defined as the phase from the start of the measurement (0.2 m·s^−1^ trigger in MRD) to when velocity changed direction (V_0_), and it is further divided into (1) initial acceleration and (2) deceleration phases.
		Distance (m)	Dist	Distance from the start of the measurement to the end of phase 1a.
		Average velocity (m·s^−1^)	V_avg_	Average of all instantaneous velocity values of phase 1a.
		Maximum velocity (m·s^−1^)	V_max_	The maximum instantaneous velocity value during phase 1a. This value defines the start and end of the deceleration and initial acceleration phase respectively.
			Dec_Phase	The deceleration phase was defined from V_max_ to V_0_ and is further divided into two subphases: Early deceleration and late deceleration.
Deceleration	Kinematic	Time (s)	Dec_Phase_Time	Time in deceleration phase.
		Distance (m)	Dec_Phase_Dist	Distance covered in the deceleration phase.
		Average deceleration (m·s^−2^)	Dec_Phase_Dec_avg_	Average of all instantaneous deceleration values during the deceleration phase
		Maximum deceleration (m·s^−2^)	Dec_Phase_Dec_max_	Maximum instantaneous deceleration during the deceleration phase
		Time to maximum deceleration (s)	Dec_Phase_Dec_max__Time	Time to maximum deceleration from the start of the deceleration phase.
		Distance to maximum deceleration (m)	Dec_Phase_Dec_max__Dist	Distance to maximum deceleration from the start of phase 1a.
	Kinetic	Average horizontal braking force (N)	Dec_Phase_HBF_avg_	Average of all instantaneous horizontal braking force (HBF) values during the deceleration phase.
		Maximum horizontal braking force (N)	Dec_Phase_HBF_max_	Maximum instantaneous HBF value during the deceleration phase
		Average horizontal braking impulse (N·s)	Dec_Phase_HBI_avg_	Average horizontal braking impulse (HBI) calculated from the instantaneous momentums (F • t = m • Δv) during the deceleration phase.
		Average braking power (W)	Dec_Phase_HBP_avg_	Average of all instantaneous horizontal braking power (HBP) values obtained during the deceleration phase
		Maximum braking power (W)	Dec_Phase_HBP_max_	Maximum instantaneous HBP value during the deceleration phase.
			E_Dec_phase	The early deceleration phase was defined from V_max_ to 50% of V_max_ (V_50_).
Early deceleration	Kinematic	Time (s)	E_Dec_Phase_Time	Time in the early deceleration phase
		Distance (m)	E_Dec_Phase_Dist	Distance covered in the early deceleration phase
		Average velocity (m·s^−1^)	E_Dec_Phase_V_avg_	Average of all instantaneous velocity values during the early deceleration phase
		Average deceleration (m·s^−2^)	E_Dec_Phase_Dec_avg_	Average of all instantaneous deceleration values during the early deceleration phase
	Kinetic	Average horizontal braking force (N)	E_Dec_Phase_HBF_avg_	Average of all instantaneous HBF values during the early deceleration phase
		Average horizontal braking impulse (N·s)	E_Dec_Phase_HBI_avg_	Average HBI calculated from the instantaneous momentums (F • t = m • Δv) during the early deceleration phase.
		Average horizontal braking power (W)	E_Dec_Phase_HBP_avg_	Average of all instantaneous HBP values obtained during early deceleration phase.
			L_Dec_Phase	The late deceleration phase was defined as the phase from V_50_ to V_0_
Late deceleration	Kinematic	Time (s)	L_Dec_Phase_Time	Time in late deceleration phase
		Distance (m)	L_Dec_Phase_Dist	Distance covered in late deceleration phase
		Average velocity (m·s^−1^)	L_Dec_Phase_V_avg_	Average of all instantaneous velocity values during the late deceleration phase
		Average deceleration (m·s^−2^)	L_Dec_Phase_Dec_avg_	Average of all instantaneous deceleration values during the late deceleration phase
	Kinetic	Average horizontal braking force (N)	L_Dec_Phase_HBF_avg_	Average of all instantaneous HBF values during the late deceleration phase
		Horizontal braking impulse (N·s)	L_Dec_Phase_HBI_avg_	Average HBI calculated from the instantaneous momentums (F • t = m • Δv) during the late deceleration phase.
		Average horizontal braking power (W)	L_Dec_Phase_HBP_avg_	Average of all instantaneous HBP values obtained during late deceleration phase.
			ReAcc_Phase	The re-acceleration phase was defined as the phase from V_0_ to V_max_finish_, which is further divided into two subphases; early re-acceleration and late re-acceleration.
Re-acceleration	Kinematic	Time (s)	ReAcc_Phase_Time	Time in re-acceleration phase.
		Distance (m)	ReAcc_Phase_Dist	Distance covered in the re-acceleration phase.
		Average velocity (m·s^−1^)	ReAcc_Phase_Dist_V_avg_	Average of all instantaneous velocity values during the re-acceleration phase.
		Maximum velocity (m·s^−1^)	ReAcc_Phase_V_max_	Maximum instantaneous velocity measurement during the re-acceleration phase.
		Average acceleration (m·s^−2^)	ReAcc_Phase_Acc_avg_	Average of instantaneous acceleration values during the re-acceleration phase.
		Maximum acceleration (m·s^−2^)	ReAcc_Phase_Acc_max_	Maximum instantaneous acceleration measurement during the re-acceleration phase.
	Kinetic	Average horizontal acceleration force (N)	ReAcc_Phase_HAF_avg_	Average of all instantaneous horizontal acceleration force (HAF) values during the re-acceleration phase.
		Maximum horizontal acceleration force (N)	ReAcc_Phase_HAF_max_	Maximum instantaneous HAF during the re-acceleration phase.
		Average horizontal acceleration impulse (N·s)	ReAcc_Phase_HAI_avg_	Average HAI calculated from the instantaneous momentums (F • t = m • Δv) during the re-acceleration phase.
		Average horizontal acceleration power (W)	ReAcc_Phase_HAP_avg_	Average of instantaneous horizontal acceleration power (HAP) values during the re-acceleration phase.
		Maximum horizontal acceleration power (W)	ReAcc_Phase_HAP_max_	Maximum instantaneous HAP value during the re-acceleration phase.
			E_ReAcc_Phase	The early re-acceleration phase was defined as the phase from V_0_ to 50% of V_max_finish_ (V_50_) during the re-acceleration phase.
Early re-acceleration	Kinematic	Time (s)	E_ReAcc_Phase_Time	Time in early re-acceleration phase.
		Distance (m)	E_ReAcc_Phase_Dist	Distance covered in the early re-acceleration phase.
		Average velocity (m·s^−1^)	E_ReAcc_Phase_V_avg_	Average of all instantaneous velocity values during the early re-acceleration phase.
		Average acceleration (m·s^−2^)	E_ReAcc_Phase_Acc_avg_	Average of all instantaneous acceleration values during the early re-acceleration phase.
	Kinetic	Average horizontal acceleration force (N)	E_ReAcc_Phase_HAF_avg_	Average of all instantaneous horizontal acceleration force (HAF) values during the early re-acceleration phase.
		Horizontal acceleration impulse (N·s)	E_ReAcc_Phase_HAI_avg_	Average HAI calculated from the instantaneous momentums (F • t = m • Δv) during the early re-acceleration phase.
		Average horizontal acceleration power (W)	E_ReAcc_Phase_HAP_avg_	Average of instantaneous HAP values during the early re-acceleration phase.
			L_ReAcc_Phase	The late re-acceleration phase was defined as the phase from V_50_ to V_max_finish_.
Late re-acceleration	Kinematic	Time (s)	L_ReAcc_Phase_Time	Time in late re-acceleration phase.
		Total distance of the late re-acceleration phase (m)	L_ReAcc_Phase_Dist	Distance covered in the late re-acceleration phase.
		Average velocity (m·s^−1^)	L_ReAcc_Phase_V_avg_	Average of all instantaneous velocity values during the late re-acceleration phase.
		Average acceleration (m·s^−2^)	L_ReAcc_Phase_Acc_avg_	Average of all instantaneous acceleration values during the late re-acceleration phase.
	Kinetic	Average horizontal acceleration force (N)	L_ReAcc_Phase_HAF_avg_	Average of all instantaneous HAF values during the late re-acceleration phase.
		Late horizontal acceleration impulse (N·s)	L_ReAcc_Phase_HAI_avg_	Average HAI calculated from the instantaneous momentums (F • t = m • Δv) during the late re-acceleration phase.
		Average horizontal acceleration power (W)	L_ReAcc_Phase_HAP_avg_	Average of instantaneous HAP values during the late re-acceleration phase.

### 2.5. Statistical analysis

The statistical analysis was performed using JASP (JASP version 0.16.3.0, Amsterdam, the Netherlands), Statistical Package for Social Sciences (SPSS version 24.0, IBM Corp., Armonk, NY, USA), jamovi (The jamovi project, 2021, version 2.3.6), and RStudio (RStudio Team, 2022). Descriptive data were calculated in Microsoft Excel (version 2209, Microsoft Corp., Redmond, WA, USA) and were presented as mean and standard deviation (SD). Normality of data was assessed using Shapiro–Wilk's test (*p* < 0.05) and qualitatively by visual inspection of Q–Q plots. Furthermore, potential outliers in the data were also explored using a web-based outlier calculator (StatsKingdom, 2017) based on the Tukey's fences method, where *k* was set at 1.5 and indicative of a “regular outlier” (18).

To explore potential differences and learning effect between test sessions in an overall performance and the phase-specific outcome measurements, a repeated measures analysis of variances (RM ANOVAs) was conducted. Alternatively, the Friedman test was performed in cases where the data did not suffice to the assumption of normality. With many variables and therefore many tests performed, the *p*-values from the RM ANOVAs/Friedman tests were corrected with Holm's sequential Bonferroni procedure (19) by using a Microsoft Excel calculator (20) to keep the nominal alpha level at the desired level and avoid type I errors, as recommended by Wagoner (21, 22). The level of statistical significance for the resulting adjusted *p*-values was set at *p* = <0.10.

The assumption of sphericity for the RM ANOVA tests was tested with Mauchly's test of sphericity. If the test indicated that the assumption had been violated (*p*-value of <0.05), then a correction to the original unadjusted *p*-value (“sphericity assumed”) was applied and subsequently used for the analysis. Based on recommendations, if the Greenhouse–Geisser epsilon value (*ε*) from Mauchly's test of sphericity was below 0.75, then a Greenhouse–Geisser correction was applied to the uncorrected *p*-value. Conversely, if the value was ≥0.75, then the Huyndt–Feldt correction was applied (23–25). If a statistically significant difference was found (i.e., *p* = <0.10), then a correction was applied to the original unadjusted *p*-value irrespective of whether or not a violation was indicated by Mauchly's test of sphericity, as has been recommended (23, 24). Furthermore, it is also important to remark that a correction was only applied if the difference was statistically significant (i.e., uncorrected *p*-value = <0.10) in the first place, since a correction in this case will only make a large and non-significant *p*-value even larger and hence increase our change of making a type II error (23, 26).

An effect size was calculated using both omega squared and Kendall's *W* test. Specifically, the omega-squared effect size with a 95% confidence interval (CI) was presented for each RM ANOVA and was calculated using the MOTE Effect Size Calculator app from DOOM Lab (27). Omega-squared values were interpreted in the following manner (24, 28): 0.01 = “small,” 0.06 = “moderate,” and >0.14 = “large.” For the Friedman tests, the measure of effect size was given using the Kendall's W test, where based on guidelines (29) the values of ≤0.1–0.3, ≤0.3–0.5, ≤0.5–0.7, ≤0.7–0.9 and ≤0.9 were interpreted with indications of “very weak agreement”, “weak agreement”, “moderate agreement”, “strong agreement” and “unusually strong agreement”, respectively.

An RM ANOVA or Friedman's test states only whether there is a statistically significant difference somewhere between the testing sessions. Accordingly, if a statistically significant effect was found, then a *post hoc* pairwise comparison was performed to evaluate the statistically significant difference between the test sessions. These were performed on each outcome variable, between each pair of test sessions. For example, test session 1 was compared with test sessions 2, 3, and 4; session 2 was compared with test sessions 3 and 4; and session 3 was compared with session 4. This signified that for one variable, a total of six comparisons were made. Thus, to keep the nominal alpha level at 0.05 and to avoid type I errors, the Holm–Bonferroni correction for multiple comparisons (19) was applied to the *p-*values and used in JASP to adjust for a family of six estimates. Similarly, the 95% CIs for the mean differences given by JASP are also corrected for multiple comparisons using the Bonferroni method. In the case of non-normal data, and if a statistically significant differences based on the Friedman test was found, then the *post hoc* pairwise comparisons were performed with the Wilcoxon matched-pairs test. Accordingly, in this case, the result was presented with the Hodges–Lehmann estimate with a 95% CI as the location parameter, *z*-test statistic value, and *p*-value from the Wilcoxon test. Based on the discussion mentioned above, the *p*-values from the Wilcoxon tests were also corrected with Holm's sequential Bonferroni procedure in JASP, adjusting for a family of six estimates.

As it is recommended to report both unstandardized and standardized effect sizes (30), the standardized Hedges'g average (Hedges'g_av_) was calculated with a 95% CI and provided on all pairs of variables that satisfied the assumption of normality. Magnitude of Hedges'g_av_ values was interpreted according to the guidelines by Hopkins (31, 32): ≤0.19 = “trivial,” 0.20–0.59 = “small,” 0.60–1.19 = “moderate,” 1.20–1.99 = “large,” 2.00–3.99 = “very large,” and ≥4.00 = “extremely large.”

Reliability statistics were obtained using a custom reliability spreadsheet (33). The relative reliability was assessed using the intraclass correlation coefficient (ICC) with a 95% CI. Specifically, the ICC were calculated based on the ICC (3,1) model, referred to as “two-way mixed effects, consistency, single rater/measurement” (34). Of note, this ICC model included only random error in the calculation and does not consider the systematic error. Hopkins noted that the ICC (3,1) model was preferable, as it was the “observed correlation between measurements in two real-life trials” (33). The ICC values were interpreted based on the guidelines presented by Koo and Li (34), where values of <0.50, 0.50–0.75, 0.75–0.90, and >0.90 were interpreted as poor, moderate, good, and excellent reliability, respectively.

The absolute reliability of outcome measurements was assessed using the typical error (TE), both in the raw unit of measurement and relative to the mean based on the log-transformed data (CV—%) with 95% CIs. The CV values of >15%, 10%–15%, 5%–10%, and <5% were used with indications of very poor, poor, acceptable, and good absolute reliability, respectively. Furthermore, to calculate what can be considered as a “real” change in the performance for each respective variable—that is, with a given level of confidence, a change that is outside the ranges of the typical variation (measurement error) of the test and therefore sufficient to be regarded as a “real” change or difference–estimation of the minimal detectable change (MDC) was calculated based on the TE with a 90% CI (MDC_90%_). All reliability statistics and MDC_90%_ were calculated for each session-to-session comparison.

## 3. Results

The descriptive data for the m505, 105, and 155 tests were presented in Tables 2–4, respectively.

**Table 2:** Phase-specific outcome measurements of the m505 test for all sessions.

Phase	Outcome variables	m505 test left				m505 test right			
		Session 1	Session 2	Session 3	Session 4	Session 1	Session 2	Session 3	Session 4
Overall	Time (s)	3.01 ± 0.25	3.04 ± 0.22	3.03 ± 0.21	3.01 ± 0.19	3.04 ± 0.27	3.04 ± 0.21	3.04 ± 0.22	3.01 ± 0.19
Phase 1a	Time (s)	1.70 ± 0.15	1.68 ± 0.14	1.69 ± 0.12	1.68 ± 0.11	1.68 ± 0.16	1.68 ± 0.12	1.69 ± 0.13	1.67 ± 0.11
	Dist (m)	4.73 ± 0.13	4.72 ± 0.11	4.74 ± 0.10	4.72 ± 0.10	4.83 ± 0.22	4.79 ± 0.13	4.80 ± 0.10	4.79 ± 0.14
	V_avg_ (m·s^−1^)	2.84 ± 0.21	2.85 ± 0.21	2.86 ± 0.20	2.87 ± 0.19	2.91 ± 0.23	2.87 ± 0.19	2.87 ± 0.20	2.88 ± 0.16
	V_max_ (m·s^−1^)	4.46 ± 0.25	4.44 ± 0.22	4.46 ± 0.26	4.48 ± 0.23	4.47 ± 0.23	4.45 ± 0.23	4.44 ± 0.24	4.49 ± 0.17
Initial acceleration	Time (s)	0.90 ± 0.13	0.92 ± 0.13	0.91 ± 0.11	0.92 ± 0.13	0.88 ± 0.13	0.92 ± 0.13	0.93 ± 0.11	0.92 ± 0.11
	Dist (m)	2.62 ± 0.27	2.66 ± 0.29	2.63 ± 0.24	2.66 ± 0.23	2.62 ± 0.25	2.69 ± 0.26	2.67 ± 0.21	2.66 ± 0.16
Deceleration	Time (s)	0.80 ± 0.06	0.77 ± 0.08	0.78 ± 0.07	0.75 ± 0.07	0.80 ± 0.08	0.76 ± 0.07	0.76 ± 0.08	0.76 ± 0.07
	Dist (m)	2.16 ± 0.18	2.09 ± 0.22	2.15 ± 0.19	2.11 ± 0.20	2.21 ± 0.19	2.10 ± 0.21	2.13 ± 0.20	2.14 ± 0.18
	Dec_avg_ (m·s^−2^)	5.61 ± 0.65	5.85 ± 0.87	5.79 ± 0.83	5.98 ± 0.70	5.64 ± 0.76	5.88 ± 0.77	5.90 ± 0.89	5.97 ± 0.68
	Dec_max_ (m·s^−2^)	10.4 ± 1.49	11.1 ± 1.50	11.2 ± 1.26	11.6 ± 1.51	10.8 ± 1.76	11.1 ± 1.21	11.6 ± 1.47	11.7 ± 1.30
	Time to Dec_max_ (s)	0.69 ± 0.12	0.66 ± 0.12	0.69 ± 0.10	0.66 ± 0.09	0.70 ± 0.11	0.66 ± 0.10	0.67 ± 0.09	0.67 ± 0.07
	Dist to Dec_max_ (m)	4.75 ± 0.14	4.73 ± 0.11	4.78 ± 0.10	4.75 ± 0.10	4.81 ± 0.20	4.77 ± 0.12	4.80 ± 0.09	4.79 ± 0.14
	HBF_avg_ (N)	458 ± 79.8	479 ± 97.0	473 ± 96.3	490 ± 89.5	460 ± 80.6	479 ± 84.4	482 ± 101	487 ± 82.5
	HBF_max_ (N)	849 ± 157	899 ± 171	913 ± 160	941 ± 178	879 ± 173	901 ± 152	939 ± 174	949 ± 158
	HBI_avg_ (N·s)	361 ± 43.4	359 ± 44.0	360 ± 45.8	363 ± 42.4	361 ± 43.2	360 ± 42.4	359 ± 43.3	363 ± 40.9
	HBP_avg_ (W)	1,032 ± 218	1,075 ± 257	1,067 ± 264	1,108 ± 235	1,040 ± 222	1,076 ± 218	1,085 ± 270	1,102 ± 211
	HBP_max_ (W)	1,745 ± 494	1,844 ± 562	1,825 ± 517	1,928 ± 473	1,809 ± 557	1,850 ± 479	1,906 ± 537	1,930 ± 457
Early deceleration	Time (s)	0.56 ± 0.06	0.55 ± 0.07	0.57 ± 0.07	0.55 ± 0.06	0.58 ± 0.06	0.55 ± 0.06	0.56 ± 0.07	0.56 ± 0.05
	Dist (m)	1.99 ± 0.20	1.94 ± 0.22	2.01 ± 0.20	1.97 ± 0.20	2.06 ± 0.18	1.96 ± 0.20	2.00 ± 0.20	2.01 ± 0.17
	V_avg_ (m·s^−1^)	3.73 ± 0.24	3.74 ± 0.22	3.75 ± 0.23	3.79 ± 0.20	3.76 ± 0.24	3.75 ± 0.23	3.76 ± 0.22	3.80 ± 0.17
	Dec_avg_ (m·s^−2^)	4.00 ± 0.60	4.13 ± 0.73	4.02 ± 0.75	4.15 ± 0.61	3.91 ± 0.54	4.09 ± 0.61	4.04 ± 0.68	4.08 ± 0.50
	HBF_avg_ (N)	328 ± 65.5	339 ± 76.3	330 ± 78.7	341 ± 68.5	321 ± 55.6	334 ± 61.2	331 ± 73.2	334 ± 57.4
	HBI_avg_ (N·s)	180 ± 20.7	180 ± 21.2	180 ± 22.0	182 ± 20.3	181 ± 21.0	180 ± 20.3	180 ± 20.6	182 ± 19.9
	HBP_avg_ (W)	1,110 ± 271	1,141 ± 303	1,118 ± 322	1,157 ± 273	1,086 ± 231	1,128 ± 239	1,118 ± 291	1,134 ± 221
Late deceleration	Time (s)	0.24 ± 0.05	0.22 ± 0.04	0.21 ± 0.03	0.21 ± 0.03	0.22 ± 0.04	0.21 ± 0.02	0.20 ± 0.02	0.20 ± 0.02
	Dist (m)	0.17 ± 0.05	0.15 ± 0.04	0.14 ± 0.03	0.14 ± 0.04	0.15 ± 0.04	0.14 ± 0.02	0.13 ± 0.02	0.13 ± 0.02
	V_avg_ (m·s^−1^)	1.13 ± 0.05	1.12 ± 0.06	1.13 ± 0.06	1.13 ± 0.06	1.13 ± 0.05	1.12 ± 0.05	1.12 ± 0.06	1.13 ± 0.04
	Dec_avg_ (m·s^−2^)	9.75 ± 1.73	10.4 ± 1.76	10.7 ± 1.35	11.0 ± 1.58	10.3 ± 1.87	10.5 ± 1.27	11.1 ± 1.49	11.2 ± 1.32
	HBF_avg_ (N)	795 ± 172	847 ± 184	867 ± 163	898 ± 180	837 ± 182	856 ± 151	901 ± 173	911 ± 157
	HBI_avg_ (N·s)	178 ± 22.4	177 ± 22.4	177 ± 23.5	179 ± 21.7	178 ± 21.9	178 ± 21.7	177 ± 22.3	179 ± 20.6
	HBP_avg_ (W)	886 ± 213	942 ± 231	965 ± 209	1,004 ± 214	937 ± 234	953 ± 194	1,004 ± 237	1,020 ± 199
Re-acceleration	Time (s)	1.32 ± 0.11	1.34 ± 0.11	1.35 ± 0.11	1.33 ± 0.10	1.36 ± 0.13	1.36 ± 0.12	1.36 ± 0.12	1.34 ± 0.11
	Dist (m)	4.79 ± 0.14	4.80 ± 0.14	4.83 ± 0.12	4.82 ± 0.12	4.84 ± 0.19	4.84 ± 0.14	4.86 ± 0.11	4.56 ± 1.14
	V_avg_ (m·s^−1^)	3.43 ± 0.20	3.38 ± 0.19	3.39 ± 0.20	3.41 ± 0.19	3.37 ± 0.20	3.37 ± 0.19	3.39 ± 0.21	3.40 ± 0.18
	V_max_ (m·s^−1^)	5.09 ± 0.33	5.02 ± 0.29	5.03 ± 0.31	5.07 ± 0.33	5.00 ± 0.33	5.03 ± 0.33	5.03 ± 0.32	5.01 ± 0.31
	Acc_avg_ (m·s^−2^)	3.86 ± 0.52	3.76 ± 0.48	3.74 ± 0.48	3.83 ± 0.50	3.69 ± 0.55	3.72 ± 0.50	3.73 ± 0.52	3.54 ± 1.01
	Acc_max_ (m·s^−2^)	9.95 ± 1.41	9.99 ± 1.82	10.4 ± 1.55	10.5 ± 1.74	9.97 ± 1.52	9.93 ± 1.26	10.8 ± 1.39	10.1 ± 2.81
	HAF_avg_ (N)	358 ± 60.2	349 ± 54.4	351 ± 55.8	356 ± 58.1	345 ± 57.3	348 ± 59.2	350 ± 60.0	330 ± 99.9
	HAF_max_ (N)	819 ± 149	822 ± 174	864 ± 166	869 ± 175	823 ± 149	822 ± 131	895 ± 161	826 ± 248
	HAI_avg_ (N·s)	467 ± 52.9	463 ± 50.3	469 ± 52.6	470 ± 55.5	464 ± 50.5	467 ± 53.3	469 ± 53.5	437 ± 121
	HAP_avg_ (W)	975 ± 202	934 ± 179	942 ± 186	965 ± 197	923 ± 194	936 ± 197	942 ± 201	883 ± 290
	HAP_max_ (W)	1,387 ± 324	1,332 ± 277	1,285 ± 262	1,350 ± 261	1,310 ± 274	1,312 ± 259	1,312 ± 275	1,203 ± 390
Early re-acceleration	Time (s)	0.35 ± 0.04	0.36 ± 0.05	0.36 ± 0.05	0.36 ± 0.03	0.37 ± 0.05	0.37 ± 0.04	0.36 ± 0.05	0.35 ± 0.04
	Dist (m)	0.61 ± 0.05	0.62 ± 0.06	0.63 ± 0.05	0.63 ± 0.04	0.63 ± 0.08	0.63 ± 0.06	0.63 ± 0.07	0.61 ± 0.07
	V_avg_ (m·s^−1^)	1.43 ± 0.08	1.42 ± 0.09	1.45 ± 0.10	1.46 ± 0.10	1.43 ± 0.09	1.43 ± 0.07	1.46 ± 0.09	1.44 ± 0.10
	Acc_avg_ (m·s^−2^)	7.33 ± 1.07	7.09 ± 1.25	7.01 ± 1.05	7.16 ± 0.98	6.94 ± 1.23	6.95 ± 1.03	7.11 ± 1.10	6.90 ± 2.00
	HAF_avg_ (N)	626 ± 116	604 ± 119	605 ± 108	614 ± 105	595 ± 113	598 ± 107	614 ± 115	588 ± 180
	HAI_avg_ (N·s)	214 ± 27.1	212 ± 25.7	215 ± 26.3	215 ± 28.3	212 ± 25.9	214 ± 27.1	215 ± 27.4	200 ± 56.4
	HAP_avg_ (W)	815 ± 180	776 ± 176	781 ± 170	797 ± 172	761 ± 176	769 ± 168	792 ± 180	755 ± 251
Late re-acceleration	Time (s)	0.97 ± 0.09	0.98 ± 0.08	0.99 ± 0.08	0.97 ± 0.08	1.00 ± 0.09	0.99 ± 0.09	1.00 ± 0.09	1.00 ± 0.09
	Dist (m)	4.18 ± 0.12	4.18 ± 0.14	4.20 ± 0.11	4.19 ± 0.11	4.21 ± 0.18	4.21 ± 0.13	4.22 ± 0.11	4.22 ± 0.14
	V_avg_ (m·s^−1^)	4.15 ± 0.27	4.09 ± 0.22	4.10 ± 0.24	4.13 ± 0.25	4.09 ± 0.26	4.09 ± 0.25	4.08 ± 0.26	4.08 ± 0.24
	Acc_avg_ (m·s^−2^)	2.63 ± 0.38	2.57 ± 0.34	2.56 ± 0.32	2.61 ± 0.35	2.53 ± 0.39	2.55 ± 0.37	2.54 ± 0.36	2.39 ± 0.69
	HAF_avg_ (N)	263 ± 43.6	257 ± 39.0	259 ± 39.1	263 ± 42.2	255 ± 42.1	257 ± 43.5	244 ± 70.7	217 ± 101
	HAI_avg_ (N·s)	252 ± 25.7	250 ± 24.5	253 ± 26.2	253 ± 27.1	251 ± 24.5	252 ± 26.0	253 ± 26.0	237 ± 64.2
	HAP_avg_ (W)	1,036 ± 220	997 ± 193	1,003 ± 194	1,028 ± 212	987 ± 211	1,001 ± 215	998 ± 211	931 ± 308

Definition and description of all outcome variables are presented in Table 1.

**Table 3:** Phase-specific outcome measurements of the 10-0-5 test for all sessions.

Phase	Outcome variables	10-0-5 test left				10-0-5 test right			
		Session 1	Session 2	Session 3	Session 4	Session 1	Session 2	Session 3	Session 4
Overall	Time (s)	3.96 ± 0.29	3.97 ± 0.27	3.95 ± 0.25	3.89 ± 0.22	3.94 ± 0.27	3.94 ± 0.28	3.95 ± 0.26	3.92 ± 0.22
Phase 1a	Time (s)	2.60 ± 0.19	2.59 ± 0.17	2.58 ± 0.15	2.55 ± 0.13	2.57 ± 0.17	2.57 ± 0.18	2.56 ± 0.17	2.55 ± 0.13
	Dist (m)	9.81 ± 0.11	9.78 ± 0.09	9.80 ± 0.14	9.76 ± 0.12	9.82 ± 0.17	9.83 ± 0.17	9.83 ± 0.15	9.81 ± 0.15
	V_avg_ (m·s^−1^)	3.80 ± 0.26	3.80 ± 0.24	3.81 ± 0.21	3.85 ± 0.19	3.84 ± 0.22	3.85 ± 0.25	3.86 ± 0.23	3.87 ± 0.19
	V_max_ (m·s^−1^)	5.97 ± 0.37	5.93 ± 0.35	5.94 ± 0.33	5.97 ± 0.29	5.92 ± 0.34	5.95 ± 0.38	5.89 ± 0.37	5.98 ± 0.31
Initial acceleration	Time (s)	1.49 ± 0.17	1.50 ± 0.17	1.51 ± 0.15	1.47 ± 0.15	1.47 ± 0.16	1.48 ± 0.17	1.49 ± 0.18	1.49 ± 0.14
	Dist (m)	5.70 ± 0.34	5.78 ± 0.42	5.82 ± 0.45	5.70 ± 0.41	5.67 ± 0.41	5.77 ± 0.43	5.84 ± 0.50	5.78 ± 0.49
Deceleration	Time (s)	1.11 ± 0.10	1.09 ± 0.08	1.07 ± 0.09	1.08 ± 0.09	1.10 ± 0.08	1.08 ± 0.07	1.07 ± 0.08	1.06 ± 0.08
	Dist (m)	4.11 ± 0.37	4.00 ± 0.39	3.97 ± 0.36	4.06 ± 0.36	4.15 ± 0.39	4.05 ± 0.35	3.99 ± 0.43	4.03 ± 0.41
	Dec_avg_ (m·s^−2^)	5.40 ± 0.70	5.47 ± 0.60	5.58 ± 0.65	5.58 ± 0.64	5.38 ± 0.56	5.50 ± 0.58	5.54 ± 0.58	5.66 ± 0.58
	Dec_max_ (m·s^−2^)	10.8 ± 1.43	11.1 ± 1.47	11.4 ± 1.23	11.7 ± 1.50	11.1 ± 1.60	11.2 ± 1.33	11.5 ± 1.33	12.0 ± 1.17
	Time to Dec_max_ (s)	1.02 ± 0.13	0.98 ± 0.12	0.96 ± 0.13	0.97 ± 0.11	0.99 ± 0.10	0.98 ± 0.10	0.96 ± 0.10	0.96 ± 0.11
	Dist to Dec_max_ (m)	9.80 ± 0.11	9.75 ± 0.09	9.77 ± 0.12	9.74 ± 0.11	9.80 ± 0.16	9.81 ± 0.16	9.81 ± 0.13	9.80 ± 0.14
	HBF_avg_ (N)	438 ± 83.6	445 ± 76.6	453 ± 83.8	454 ± 79.4	436 ± 61.0	447 ± 73.6	449 ± 75.2	460 ± 77.3
	HBF_max_ (N)	878 ± 155	902 ± 161	919 ± 146	948 ± 166	903 ± 170	909 ± 152	934 ± 164	974 ± 164
	HBI_avg_ (N·s)	479 ± 62.4	480 ± 61.7	479 ± 61.0	483 ± 59.8	478 ± 57.3	480 ± 63.6	475 ± 60.8	483 ± 59.0
	HBP_avg_ (W)	1,314 ± 307	1,328 ± 283	1,354 ± 294	1,363 ± 285	1,296 ± 231	1,338 ± 283	1,330 ± 269	1,381 ± 278
	HBP_max_ (W)	1,932 ± 461	2,028 ± 461	2,052 ± 500	2,153 ± 514	2,030 ± 525	2,051 ± 477	2,050 ± 512	2,186 ± 537
Early deceleration	Time (s)	0.80 ± 0.08	0.78 ± 0.08	0.78 ± 0.09	0.79 ± 0.08	0.81 ± 0.07	0.79 ± 0.07	0.78 ± 0.07	0.78 ± 0.08
	Dist (m)	3.74 ± 0.35	3.65 ± 0.38	3.65 ± 0.37	3.74 ± 0.36	3.82 ± 0.38	3.72 ± 0.35	3.68 ± 0.42	3.72 ± 0.41
	V_avg_ (m·s^−1^)	4.87 ± 0.32	4.84 ± 0.29	4.87 ± 0.29	4.91 ± 0.26	4.90 ± 0.31	4.91 ± 0.33	4.87 ± 0.34	4.93 ± 0.27
	Dec_avg_ (m·s^−2^)	3.78 ± 0.53	3.82 ± 0.48	3.86 ± 0.54	3.81 ± 0.47	3.69 ± 0.40	3.81 ± 0.49	3.79 ± 0.47	3.86 ± 0.49
	HBF_avg_ (N)	306 ± 60.2	311 ± 56.3	314 ± 62.3	310 ± 54.6	298 ± 40.7	309 ± 57.1	307 ± 54.5	314 ± 57.0
	HBI_avg_ (N·s)	239 ± 30.2	240 ± 30.3	239 ± 29.4	241 ± 28.9	238 ± 28.3	239 ± 31.0	237 ± 29.5	241 ± 28.7
	HBP_avg_ (W)	1,381 ± 332	1,393 ± 312	1,411 ± 325	1,399 ± 296	1,332 ± 233	1,393 ± 321	1,366 ± 290	1,416 ± 308
Late deceleration	Time (s)	0.32 ± 0.04	0.31 ± 0.04	0.29 ± 0.03	0.29 ± 0.04	0.30 ± 0.04	0.30 ± 0.03	0.28 ± 0.03	0.28 ± 0.03
	Dist (m)	0.37 ± 0.07	0.35 ± 0.07	0.33 ± 0.05	0.31 ± 0.06	0.32 ± 0.06	0.33 ± 0.06	0.31 ± 0.05	0.31 ± 0.07
	V_avg_ (m·s^−1^)	1.57 ± 0.10	1.55 ± 0.12	1.54 ± 0.09	1.55 ± 0.09	1.52 ± 0.10	1.55 ± 0.11	1.52 ± 0.11	1.55 ± 0.12
	Dec_avg_ (m·s^−2^)	9.61 ± 1.51	9.83 ± 1.47	10.2 ± 1.29	10.6 ± 1.51	10.1 ± 1.51	10.1 ± 1.30	10.5 ± 1.35	10.8 ± 1.33
	HBF_avg_ (N)	778 ± 156	801 ± 155	828 ± 152	859 ± 164	821 ± 158	816 ± 141	849 ± 157	878 ± 164
	HBI_avg_ (N·s)	238 ± 31.9	238 ± 31.1	238 ± 31.2	239 ± 30.5	238 ± 28.7	238 ± 32.3	236 ± 30.9	240 ± 30.0
	HBP_avg_ (W)	1,164 ± 281	1,189 ± 264	1,230 ± 265	1,284 ± 285	1,217 ± 272	1,218 ± 261	1,251 ± 275	1,311 ± 275
Re-acceleration	Time (s)	1.36 ± 0.11	1.38 ± 0.11	1.37 ± 0.11	1.35 ± 0.11	1.37 ± 0.12	1.38 ± 0.12	1.39 ± 0.12	1.37 ± 0.12
	Dist (m)	4.82 ± 0.11	4.78 ± 0.09	4.81 ± 0.13	4.77 ± 0.12	4.83 ± 0.17	4.82 ± 0.16	4.84 ± 0.15	4.82 ± 0.16
	V_avg_ (m·s^−1^)	3.40 ± 0.23	3.34 ± 0.22	3.37 ± 0.19	3.40 ± 0.21	3.39 ± 0.22	3.36 ± 0.23	3.35 ± 0.21	3.39 ± 0.21
	V_max_ (m·s^−1^)	5.22 ± 0.39	5.14 ± 0.40	5.14 ± 0.40	5.16 ± 0.37	5.22 ± 0.42	5.15 ± 0.43	5.10 ± 0.41	5.15 ± 0.37
	Acc_avg_ (m·s^−2^)	3.86 ± 0.56	3.76 ± 0.54	3.77 ± 0.52	3.86 ± 0.55	3.85 ± 0.62	3.77 ± 0.60	3.70 ± 0.56	3.80 ± 0.55
	Acc_max_ (m·s^−2^)	10.10 ± 1.69	9.88 ± 1.88	10.3 ± 1.71	10.6 ± 1.91	9.94 ± 1.68	10.0 ± 1.68	10.3 ± 1.65	10.7 ± 1.67
	HAF_avg_ (N)	357 ± 60.6	349 ± 61.6	353 ± 60.5	360 ± 61.5	358 ± 66.3	350 ± 65.9	355 ± 60.4	352 ± 63.8
	HAF_max_ (N)	830 ± 156	816 ± 171	853 ± 158	876 ± 180	823 ± 159	825 ± 165	855 ± 173	890 ± 175
	HAI_avg_ (N·s)	480 ± 56.9	475 ± 59.5	477 ± 59.7	479 ± 56.1	482 ± 58.5	475 ± 61.9	474 ± 59.7	478 ± 55.6
	HAP_avg_ (W)	991 ± 216	953 ± 217	964 ± 214	987 ± 217	993 ± 242	959 ± 235	940 ± 215	970 ± 213
	HAP_max_ (W)	1,299 ± 279	1,244 ± 302	1,302 ± 299	1,276 ± 264	1,301 ± 279	1,263 ± 266	1,223 ± 227	1,259 ± 231
Early re-acceleration	Time (s)	0.38 ± 0.05	0.39 ± 0.05	0.37 ± 0.05	0.36 ± 0.04	0.38 ± 0.05	0.38 ± 0.05	0.38 ± 0.05	0.37 ± 0.04
	Dist (m)	0.67 ± 0.07	0.68 ± 0.08	0.66 ± 0.09	0.65 ± 0.04	0.68 ± 0.07	0.67 ± 0.08	0.67 ± 0.09	0.67 ± 0.06
	V_avg_ (m·s^−1^)	1.50 ± 0.12	1.47 ± 0.12	1.48 ± 0.12	1.49 ± 0.12	1.49 ± 0.11	1.48 ± 0.10	1.47 ± 0.11	1.49 ± 0.12
	Acc_avg_ (m·s^−2^)	7.01 ± 1.12	6.77 ± 1.16	6.98 ± 1.09	7.18 ± 1.13	6.88 ± 1.21	6.84 ± 1.14	6.81 ± 1.12	6.98 ± 1.04
	HAF_avg_ (N)	599 ± 104	582 ± 114	602 ± 107	618 ± 116	592 ± 117	588 ± 119	587 ± 113	603 ± 110
	HAI_avg_ (N·s)	221 ± 29.8	219 ± 30.9	220 ± 31.2	220 ± 28.6	222 ± 30.2	219 ± 31.8	218 ± 31.1	220 ± 28.2
	HAP_avg_ (W)	802 ± 181	767 ± 187	794 ± 180	820 ± 197	795 ± 201	777 ± 201	770 ± 185	798 ± 182
Late re-acceleration	Time (s)	0.98 ± 0.09	0.99 ± 0.09	1.00 ± 0.10	0.98 ± 0.08	0.98 ± 0.10	1.00 ± 0.10	1.01 ± 0.10	0.99 ± 0.09
	Dist (m)	4.15 ± 0.10	4.11 ± 0.11	4.15 ± 0.17	4.12 ± 0.12	4.15 ± 0.15	4.15 ± 0.16	4.17 ± 0.14	4.16 ± 0.16
	V_avg_ (m·s^−1^)	4.14 ± 0.29	4.07 ± 0.30	4.09 ± 0.27	4.11 ± 0.24	4.14 ± 0.30	4.09 ± 0.31	4.06 ± 0.28	4.10 ± 0.27
	Acc_avg_ (m·s^−2^)	2.68 ± 0.41	2.62 ± 0.41	2.61 ± 0.43	2.65 ± 0.38	2.69 ± 0.47	2.62 ± 0.45	2.56 ± 0.42	2.62 ± 0.41
	HAF_avg_ (N)	266 ± 45.7	261 ± 46.9	262 ± 47.1	265 ± 43.2	267 ± 50.5	261 ± 49.3	257 ± 46.2	262 ± 43.8
	HAI_avg_ (N·s)	258 ± 27.0	255 ± 28.6	257 ± 28.6	257 ± 27.5	258 ± 28.1	255 ± 30.0	255 ± 28.5	257 ± 27.4
	HAP_avg_ (W)	1,067 ± 240	1,031 ± 246	1,035 ± 244	1,050 ± 227	1,074 ± 274	1,035 ± 262	1,009 ± 237	1,037 ± 230

Definition and description of all outcome variables are presented in Table 1.

**Table 4:** Phase-specific outcome measurements of the 15-0-5 test for all sessions.

Phase	Outcome variables	15-0-5 test left				15-0-5 test right			
		Session 1	Session 2	Session 3	Session 4	Session 1	Session 2	Session 3	Session 4
Overall	Time (s)	4.68 ± 0.32	4.68 ± 0.30	4.70 ± 0.33	4.65 ± 0.23	4.71 ± 0.31	4.71 ± 0.33	4.72 ± 0.33	4.66 ± 0.25
Phase 1a	Time (s)	3.33 ± 0.23	3.33 ± 0.20	3.35 ± 0.23	3.29 ± 0.15	3.33 ± 0.20	3.34 ± 0.22	3.33 ± 0.22	3.29 ± 0.16
	Dist (m)	14.8 ± 0.16	14.7 ± 0.11	14.8 ± 0.11	14.8 ± 0.13	14.8 ± 0.22	14.8 ± 0.14	14.8 ± 0.15	14.8 ± 0.15
	V_avg_ (m·s^−1^)	4.47 ± 0.30	4.45 ± 0.25	4.44 ± 0.27	4.50 ± 0.19	4.48 ± 0.25	4.45 ± 0.27	4.46 ± 0.26	4.52 ± 0.21
	V_max_ (m·s^−1^)	6.79 ± 0.49	6.79 ± 0.46	6.73 ± 0.47	6.81 ± 0.37	6.79 ± 0.49	6.76 ± 0.46	6.73 ± 0.50	6.82 ± 0.36
Initial acceleration	Time (s)	1.97 ± 0.19	1.98 ± 0.17	2.00 ± 0.23	1.98 ± 0.15	1.97 ± 0.20	1.98 ± 0.19	2.00 ± 0.22	2.00 ± 0.16
	Dist (m)	9.03 ± 0.53	8.95 ± 0.44	8.90 ± 0.54	9.05 ± 0.47	8.97 ± 0.60	8.86 ± 0.52	9.04 ± 0.67	9.16 ± 0.68
Deceleration	Time (s)	1.35 ± 0.09	1.35 ± 0.10	1.35 ± 0.09	1.32 ± 0.10	1.36 ± 0.10	1.37 ± 0.10	1.33 ± 0.11	1.29 ± 0.11
	Dist (m)	5.75 ± 0.44	5.79 ± 0.43	5.87 ± 0.49	5.70 ± 0.44	5.86 ± 0.51	5.92 ± 0.53	5.76 ± 0.58	5.66 ± 0.57
	Dec_avg_ (m·s^−2^)	5.05 ± 0.62	5.05 ± 0.62	5.02 ± 0.57	5.21 ± 0.63	5.03 ± 0.59	4.98 ± 0.60	5.08 ± 0.67	5.30 ± 0.65
	Dec_max_ (m·s^−2^)	10.8 ± 1.30	11.2 ± 1.39	11.6 ± 1.39	11.5 ± 1.62	11.2 ± 1.45	11.3 ± 1.38	11.3 ± 1.34	11.8 ± 1.29
	Time to Dec_max_ (s)	1.26 ± 0.15	1.27 ± 0.13	1.26 ± 0.11	1.22 ± 0.11	1.25 ± 0.14	1.28 ± 0.11	1.23 ± 0.14	1.18 ± 0.15
	Dist to Dec_max_ (m)	14.8 ± 0.14	14.7 ± 0.11	14.8 ± 0.11	14.7 ± 0.12	14.8 ± 0.21	14.8 ± 0.13	14.8 ± 0.14	14.8 ± 0.13
	HBF_avg_ (N)	409 ± 72.8	411 ± 78.1	407 ± 73.3	424 ± 77.5	407 ± 68.9	404 ± 70.4	413 ± 82.7	431 ± 83.1
	HBF_max_ (N)	876 ± 133	910 ± 151	944 ± 177	937 ± 186	911 ± 155	920 ± 163	921 ± 161	961 ± 161
	HBI_avg_ (N·s)	548 ± 74.7	548 ± 75.0	542 ± 73.1	550 ± 68.1	547 ± 72.9	546 ± 73.3	543 ± 73.8	549 ± 67.7
	HBP_avg_ (W)	1,397 ± 323	1,403 ± 334	1,375 ± 308	1,448 ± 317	1,389 ± 301	1,373 ± 307	1,398 ± 340	1,473 ± 335
	HBP_max_ (W)	2,126 ± 535	2,085 ± 497	2,106 ± 501	2,173 ± 446	2,117 ± 563	2,050 ± 448	2,157 ± 532	2,264 ± 513
Early deceleration	Time (s)	0.97 ± 0.09	0.98 ± 0.09	1.00 ± 0.09	0.96 ± 0.09	0.99 ± 0.09	1.01 ± 0.10	0.98 ± 0.10	0.96 ± 0.10
	Dist (m)	5.19 ± 0.43	5.24 ± 0.40	5.37 ± 0.48	5.20 ± 0.45	5.35 ± 0.48	5.41 ± 0.54	5.27 ± 0.55	5.20 ± 0.54
	V_avg_ (m·s^−1^)	5.54 ± 0.38	5.53 ± 0.35	5.54 ± 0.38	5.57 ± 0.30	5.54 ± 0.39	5.52 ± 0.38	5.51 ± 0.38	5.59 ± 0.32
	Dec_avg_ (m·s^−2^)	3.54 ± 0.50	3.50 ± 0.49	3.39 ± 0.43	3.58 ± 0.48	3.44 ± 0.43	3.39 ± 0.48	3.46 ± 0.53	3.59 ± 0.49
	HBF_avg_ (N)	286 ± 53.2	284 ± 56.5	274 ± 47.0	289 ± 52.2	277 ± 47.7	274 ± 51.8	281 ± 60.1	291 ± 58.2
	HBI_avg_ (N·s)	272 ± 35.9	273 ± 36.5	270 ± 35.2	274 ± 33.3	272 ± 35.3	272 ± 35.8	271 ± 35.7	273 ± 32.9
	HBP_avg_ (W)	1,469 ± 352	1,456 ± 366	1,392 ± 305	1,485 ± 325	1,422 ± 311	1,402 ± 334	1,430 ± 371	1,495 ± 351
Late deceleration	Time (s)	0.39 ± 0.05	0.37 ± 0.04	0.35 ± 0.05	0.35 ± 0.05	0.36 ± 0.05	0.36 ± 0.03	0.35 ± 0.05	0.34 ± 0.04
	Dist (m)	0.56 ± 0.12	0.55 ± 0.11	0.50 ± 0.10	0.50 ± 0.09	0.51 ± 0.11	0.51 ± 0.07	0.49 ± 0.13	0.46 ± 0.09
	V_avg_ (m·s^−1^)	1.83 ± 0.15	1.86 ± 0.16	1.83 ± 0.14	1.84 ± 0.13	1.82 ± 0.15	1.83 ± 0.14	1.80 ± 0.17	1.81 ± 0.14
	Dec_avg_ (m·s^−2^)	8.94 ± 1.34	9.22 ± 1.22	9.78 ± 1.44	9.80 ± 1.49	9.51 ± 1.41	9.53 ± 1.25	9.77 ± 1.46	10.3 ± 1.43
	HBF_avg_ (N)	728 ± 141	752 ± 147	799 ± 179	805 ± 173	772 ± 143	774 ± 137	794 ± 166	836 ± 167
	HBI_avg_ (N·s)	274 ± 38.5	273 ± 38.3	270 ± 37.6	274 ± 34.5	273 ± 37.5	272 ± 37.3	270 ± 37.9	274 ± 34.5
	HBP_avg_ (W)	1,242 ± 300	1,283 ± 297	1,350 ± 351	1,371 ± 335	1,315 ± 306	1,314 ± 291	1,340 ± 330	1,430 ± 338
Re-acceleration	Time (s)	1.36 ± 0.10	1.36 ± 0.11	1.37 ± 0.12	1.36 ± 0.10	1.39 ± 0.12	1.37 ± 0.11	1.38 ± 0.13	1.37 ± 0.12
	Dist (m)	4.79 ± 0.17	4.74 ± 0.11	4.77 ± 0.12	4.76 ± 0.13	4.84 ± 0.22	4.78 ± 0.11	4.81 ± 0.15	4.82 ± 0.15
	V_avg_ (m·s^−1^)	3.38 ± 0.20	3.35 ± 0.22	3.34 ± 0.22	3.35 ± 0.19	3.35 ± 0.21	3.34 ± 0.23	3.34 ± 0.23	3.38 ± 0.19
	V_max_ (m·s^−1^)	5.16 ± 0.38	5.10 ± 0.39	5.03 ± 0.43	5.12 ± 0.36	5.13 ± 0.44	5.09 ± 0.40	5.07 ± 0.42	5.15 ± 0.36
	Acc_avg_ (m·s^−2^)	3.81 ± 0.52	3.77 ± 0.56	3.71 ± 0.59	3.79 ± 0.53	3.74 ± 0.59	3.73 ± 0.56	3.70 ± 0.61	3.79 ± 0.54
	Acc_max_ (m·s^−2^)	10.0 ± 1.42	10.4 ± 1.54	10.7 ± 1.52	10.5 ± 1.68	10.1 ± 1.41	10.4 ± 1.62	10.3 ± 1.52	10.5 ± 1.69
	HAF_avg_ (N)	355 ± 55.3	350 ± 62.3	346 ± 64.9	352 ± 58.2	349 ± 64.2	347 ± 63.2	346 ± 64.9	354 ± 58.8
	HAF_max_ (N)	828 ± 125	853 ± 146	887 ± 170	866 ± 170	832 ± 144	852 ± 166	852 ± 152	873 ± 168
	HAI_avg_ (N·s)	479 ± 55.5	470 ± 60.4	468 ± 59.4	474 ± 54.4	477 ± 61.6	471 ± 59.0	471 ± 58.4	478 ± 54.6
	HAP_avg_ (W)	973 ± 197	950 ± 219	931 ± 226	954 ± 201	953 ± 227	941 ± 219	935 ± 226	966 ± 204
	HAP_max_ (W)	1,269 ± 251	1,212 ± 268	1,222 ± 275	1,233 ± 264	1,241 ± 248	1,217 ± 231	1,212 ± 259	1,235 ± 230
Early re-acceleration	Time (s)	0.37 ± 0.04	0.37 ± 0.05	0.36 ± 0.04	0.38 ± 0.04	0.38 ± 0.05	0.37 ± 0.05	0.38 ± 0.04	0.37 ± 0.04
	Dist (m)	0.65 ± 0.08	0.64 ± 0.08	0.63 ± 0.07	0.67 ± 0.07	0.67 ± 0.09	0.66 ± 0.08	0.66 ± 0.06	0.66 ± 0.07
	V_avg_ (m·s^−1^)	1.47 ± 0.12	1.46 ± 0.12	1.46 ± 0.14	1.49 ± 0.12	1.47 ± 0.11	1.47 ± 0.11	1.47 ± 0.11	1.48 ± 0.12
	Acc_avg_ (m·s^−2^)	7.10 ± 0.95	7.09 ± 1.20	7.14 ± 1.05	6.86 ± 0.95	6.85 ± 1.05	6.93 ± 1.24	6.82 ± 1.15	7.04 ± 1.08
	HAF_avg_ (N)	609 ± 85.7	604 ± 109	614 ± 113	589 ± 94.0	589 ± 102	596 ± 127	587 ± 109	607 ± 108
	HAI_avg_ (N·s)	220 ± 29.0	216 ± 31.3	214 ± 31.0	219 ± 28.5	219 ± 32.3	217 ± 30.0	217 ± 30.3	220 ± 28.0
	HAP_avg_ (W)	804 ± 150	791 ± 189	795 ± 193	773 ± 163	775 ± 176	781 ± 205	766 ± 188	802 ± 180
Late re-acceleration	Time (s)	0.99 ± 0.09	0.99 ± 0.09	1.01 ± 0.11	0.98 ± 0.09	1.01 ± 0.10	1.00 ± 0.09	1.01 ± 0.10	1.00 ± 0.09
	Dist (m)	4.14 ± 0.15	4.09 ± 0.13	4.14 ± 0.12	4.09 ± 0.16	4.17 ± 0.20	4.12 ± 0.08	4.15 ± 0.16	4.16 ± 0.16
	V_avg_ (m·s^−1^)	4.09 ± 0.27	4.04 ± 0.27	4.01 ± 0.29	4.06 ± 0.24	4.07 ± 0.30	4.04 ± 0.29	4.04 ± 0.29	4.08 ± 0.23
	Acc_avg_ (m·s^−2^)	2.62 ± 0.40	2.59 ± 0.41	2.51 ± 0.44	2.62 ± 0.40	2.58 ± 0.45	2.57 ± 0.40	2.55 ± 0.44	2.60 ± 0.38
	HAF_avg_ (N)	263 ± 43.4	259 ± 46.9	253 ± 47.2	261 ± 44.0	259 ± 49.1	257 ± 44.7	256 ± 48.3	261 ± 42.1
	HAI_avg_ (N·s)	258 ± 26.5	253 ± 29.0	252 ± 28.4	254 ± 25.9	256 ± 29.2	253 ± 28.9	254 ± 28.0	257 ± 26.6
	HAP_avg_ (W)	1,040 ± 227	1,013 ± 241	982 ± 244	1,027 ± 224	1,025 ± 256	1,004 ± 233	1,000 ± 248	1,028 ± 217

Definition and description of all outcome variables are presented in Table 1.

Overall, 80.1% (822 out of 1,026) of all variables showed good or better relative reliability. Specifically, 50.7% (520 out of 1,026) showed excellent, 29.4% (302 out of 1,026) showed good, 14.2% (146 out of 1,026) showed moderate, and 5.7% (58 out of 1,026) of all variables showed poor relative reliability. For the absolute reliability, 97.0% (995 out of 1,026) of all variables showed acceptable or better absolute reliability. Specifically, 58.9% (604 out of 1,026) showed good, 38.1% (391 out of 1,026) showed acceptable, 2.7% (28 out of 1,026) showed poor, and 0.3% (3 out of 1,026) of the variables showed very poor absolute reliability.

### 3.1. M505 test

For the m505L test (Table 5), when combining the three comparisons, 87.7% (150 out of 171) of the variables displayed good or better relative reliability, with ICC values ranging from 0.18 to 0.99. Specifically, 50.3% (86 out of 171) of all variables displayed excellent, 37.4% (64 out of 171) displayed good, 8.8% (15 out of 171) displayed moderate, and 3.5% (6 out of 171) displayed poor relative reliability*.* With regard to CV values, 98.3% (168 out of 171) of all the variables had acceptable or better absolute reliability with a range from 0.8 to 16.0%. Specifically, 57.9% (99 out of 171) of all variables displayed good, 40.4% (69 out of 171) displayed acceptable*,* 1.2% (2 out of 171) displayed poor, and 0.6% (1 out of 171) displayed very poor absolute reliability. Furthermore, the CV values for the comparison of sessions 1–2 ranged from 0.8 to 16.0 (32/57 good), while the CV values for the comparison of sessions 3–4 ranged from 0.8 to 10.1 (32/57 good).

**Table 5:** Test–retest reliability of overall and phase-specific outcome measurement for the m505 test with left foot ultimate step.

Phase	Outcome variables	Test session 1–2 (*n*^1^ = 15; *n*^2^ = 21)				Test session 2–3 (*n^1^* = 16; *n*^2^ = 20)				Test session 3–4 (*n*^1^ = 16; *n*^2^ = 19)			
		ICC (95% CI)	CV (95% CI)	TE (95% CI)	MDC_90%_	ICC (95% CI)	CV (95% CI)	TE (95% CI)	MDC_90%_	ICC (95% CI)	CV (95% CI)	TE (95% CI)	MDC_90%_
Overall	Time (s)	0.94 (0.85; 0.98)	2.0 (1.5; 3.2)	0.06 (0.04; 0.09)	0.14	0.89 (0.71; 0.95)	2.6 (1.9; 4.1)	0.08 (0.06; 0.12)	0.18	0.96 (0.90; 0.99)	1.4 (1.0; 2.1)	0.04 (0.03; 0.06)	0.09
Phase 1a	Time (s)	0.88 (0.73; 0.95)	3.1 (2.3; 4.4)	0.05 (0.04; 0.07)	0.12	0.84 (0.64; 0.93)	3.4 (2.5; 4.9)	0.06 (0.04; 0.08)	0.13	0.89 (0.74; 0.95)	2.4 (1.8; 3.6)	0.04 (0.03; 0.06)	0.10
	Dist (m)	0.78 (0.53; 0.90)	1.2 (1.0; 1.8)	0.06 (0.05; 0.09)	0.14	0.48 (0.05; 0.75)	1.7 (1.3; 2.5)	0.08 (0.06; 0.11)	0.18	0.88 (0.72; 0.95)	0.8 (0.6; 1.2)	0.04 (0.03; 0.05)	0.09
	V_avg_ (m·s^−1^)	0.84 (0.64; 0.93)	3.2 (2.4; 4.6)	0.09 (0.07; 0.13)	0.21	0.86 (0.69; 0.94)	2.9 (2.2; 4.2)	0.08 (0.06; 0.12)	0.19	0.91 (0.78; 0.96)	2.3 (1.7; 3.4)	0.06 (0.05; 0.09)	0.15
	V_max_ (m·s^−1^)	0.82 (0.60; 0.92)	2.4 (1.8; 3.5)	0.10 (0.08; 0.15)	0.24	0.84 (0.65; 0.93)	2.3 (1.7; 3.3)	0.10 (0.08; 0.14)	0.23	0.85 (0.66; 0.94)	2.2 (1.7; 3.3)	0.10 (0.07; 0.15)	0.23
Initial acceleration	Time (s)	0.83 (0.64; 0.93)	6.4 (4.9; 9.3)	0.06 (0.04; 0.08)	0.13	0.80 (0.56; 0.91)	6.5 (4.9; 9.7)	0.06 (0.04; 0.08)	0.13	0.88 (0.72; 0.95)	4.8 (3.6; 7.2)	0.04 (0.03; 0.07)	0.10
	Dist (m)	0.86 (0.68; 0.94)	4.4 (3.4; 6.5)	0.11 (0.08; 0.16)	0.26	0.80 (0.56; 0.91)	5.1 (3.8; 7.5)	0.12 (0.09; 0.18)	0.29	0.71 (0.39; 0.88)	4.9 (3.7; 7.4)	0.13 (0.10; 0.19)	0.30
Deceleration	Time (s)	0.88 (0.73; 0.95)	3.6 (2.7; 5.2)	0.03 (0.02; 0.04)	0.06	0.82 (0.60; 0.92)	4.5 (3.4; 6.6)	0.03 (0.03; 0.05)	0.08	0.81 (0.57; 0.92)	4.2 (3.1; 6.2)	0.03 (0.02; 0.05)	0.07
	Dist (m)	0.74 (0.46; 0.89)	5.2 (3.9; 7.6)	0.11 (0.08; 0.15)	0.25	0.81 (0.59; 0.92)	4.6 (3.5; 6.7)	0.09 (0.07; 0.14)	0.22	0.70 (0.37; 0.87)	5.8 (4.3; 8.7)	0.11 (0.08; 0.16)	0.26
	Dec_avg_ (m·s^−2^)	0.87 (0.70; 0.94)	5.0 (3.8; 7.4)	0.30 (0.23; 0.43)	0.69	0.90 (0.76; 0.96)	5.1 (3.8; 7.5)	0.29 (0.22; 0.42)	0.67	0.86 (0.67; 0.94)	5.4 (4.1; 8.1)	0.30 (0.23; 0.45)	0.70
	Dec_max_ (m·s^−2^)	0.80 (0.58; 0.92)	6.3 (4.8; 9.2)	0.69 (0.53; 0.99)	1.60	0.78 (0.53; 0.91)	6.2 (4.7; 9.2)	0.67 (0.51; 0.98)	1.56	0.83 (0.61; 0.93)	5.2 (3.9; 7.8)	0.60 (0.45; 0.88)	1.39
	Time to Dec_max_ (s)	0.90 (0.78; 0.96)	5.9 (4.5; 8.7)	0.04 (0.03; 0.06)	0.09	0.86 (0.69; 0.94)	6.3 (4.8; 9.3)	0.04 (0.03; 0.06)	0.10	0.75 (0.45; 0.89)	7.8 (5.9; 11.8)	0.05 (0.04; 0.07)	0.12
	Dist to Dec_max_ (m)	0.76 (0.50; 0.90)	1.3 (1.0; 1.9)	0.06 (0.05; 0.09)	0.15	0.43 (0.00; 0.73)	1.7 (1.3; 2.5)	0.08 (0.06; 0.12)	0.19	0.73 (0.42; 0.88)	1.2 (0.9; 1.7)	0.06 (0.04; 0.08)	0.13
	HBF_avg_ (N)	0.94 (0.85; 0.97)	4.9 (3.7; 7.1)	23.2 (17.8; 33.5)	54.0	0.95 (0.87; 0.98)	5.0 (3.8; 7.3)	23.2 (17.7; 33.9)	54.1	0.94 (0.85; 0.98)	5.2 (3.9; 7.7)	24.6 (18.6; 36.3)	57.1
	HBF_max_ (N)	0.89 (0.75; 0.95)	6.1 (4.6; 8.9)	57.0 (43.7; 82.4)	133	0.90 (0.76; 0.96)	6.1 (4.6; 9.1)	55.4 (42.2; 81.0)	129	0.92 (0.81; 0.97)	5.1 (3.8; 7.6)	49.4 (37.3; 73.0)	115
	HBI_avg_ (N·s)	0.97 (0.93; 0.99)	2.3 (1.8; 3.4)	8.00 (6.10; 11.5)	18.5	0.97 (0.92; 0.99)	2.4 (1.9; 3.6)	8.50 (6.50; 12.4)	19.8	0.97 (0.92; 0.99)	2.2 (1.7; 3.3)	8.50 (6.40; 12.6)	19.8
	HBP_avg_ (W)	0.93 (0.84; 0.97)	6.5 (4.9; 9.5)	65.7 (50.3; 94.9)	153	0.95 (0.87; 0.98)	6.2 (4.7; 9.2)	63.8 (48.5; 93.1)	148	0.93 (0.82; 0.97)	6.7 (5.0; 10.1)	71.8 (54.2; 106)	167
	HBP_max_ (W)	0.95 (0.88; 0.98)	7.7 (5.8; 11.3)	123 (94.4; 178)	287	0.92 (0.81; 0.97)	9.7 (7.3; 14.4)	163 (124; 237)	378	0.92 (0.81; 0.97)	8.0 (6.0; 12.1)	146 (110.6; 217)	341
Early deceleration	Time (s)	0.79 (0.55; 0.91)	5.7 (4.3; 8.4)	0.03 (0.02; 0.05)	0.07	0.90 (0.77; 0.96)	4.3 (3.3; 6.4)	0.02 (0.02; 0.03)	0.05	0.78 (0.51; 0.91)	6.1 (4.6; 9.1)	0.03 (0.02; 0.05)	0.08
	Dist (m)	0.73 (0.45; 0.88)	5.9 (4.5; 8.7)	0.11 (0.09; 0.16)	0.26	0.85 (0.67; 0.94)	4.5 (3.4; 6.6)	0.09 (0.07; 0.13)	0.20	0.70 (0.38; 0.87)	6.4 (4.8; 9.5)	0.11 (0.09; 0.17)	0.27
	V_avg_ (m·s^−1^)	0.91 (0.79; 0.96)	2.1 (1.6; 2.9)	0.07 (0.06; 0.11)	0.17	0.89 (0.74; 0.95)	2.2 (1.6; 3.2)	0.08 (0.06; 0.12)	0.18	0.85 (0.64; 0.94)	2.4 (1.8; 3.5)	0.09 (0.07; 0.13)	0.20
	Dec_avg_ (m·s^−2^)	0.81 (0.59; 0.92)	7.7 (5.8; 11.3)	0.30 (0.23; 0.44)	0.70	0.93 (0.84; 0.97)	5.2 (3.9; 7.7)	0.20 (0.16; 0.30)	0.47	0.84 (0.63; 0.93)	7.3 (5.5; 11.0)	0.29 (0.22; 0.43)	0.67
	HBF_avg_ (N)	0.91 (0.78; 0.96)	7.2 (5.5; 10.6)	23.0 (17.6; 33.2)	53.4	0.96 (0.91; 0.99)	4.8 (3.6; 7.1)	15.6 (11.9; 22.8)	36.4	0.91 (0.78; 0.96)	6.9 (5.2; 10.3)	23.6 (17.8; 35.0)	55.0
	HBI_avg_ (N·s)	0.97 (0.94; 0.99)	2.1 (1.6; 3.0)	3.56 (2.73; 5.15)	8.29	0.97 (0.92; 0.99)	2.4 (1.8; 3.5)	4.18 (3.18; 6.10)	9.72	0.97 (0.92; 0.99)	2.1 (1.6; 3.1)	4.07 (3.07; 6.02)	9.46
	HBP_avg_ (W)	0.90 (0.77; 0.96)	9.1 (6.9; 13.4)	95.8 (73.3; 138)	223	0.96 (0.90; 0.98)	6.4 (4.8; 9.5)	68.4 (52.0; 99.9)	159	0.91 (0.77; 0.96)	8.5 (6.3; 12.8)	96.7 (73.1; 143)	225
Late deceleration	Time (s)	0.86 (0.69; 0.94)	8.2 (6.2; 12.1)	0.02 (0.01; 0.03)	0.04	0.78 (0.53; 0.91)	7.5 (5.6; 11.1)	0.02 (0.01; 0.02)	0.04	0.87 (0.70; 0.95)	5.6 (4.2; 8.3)	0.01 (0.01; 0.02)	0.03
	Dist (m)	0.76 (0.50; 0.90)	16.0 (12.1; 24.0)	0.02 (0.02; 0.03)	0.05	0.67 (0.33; 0.85)	14.8 (11.1; 22.4)	0.02 (0.02; 0.03)	0.05	0.86 (0.68; 0.94)	10.1 (7.6; 15.3)	0.01 (0.01; 0.02)	0.03
	V_avg_ (m·s^−1^)	0.74 (0.47; 0.89)	2.6 (2.0; 3.8)	0.03 (0.02; 0.04)	0.07	0.76 (0.50; 0.90)	2.7 (2.0; 4.0)	0.03 (0.02; 0.04)	0.07	0.88 (0.72; 0.95)	1.9 (1.4; 2.8)	0.02 (0.02; 0.03)	0.05
	Dec_avg_ (m·s^−2^)	0.86 (0.68; 0.94)	6.9 (5.2; 10.1)	0.69 (0.53; 1.00)	1.60	0.81 (0.58; 0.92)	7.4 (5.6; 11.0)	0.71 (0.54; 1.04)	1.66	0.84 (0.64; 0.94)	5.6 (4.2; 8.4)	0.61 (0.46; 0.90)	1.41
	HBF_avg_ (N)	0.91 (0.79; 0.96)	6.6 (5.0; 9.6)	56.0 (42.8; 80.8)	130	0.89 (0.75; 0.95)	7.4 (5.6; 11.1)	60.3 (45.9; 88.1)	140	0.93 (0.82; 0.97)	5.3 (4.0; 7.9)	48.6 (36.7; 71.9)	113
	HBI_avg_ (N·s)	0.96 (0.91; 0.98)	2.7 (2.0; 3.9)	4.54 (3.48; 6.56)	10.6	0.97 (0.92; 0.99)	2.6 (2.0; 3.8)	4.44 (3.38; 6.48)	10.3	0.96 (0.91; 0.99)	2.5 (1.9; 3.8)	4.57 (3.45; 6.76)	10.6
	HBP_avg_ (W)	0.95 (0.88; 0.98	5.9 (4.4; 8.6)	52.3 (40.0; 75.5)	122	0.92 (0.81; 0.97)	7.7 (5.8; 11.5)	66.5 (50.6; 97.2)	155	0.92 (0.82; 0.97)	6.1 (4.6; 9.2)	61.3 (46.3; 90.6)	143
Re-acceleration	Time (s)	0.97 (0.91; 0.99)	1.6 (1.2; 2.6)	0.02 (0.02; 0.03)	0.05	0.95 (0.86; 0.98)	2.1 (1.5; 3.3)	0.03 (0.02; 0.04)	0.06	0.93 (0.81; 0.97)	2.2 (1.6; 3.4)	0.03 (0.02; 0.05)	0.07
	Dist (m)	0.77 (0.45; 0.91)	1.5 (1.1; 2.3)	0.07 (0.05; 0.11)	0.16	0.47 (-0.04; 0.76)	2.0 (1.5; 3.1)	0.09 (0.07; 0.15)	0.22	0.76 (0.43; 0.90)	1.2 (0.9; 1.9)	0.06 (0.04; 0.09)	0.14
	V_avg_ (m·s^−1^)	0.96 (0.88; 0.98)	1.2 (0.9; 2.0)	0.04 (0.03; 0.07)	0.10	0.97 (0.93; 0.99)	1.0 (0.8; 1.6)	0.03 (0.03; 0.05)	0.08	0.96 (0.90; 0.99)	1.2 (0.9; 1.9)	0.04 (0.03; 0.06)	0.10
	V_max_ (m·s^−1^)	0.95 (0.87; 0.98)	1.4 (1.0; 2.1)	0.07 (0.05; 0.11)	0.16	0.93 (0.82; 0.97)	1.6 (1.2; 2.5)	0.08 (0.06; 0.13)	0.19	0.96 (0.90; 0.99)	1.3 (1.0; 2.0)	0.06 (0.05; 0.10)	0.15
	Acc_avg_ (m·s^−2^)	0.98 (0.94; 0.99)	1.9 (1.4; 3.0)	0.08 (0.06; 0.12)	0.18	0.97 (0.91; 0.99)	2.4 (1.8; 3.8)	0.09 (0.07; 0.14)	0.22	0.95 (0.87; 0.98)	3.2 (2.3; 4.9)	0.11 (0.08; 0.17)	0.26
	Acc_max_ (m·s^−2^)	0.75 (0.41; 0.90)	8.7 (6.3; 14.1)	0.85 (0.62; 1.34)	1.97	0.93 (0.82; 0.97)	4.8 (3.5. 7.5)	0.47 (0.34; 0.72)	1.08	0.83 (0.58; 0.93)	6.8 (5.0; 10.8)	0.71 (0.52; 1.10)	1.65
	HAF_avg_ (N)	0.99 (0.97; 1.00)	1.7 (1.2; 2.7)	6.55 (4.79; 10.3)	15.2	0.98 (0.95; 0.99)	2.2 (1.6; 3.5)	7.94 (5.86; 12.3)	18.5	0.97 (0.93; 0.99	2.7 (2.0; 4.2)	9.70 (7.17; 15.0)	22.6
	HAF_max_ (N)	0.83 (0.56; 0.93)	8.2 (5.9; 13.2)	71.0 (52.0; 112)	165	0.95 (0.88; 0.98)	4.6 (3.4; 7.2)	38.8 (28.7; 60.1)	90.4	0.91 (0.76; 0.96)	6.2 (4.5; 9.7)	55.4 (40.9; 85.7)	129
	HAI_avg_ (N·s)	0.99 (0.97; 1.00)	1.2 (0.9; 2.0)	5.96 (4.37; 9.41)	13.9	0.98 (0.95; 0.99)	1.6 (1.2. 2.5)	7.39 (5.46; 11.4)	17.2	0.99 (0.97; 1.00)	1.2 (0.9; 1.9)	5.59 (4.13; 8.66)	13.0
	HAP_avg_ (W)	0.98 (0.94; 0.99)	2.7 (2.0; 4.3)	29.0 (21.3; 45.8)	67.5	0.97 (0.93; 0.99)	3.3 (2.4; 5.1)	31.5 (23.3; 48.8)	73.3	0.97 (0.92; 0.99)	3.6 (2.7; 5.6)	34.2 (25.3; 52.9)	79.6
	HAP_max_ (W)	0.89 (0.71; 0.96)	7.4 (5.3; 11.9)	105 (77.2; 166)	245	0.92 (0.80; 0.97)	5.9 (4.3; 9.3)	79.5 (58.7; 123)	185	0.97 (0.92; 0.99)	3.3 (2.4; 5.2)	48.3 (35.7; 74.8)	112
Early re-acceleration	Time (s)	0.63 (0.18; 0.84)	9.1 (6.6; 14.7)	0.03 (0.02; 0.05)	0.07	0.72 (0.37; 0.88)	8.1 (5.9; 12.7)	0.03 (0.02; 0.04)	0.06	0.66 (0.26; 0.86)	6.7 (4.9; 10.5)	0.02 (0.02; 0.04)	0.06
	Dist (m)	0.42 (-0.13; 0.74)	8.1 (5.9; 13.1)	0.05 (0.03; 0.07)	0.11	0.18 (-0.40; 0.60)	8.9 (6.5; 14.2)	0.05 (0.04; 0.08)	0.12	0.48 (-0.02; 0.77)	5.4 (3.9; 8.4)	0.03 (0.02; 0.05)	0.08
	V_avg_ (m·s^−1^)	0.87 (0.66; 0.95)	2.3 (1.7; 3.7)	0.03 (0.02; 0.05)	0.08	0.84 (0.60; 0.93)	2.9 (2.1; 4.5)	0.04 (0.03; 0.06)	0.10	0.96 (0.90; 0.99)	1.4 (1.0; 2.2)	0.02 (0.02; 0.03)	0.05
	Acc_avg_ (m·s^−2^)	0.75 (0.40; 0.90)	8.5 (6.1; 13.7)	0.61 (0.45; 0.96)	1.42	0.80 (0.52; 0.92)	7.3 (5.3; 11.5)	0.54 (0.40; 0.83)	1.25	0.80 (0.53; 0.92)	7.0 (5.1; 11.0)	0.47 (0.35; 0.73)	1.10
	HAF_avg_ (N)	0.83 (0.57; 0.93)	7.9 (5.7; 12.7)	51.1 (37.4; 80.6)	119	0.88 (0.70; 0.95)	6.4 (4.7; 10.1)	40.8 (30.2. 63.2)	95.0	0.88 (0.70; 0.95)	6.3 (4.6; 9.9)	38.7 (28.6; 59.9)	90.0
	HAI_avg_ (N·s)	0.98 (0.95; 0.99)	1.7 (1.2; 2.6)	3.66 (2.68; 5.78)	8.52	0.97 (0.93; 0.99)	2.1 (1.5; 3.2)	4.40 (3.25; 6.82)	10.2	0.99 (0.97; 1.00)	1.5 (1.1; 2.4)	3.11 (2.30; 4.81)	7.23
	HAP_avg_ (W)	0.89 (0.71; 0.96)	7.6 (5.5; 12.2)	62.1 (45.4; 97.9)	144	0.95 (0.85; 0.98)	5.2 (3.8. 8.2)	42.8 (31.6; 66.2)	99.5	0.92 (0.78; 0.97)	6.6 (4.8; 10.3)	52.3 (38.7; 81.0)	122
Late re-acceleration	Time (s)	0.95 (0.85; 0.98)	2.3 (1.7; 3.6)	0.02 (0.02; 0.03)	0.05	0.92 (0.79; 0.97)	2.5 (1.8; 3.9)	0.02 (0.02; 0.04)	0.05	0.96 (0.89; 0.98)	1.7 (1.2; 2.6)	0.02 (0.01; 0.03)	0.04
	Dist (m)	0.73 (0.36; 0.89)	1.7 (1.3; 2.7)	0.07 (0.05; 0.11)	0.16	0.53 (0.04; 0.79)	2.1 (1.6; 3.3)	0.09 (0.06; 0.13)	0.20	0.80 (0.53; 0.92)	1.2 (0.9; 1.9)	0.05 (0.04; 0.08)	0.12
	V_avg_ (m·s^−1^)	0.95 (0.85; 0.98)	1.4 (1.1; 2.3)	0.06 (0.04; 0.10)	0.14	0.93 (0.80. 0.97)	1.6 (1.2; 2.5)	0.07 (0.05; 0.10)	0.15	0.96 (0.90; 0.99)	1.2 (0.9; 1.9)	0.05 (0.04; 0.08	0.11
	Acc_avg_ (m·s^−2^)	0.94 (0.84; 0.98)	3.3 (2.4; 5.3)	0.09 (0.07; 0.15)	0.22	0.92 (0.79; 0.97)	3.8 (2.8; 5.9)	0.10 (0.07; 0.15)	0.23	0.96 (0.90; 0.99)	2.8 (2.1; 4.4)	0.07 (0.05; 0.11)	0.16
	HAF_avg_ (N)	0.97 (0.91; 0.99)	2.7 (2.0; 4.4)	7.73 (5.66; 12.2)	18.0	0.96 (0.88; 0.98)	3.2 (2.4; 5.0)	8.59 (6.34; 13.3)	20.0	0.98 (0.95; 0.99)	2.3 (1.7; 3.6)	6.04 (4.46; 9.34)	14.0
	HAI_avg_ (N·s)	0.99 (0.97; 1.00)	1.0 (0.7; 1.6)	2.61 (1.91; 4.11)	6.07	0.99 (0.96; 0.99)	1.3 (0.9; 2.0)	3.23 (2.39; 5.00)	7.51	0.99 (0.97; 1.00)	1.1 (0.8; 1.7)	2.80 (2.07; 4.34)	6.52
	HAP_avg_ (W)	0.96 (0.88; 0.98)	4.0 (2.9; 6.3)	44.7 (32.8; 70.5)	104	0.94 (0.85; 0.98)	4.7 (3.4; 7.3)	48.7 (36.0; 75.4)	113	0.97 (0.93; 0.99	3.5 (2.6; 5.4)	34.8 (25.7; 53.9)	80.9

Definition and description of all outcome variables are presented in Table 1. ICC, intra-class correlation coefficient; CI, confidence interval; CV, coefficient of variation; TE, typical error; MDC, minimal detectable change; *n*^1^, number of participants for overall time and all re-acceleration outcome variables; *n*^2^, number of participants for all phase 1a outcome variables (i.e., overall, initial acceleration and all deceleration phase outcome variables).

For the m505R test (Table 6), when combining all three comparisons, 76.6% (131 out of 171) of all the variables displayed good or better relative reliability, with ICC values ranging from 0.31 to 0.996. Specifically, 46.8% (80 out of 171) of all variables displayed excellent, 29.8% (51 out of 171) displayed good, 19.3% (33 out of 171) displayed moderate, and 4.1% (7 out of 171) displayed poor relative reliability. With regard to the CV values, 99.4% (170 out of 171) of all variables had acceptable or better absolute reliability, with a range from 0.7 to 15.7%. Specifically, 50.3% (86 out of 171) of all variables displayed good, 49.1% (84 out of 171) displayed moderate, and 0.6% (1 out of 171) displayed very poor absolute reliability*.* Furthermore, the CV values for the comparison of sessions 1–2 ranged from 1.3 to 15.7 (25/57 good), while the CV values for the comparison of sessions 3–4 ranged from 0.7 to 10.0 (31/57 good).

**Table 6:** Test–retest reliability of overall and phase-specific outcome measurement for the m505 test with the right foot ultimate step.

Phase	Outcome variables	Test session 1–2 (*n*^1^ = 19; *n*^2^ = 21)				Test session 2–3 (*n*^1^ = 18; *n*^2^ = 20)				Test session 3–4 (*n*^1^ = 16; *n*^2^ = 19)			
		ICC (95% CI)	CV (95% CI)	TE (95% CI)	MDC_90%_	ICC (95% CI)	CV (95% CI)	TE (95% CI)	MDC_90%_	ICC (95% CI)	CV (95% CI)	TE (95% CI)	MDC_90%_
Overall	Time (s)	0.90 (0.77; 0.96)	2.7 (2.0; 4.0)	0.08 (0.06; 0.12)	0.18	0.94 (0.84; 0.98)	1.9 (1.4; 2.9)	0.06 (0.04; 0.08)	0.13	0.95 (0.87; 0.98)	1.6 (1.2; 2.6)	0.05 (0.04; 0.07)	0.11
Phase 1a	Time (s)	0.86 (0.69; 0.94)	3.4 (2.6; 5.0)	0.05 (0.04; 0.08)	0.13	0.89 (0.75; 0.96)	2.6 (2.0; 3.8)	0.04 (0.03; 0.06)	0.10	0.87 (0.69; 0.95)	2.8 (2.1; 4.2)	0.05 (0.03; 0.07)	0.11
	Dist (m)	0.62 (0.27; 0.83)	2.3 (1.8; 3.4)	0.11 (0.09; 0.16)	0.26	0.68 (0.35; 0.86)	1.4 (1.1; 2.1)	0.07 (0.05; 0.10)	0.16	0.40 (-0.06; 0.71)	2.0 (1.5; 3.0)	0.10 (0.07; 0.14)	0.22
	V_avg_ (m·s^−1^)	0.81 (0.60; 0.92)	3.3 (2.6; 4.9)	0.10 (0.07; 0.14)	0.22	0.89 (0.75; 0.96)	2.4 (1.8; 3.5)	0.07 (0.05; 0.10	0.16	0.86 (0.67; 0.94)	2.5 (1.9; 3.7)	0.07 (0.05; 0.11)	0.17
	V_max_ (m·s^−1^)	0.94 (0.86; 0.98)	1.3 (1.0; 1.9)	0.06 (0.05; 0.09)	0.14	0.94 (0.86; 0.98)	1.4 (1.0; 2.0)	0.06 (0.05; 0.09)	0.14	0.83 (0.62; 0.93)	2.0 (1.5; 2.9)	0.09 (0.07; 0.13)	0.21
Initial acceleration	Time (s)	0.77 (0.52; 0.90)	7.8 (5.9; 11.5)	0.06 (0.05; 0.09)	0.15	0.80 (0.56; 0.91)	6.1 (4.6; 9.1)	0.06 (0.04; 0.08)	0.13	0.96 (0.91; 0.99	2.6 (2.0; 3.9)	0.02 (0.02; 0.03)	0.05
	Dist (m)	0.62 (0.26; 0.82)	6.1 (4.7; 9.0)	0.16 (0.12; 0.23)	0.38	0.50 (0.09; 0.77)	6.5 (4.9; 9.5)	0.17 (0.13; 0.25)	0.39	0.77 (0.50; 0.90)	3.5 (2.6; 5.2)	0.09 (0.07; 0.14)	0.22
Deceleration	Time (s)	0.67 (0.35; 0.85)	6.3 (4.8; 9.2)	0.04 (0.03; 0.06)	0.10	0.75 (0.47; 0.89)	5.5 (4.2; 8.2)	0.04 (0.03; 0.06)	0.09	0.77 (0.50; 0.90)	4.9 (3.7; 7.4)	0.04 (0.03; 0.05)	0.08
	Dist (m)	0.34 (-0.10; 0.67)	8.6 (6.5; 12.7)	0.16 (0.13; 0.24)	0.38	0.49 (0.07; 0.76)	7.5 (5.6; 11.1)	0.15 (0.11; 0.22)	0.35	0.62 (0.25; 0.83)	5.8 (4.4; 8.8)	0.12 (0.09; 0.18)	0.28
	Dec_avg_ (m·s^−2^)	0.78 (0.53; 0.90)	6.1 (4.6; 9.0)	0.37 (0.29; 0.54)	0.87	0.84 (0.63; 0.93)	5.7 (4.3; 8.5)	0.35 (0.27; 0.51)	0.82	0.86 (0.67; 0.94)	5.4 (4.0; 8.0)	0.32 (0.24; 0.47)	0.73
	Dec_max_ (m·s^−2^)	0.72 (0.43; 0.88)	8.1 (6.2; 11.9)	0.83 (0.63; 1.19)	1.92	0.82 (0.61; 0.93)	5.1 (3.9; 7.5)	0.59 (0.45; 0.86)	1.37	0.81 (0.58; 0.92)	5.4 (4.0. 8.0)	0.63 (0.47; 0.93)	1.46
	Time to Dec_max_ (s)	0.73 (0.45; 0.88)	8.5 (6.5; 12.6)	0.05 (0.04; 0.08)	0.13	0.73 (0.43; 0.88)	8.5 (6.4; 12.7)	0.05 (0.04; 0.07)	0.12	0.85 (0.64; 0.94)	5.2 (3.9. 7.8)	0.03 (0.02; 0.05)	0.08
	Dist to Dec_max_ (m)	0.65 (0.31; 0.80)	2.1 (1.6; 3.0)	0.10 (0.08; 0.15)	0.24	0.68 (0.35; 0.86)	1.3 (1.0; 2.0)	0.06 (0.05; 0.09)	0.15	0.40 (-0.05; 0.72)	1.9 (1.4; 2.8)	0.09 (0.07; 0.13)	0.21
	HBF_avg_ (N)	0.89 (0.75; 0.95)	5.7 (4.3; 8.3)	28.5 (21.8; 41.2)	66.3	0.93 (0.83; 0.97)	5.2 (4.0; 7.8)	26.4 (20.1; 38.6)	61.4	0.93 (0.84; 0.97)	5.1 (3.8; 7.6)	25.2 (19.0; 37.2)	58.5
	HBF_max_ (N)	0.85 (0.66; 0.94)	7.8 (5.9; 11.4)	66.3 (50.7; 95.8)	154	0.92 (0.82; 0.97)	5.1 (3.9; 7.6)	47.8 (36.3; 69.7)	111	0.91 (0.79. 0.96)	5.3 (4.0; 7.9)	52.1 (39.4; 77.0)	121
	HBI_avg_ (N·s)	0.99 (0.97; 1.00)	1.4 (1.1; 2.0)	5.02 (3.84; 7.25)	11.7	0.99 (0.97; 1.00)	1.3 (1.0; 1.9)	4.69 (3.57; 6.85)	10.9	0.98 (0.94; 0.99)	1.9 (1.4. 2.7)	6.85 (5.18; 10.1)	15.9
	HBP_avg_ (W)	0.92 (0.82; 0.97)	5.7 (4.4; 8.4)	64.3 (49.2; 92.9)	150	0.93 (0.84; 0.97)	5.9 (4.5; 8.7)	67.1 (51.0; 98.0)	156	0.93 (0.83; 0.97)	6.0 (4.5; 9.0)	68.2 (51.5; 101)	159
	HBP_max_ (W)	0.94 (0.86; 0.97)	7.5 (5.7; 11.1)	136 (104; 196)	315	0.95 (0.89; 0.98)	5.9 (4.5; 8.8)	116 (88.4; 170)	271	0.95 (0.87; 0.98)	6.1 (4.6; 9.1)	122 (92.5; 181)	285
Early deceleration	Time (s)	0.61 (0.25; 0.82)	7.7 (5.8; 11.2)	0.04 (0.03; 0.06)	0.09	0.68 (0.35; 0.86)	7.4 (5.6; 11.1)	0.04 (0.03; 0.06)	0.09	0.78 (0.52; 0.91)	5.4 (4.1; 8.1)	0.03 (0.02; 0.04)	0.07
	Dist (m)	0.31 (-0.13; 0.65)	9.0 (6.8; 13.3)	0.16 (0.12; 0.23)	0.37	0.45 (0.02; 0.74)	8.0 (6.0; 11.9)	0.15 (0.11; 0.22)	0.34	0.65 (0.28; 0.84)	5.9 (4.4; 8.8)	0.11 (0.08; 0.17)	0.26
	V_avg_ (m·s^−1^)	0.94 (0.85; 0.97)	1.7 (1.3; 2.4)	0.06 (0.05; 0.09)	0.15	0.96 (0.89; 0.98)	1.4 (1.0; 2.0)	0.05 (0.04; 0.07)	0.12	0.89 (0.74; 0.96)	1.8 (1.3; 2.6)	0.07 (0.05; 0.10)	0.16
	Dec_avg_ (m·s^−2^)	0.70 (0.39; 0.87)	7.8 (5.9; 11.4)	0.33 (0.25; 0.47)	0.76	0.76 (0.48; 0.89)	8.0 (6.0; 11.9)	0.33 (0.25; 0.48)	0.77	0.85 (0.66; 0.94)	6.1 (4.6; 9.2)	0.24 (0.18; 0.36)	0.56
	HBF_avg_ (N)	0.84 (0.64; 0.93)	7.2 (5.4; 10.5)	24.8 (18.9; 35.7)	57.6	0.88 (0.72; 0.95)	7.3 (5.5; 10.8)	24.7 (18.8; 36.1)	57.6	0.93 (0.82; 0.97)	5.7 (4.3; 8.5)	19.1 (14.4; 28.2)	44.3
	HBI_avg_ (N·s)	0.98 (0.96; 0.99)	1.5 (1.1; 2.2)	2.68 (2.05; 3.87)	6.23	0.99 (0.97; 1.00)	1.3 (1.0; 1.9)	2.35 (1.79; 3.43)	5.47	0.98 (0.94; 0.99)	1.8 (1.4; 2.7)	3.36 (2.54; 4.96)	7.81
	HBP_avg_ (W)	0.88 (0.73; 0.95)	7.3 (5.6; 10.7)	84.6 (64.8; 122)	197	0.89 (0.75; 0.96)	7.9 (6.0; 11.8)	91.2 (69.4; 133)	212	0.92 (0.81; 0.97)	6.7 (5.1; 10.1)	77.5 (58.5; 115)	180
Late deceleration	Time (s)	0.61 (0.26; 0.82)	9.0 (6.8; 13.3)	0.02 (0.02; 0.03)	0.05	0.69 (0.37; 0.87)	6.0 (4.5; 8.8)	0.01 (0.01; 0.02)	0.03	0.75 (0.45; 0.89)	5.6 (4.2; 8.5)	0.01 (0.01; 0.02)	0.02
	Dist (m)	0.57 (0.19; 0.80)	15.7 (11.8; 23.5)	0.02 (0.02; 0.03)	0.06	0.67 (0.33;0.85)	9.5 (7.2; 14.2)	0.01 (0.01; 0.02)	0.03	0.67 (0.32; 0.86)	10.0 (7.4; 15.1)	0.01 (0.01; 0.02)	0.03
	V_avg_ (m·s^−1^)	0.84 (0.65; 0.93)	1.9 (1.5; 2.8)	0.02 (0.02; 0.03)	0.05	0.83 (0.62; 0.93)	2.0 (1.6; 3.0)	0.02 (0.02; 0.03)	0.05	0.85 (0.65; 0.94)	1.8 (1.4; 2.7)	0.02 (0.02; 0.03)	0.05
	Dec_avg_ (m·s^−2^)	0.75 (0.47; 0.89)	8.6 (6.5; 12.7)	0.83 (0.64; 1.20)	1.94	0.81 (0.59; 0.92)	5.6 (4.3; 8.3)	0.62 (0.47; 0.91)	1.45	0.84 (0.64; 0.94)	5.2 (3.9; 7.8)	0.59 (0.44; 0.87)	1.36
	HBF_avg_ (N)	0.86 (0.69; 0.94)	8.2 (6.2; 12.1)	65.7 (50.2; 94.8)	153	0.92 (0.81; 0.97)	5.3 (4.0; 7.9)	48.0 (36.5; 70.1)	112	0.92 (0.82; 0.97)	5.0 (3.8; 7.5)	47.9 (36.2; 70.8)	111
	HBI_avg_ (N·s)	0.99 (0.97; 0.99)	1.5 (1.2; 2.2)	2.70 (2.10; 3.90)	6.3	0.99 (0.97; 0.99)	1.5 (1.2; 2.2)	2.60 (2.00; 3.80)	6.0	0.97 (0.93; 0.99)	2.0 (1.5; 3.0)	3.67 (2.77; 5.43)	8.5
	HBP_avg_ (W)	0.90 (0.76; 0.96)	8.0 (6.1; 11.8)	72.5 (55.5; 105)	169	0.94 (0.85; 0.97)	5.5 (4.1; 8.1)	58.0 (44.1; 84.7)	135	0.93 (0.84; 0.97)	5.6 (4.2; 8.4)	59.8 (45.2; 88.4)	139
Re-acceleration	Time (s)	0.92 (0.80; 0.97)	2.7 (2.0; 4.0)	0.04 (0.03; 0.05)	0.09	0.97 (0.93; 0.99)	1.3 (1.0; 2.0)	0.02 (0.01; 0.03)	0.05	0.95 (0.87; 0.98)	2.0 (1.5; 3.2)	0.03 (0.02; 0.04)	0.06
	Dist (m)	0.73 (0.42; 0.89)	1.9 (1.4; 2.8)	0.09 (0.07; 0.13)	0.21	0.70 (0.37. 0.87)	1.5 (1.1; 2.2)	0.07 (0.05; 0.10)	0.16	0.80 (0.53; 0.92)	1.2 (0.9; 1.8)	0.06 (0.04; 0.09)	0.13
	V_avg_ (m·s^−1^)	0.88 (0.72; 0.95)	2.2 (1.6; 3.2)	0.07 (0.05; 0.11)	0.17	0.96 (0.90; 0.98)	1.2 (0.9; 1.8)	0.04 (0.03. 0.06)	0.10	0.97 (0.91; 0.99)	1.2 (0.9; 1.8)	0.04 (0.03; 0.06)	0.09
	V_max_ (m·s^−1^)	0.90 (0.76; 0.96)	2.2 (1.7; 3.3)	0.11 (0.08; 0.16)	0.26	0.97 (0.91; 0.99)	1.3 (1.0; 2.0)	0.06 (0.05; 0.10)	0.15	0.98 (0.93; 0.99)	1.1 (0.8; 1.7)	0.05 (0.04; 0.08)	0.12
	Acc_avg_ (m·s^−2^)	0.92 (0.80; 0.97)	4.4 (3.3; 6.5)	0.16 (0.12; 0.23)	0.37	0.99 (0.96; 0.99)	1.8 (1.4; 2.7)	0.06 (0.05; 0.09)	0.15	0.97 (0.92; 0.99)	2.4 (1.8; 3.7)	0.09 (0.07; 0.14)	0.21
	Acc_max_ (m·s^−2^)	0.66 (0.30; 0.85)	8.7 (6.5; 13.2)	0.85 (0.64; 1.25)	1.97	0.58 (0.17; 0.82)	8.8 (6.6; 13.5)	0.88 (0.66; 1.32)	2.04	0.78 (0.48; 0.91)	6.0 (4.4; 9.4)	0.66 (0.49; 1.02)	1.53
	HAF_avg_ (N)	0.95 (0.88; 0.98)	3.8 (2.9; 5.7)	13.7 (10.3; 20.2)	31.8	0.99 (0.98; 1.00)	1.7 (1.2; 2.5)	5.29 (3.97; 7.93)	12.3	0.98 (0.96; 0.99)	2.1 (1.5; 3.2)	7.99 (5.90; 12.4)	18.6
	HAF_max_ (N)	0.79 (0.53; 0.91)	8.3 (6.2; 12.6)	67.3 (50.9; 99.6)	17	0.79 (0.52; 0.91)	8.3 (6.2; 12.7)	70.4 (52.8; 106)	164	0.99 (0.71; 0.95)	5.7 (4.2; 9.0)	54.3 (40.1; 84.0)	126
	HAI_avg_ (N·s)	0.98 (0.94; 0.99)	1.8 (1.3; 2.7)	8.69 (6.57; 12.9)	20.2	0.99 (0.97; 1.00)	1.2 (0.9; 1.8)	5.65 (4.24; 8.47)	13.2	0.99 (0.98; 1.00)	1.0 (0.7; 1.5)	4.30 (3.20; 6.71)	10.1
	HAP_avg_ (W)	0.93 (0.83; 0.97)	5.6 (4.2; 8.4)	54.1 (40.9; 80.0)	126	0.99 (0.97; 1.00)	2.5 (1.9; 3.8)	21.3 (16.0; 31.9)	49.5	0.99 (0.96; 0.99)	2.6 (1.9; 4.1)	25.3 (18.7; 39.2)	58.9
	HAP_max_ (W)	0.87 (0.69; 0.95)	7.5 (5.7; 11.4)	103 (77.4; 152)	238	0.93 (0.83; 0.97)	6.1 (4.5; 9.3)	73.6 (55.2; 110)	171	0.96 (0.89; 0.98)	5.0 (3.7; 7.9)	57.6 (42.5; 89.1)	134
Early re-acceleration	Time (s)	0.73 (0.42; 0.88)	7.3 (5.5; 11.0)	0.03 (0.02; 0.04)	0.06	0.77 (0.49; 0.90)	6.3 (4.7; 9.6)	0.02 (0.02; 0.04)	0.05	0.64 (0.21; 0.85)	8.0 (5.8; 12.6)	0.03 (0.02; 0.04)	0.07
	Dist (m)	0.66 (0.31; 0.86)	6.7 (5.0; 10.0)	0.04 (0.03; 0.06)	0.10	0.72 (0.40; 0.88)	5.6 (4.2; 8.5)	0.04 (0.03; 0.05)	0.08	0.40 (-0.13; 0.73)	8.7 (6.3; 13.7)	0.05 (0.04; 0.08)	0.12
	V_avg_ (m·s^−1^)	0.86 (0.66; 0.94)	2.3 (1.8; 3.5)	0.03 (0.02; 0.05)	0.08	0.88 (0.72; 0.95)	2.0 (1.5; 3.0)	0.03 (0.02; 0.04)	0.07	0.82 (0.57; 0.93)	3.0 (2.2; 4.7)	0.04 (0.03; 0.06)	0.10
	Acc_avg_ (m·s^−2^)	0.82 (0.60; 0.93)	7.8 (5.9; 11.8)	0.50 (0.38; 0.74)	1.17	0.90 (0.76; 0.96)	5.7 (4.3; 8.7)	0.35 (0.27; 0.53)	0.82	0.77 (0.46; 0.91)	7.8 (5.7; 12.4)	0.53 (0.39; 0.83)	1.24
	HAF_avg_ (N)	0.87 (0.69; 0.95)	7.2 (5.4; 10.9)	42.6 (32.2; 63.0)	99.0	0.94 (0.86; 0.98)	5.2 (3.9; 7.9)	27.8 (20.9; 41.7)	64.8	0.87 (0.66; 0.95)	7.1 (5.2; 11.2)	43.0 (31.8; 66.6)	100
	HAI_avg_ (N·s)	0.97 (0.94; 0.99)	2.1 (1.6; 3.1)	4.51 (3.40; 6.66)	10.5	0.99 (0.96; 0.99)	1.7 (1.3; 2.5)	3.49 (2.62; 5.23)	8.12	0.99 (0.97; 1.00)	1.5 (1.1; 2.3)	2.97 (2.19; 4.59)	6.90
	HAP_avg_ (W)	0.88 (0.72; 0.95)	8.1 (6.1; 12.3)	62.2 (47.0; 92.0)	145	0.97 (0.92; 0.99)	4.8 (3.6; 7.2)	33.4 (25.0; 50.0)	77.6	0.92 (0.79; 0.97)	6.4 (4.7; 10.1)	52.2 (38.5; 80.7)	121
Late re-acceleration	Time (s)	0.92 (0.80; 0.97)	2.7 (2.1; 4.1)	0.03 (0.02; 0.04)	0.07	0.95 (0.87; 0.98)	2.2 (1.7: 3.4)	0.02 (0.02; 0.03)	0.05	0.96 (0.89; 0.98)	2.0 (1.5; 3.1)	0.02 (0.01; 0.03)	0.04
	Dist (m)	0.66 (0.30; 0.85)	2.2 (1.7; 3.3)	0.10 (0.07; 0.14)	0.22	0.67 (0.31; 0.86)	1.7 (1.3; 2.6)	0.07 (0.05; 0.11)	0.17	0.80 (0.52; 0.92)	1.4 (1.0; 2.1)	0.06 (0.04; 0.09)	0.13
	V_avg_ (m·s^−1^)	0.92 (0.80; 0.97)	1.9 (1.4; 2.8)	0.08 (0.06; 0.11)	0.18	0.96 (0.89; 0.98)	1.4 (1.0; 2.1)	0.06 (0.04; 0.08)	0.13	0.97 (0.92; 0.99)	1.2 (0.9; 1.8)	0.05 (0.03. 0.07)	0.11
	Acc_avg_ (m·s^−2^)	0.92 (0.82; 0.97)	4.5 (3.4; 6.8)	0.11 (0.08; 0.16)	0.26	0.97 (0.91; 0.99)	3.1 (2.3; 4.6)	0.07 (0.05; 0.11)	0.17	0.97 (0.91; 0.99)	2.8 (2.0; 4.3)	0.07 (0.05; 0.11)	0.16
	HAF_avg_ (N)	0.96 (0.89; 0.98)	3.7 (2.8; 5.5)	9.40 (7.10; 13.9)	21.9	0.99 (0.98; 1.00)	2.5 (1.9; 3.8)	5.86 (4.40; 8.79)	13.6	1.00 (0.99; 1.00)	2.1 (1.6; 3.3)	5.57 (4.12; 8.62)	13.0
	HAI_avg_ (N·s)	0.97 (0.93; 0.99)	1.7 (1.3; 2.5)	4.49 (3.39; 6.64)	10.4	0.99 (0.98; 1.00)	1.0 (0.7; 1.4)	2.52 (1.89; 3.78)	5.87	1.00 (0.99; 1.00)	0.7 (0.5; 1.1)	1.79 (1.32; 2.77)	4.17
	HAP_avg_ (W)	0.94 (0.85; 0.98)	5.6 (4.2; 8.4)	56.4 (42.6; 83.4)	131	0.98 (0.95; 0.99)	3.6 (2.7; 5.4)	31.7 (23.8; 47.5)	73.8	0.98 (0.95; 0.99)	3.1 (2.2; 4.8)	29.5 (21.8; 45.7)	68.6

Definition and description of all outcome variables are presented in Table 1. ICC, intra-class correlation coefficient; CI, confidence interval; CV, coefficient of variation; TE, typical error; MDC, minimal detectable change; *n*^1^, number of participants for overall time and all re-acceleration outcome variables; *n*^2^, number of participants for all phase 1a outcome variables (i.e., overall, initial acceleration and all deceleration phase outcome variables).

### 3.2. 105 test

For the 105L test (Table 7), when combining all three comparisons, 77.2% (132 out of 171) of all variables displayed good or better relative reliability, with ICC values ranging from 0.34 to 0.99. Specifically, 50.9% (87 out of 171) of all variables displayed excellent, 26.3% (45 out of 171) good, 17.0% (29 out of 171) displayed moderate, and 5.6% (10 out of 171) displayed poor relative reliability*.* With regard to the CV values, 95.3% (163 out of 171) of all variables had acceptable or better absolute reliability, with a range from 0.7 to 12.2%. Specifically, 57.3% (98 out of 171) of all variables displayed good, 38.0% (65 out of 171) displayed acceptable, and 4.7% (8 out of 171) displayed poor absolute reliability. Consequently, none of the variables displayed very poor absolute reliability. Furthermore, the CV values for the comparison of sessions 1–2 ranged from 0.8 to 12.2 (27/57 good), while the CV values for the comparison of sessions 3–4 ranged from 0.7 to 8.2 (41/57 good).

**Table 7:** Test–retest reliability of overall and phase-specific outcome measurement for the 10-0-5 test with the left foot ultimate step.

Phase	Outcome variables	Test session 1–2 (*n*^1^ = 19; *n*^2^ = 20)				Test session 2–3 (*n*^1^ = 19; *n*^2^ = 20)				Test session 3–4 (*n*^1^ = 18; *n*^2^ = 19)			
		ICC (95% CI)	CV (95% CI)	TE (95% CI)	MDC_90%_	ICC (95% CI)	CV (95% CI)	TE (95% CI)	MDC_90%_	ICC (95% CI)	CV (95% CI)	TE (95% CI)	MDC_90%_
Overall	Time (s)	0.88 (0.73; 0.95)	2.6 (2.0; 3.8)	0.10 (0.08; 0.15)	0.23	0.96 (0.90; 0.99)	1.4 (1.0; 2.1)	0.05 (0.04; 0.08)	0.12	0.94 (0.86; 0.98)	1.6 (1.2; 2.3)	0.06 (0.04; 0.09)	0.14
Phase 1a	Time (s)	0.87 (0.71; 0.95)	2.7 (2.0; 3.9)	0.07 (0.05; 0.10)	0.16	0.92 (0.82; 0.97)	1.8 (1.4; 2.7)	0.05 (0.04; 0.07)	0.11	0.92 (0.81; 0.97)	1.7 (1.3; 2.5)	0.04 (0.03; 0.06)	0.10
	Dist (m)	0.46 (0.02; 0.74)	0.8 (0.6; 1.1)	0.08 (0.06; 0.11)	0.18	0.35 (-0.11; 0.68)	1.0 (0.7; 1.4)	0.10 (0.07; 0.14)	0.22	0.69 (0.35; 0.86)	0.8 (0.6; 1.1)	0.08 (0.06; 0.11)	0.18
	V_avg_ (m·s^−1^)	0.86 (0.68; 0.94)	2.6 (2.0; 3.8)	0.10 (0.07; 0.14)	0.23	0.89 (0.75; 0.95)	2.1 (1.6; 3.1)	0.08 (0.06; 0.11)	0.18	0.87 (0.70; 0.95)	1.9 (1.5; 2.9)	0.07 (0.06; 0.11)	0.17
	V_max_ (m·s^−1^)	0.95 (0.88; 0.98)	1.4 (1.1; 2.1)	0.08 (0.06; 0.12)	0.20	0.95 (0.89; 0.98)	1.3 (1.0; 1.9)	0.08 (0.06; 0.11)	0.18	0.94 (0.86; 0.98)	1.3 (1.0; 2.0)	0.08 (0.06; 0.12)	0.18
Initial acceleration	Time (s)	0.82 (0.59; 0.92)	5.1 (3.9; 7.5)	0.08 (0.06; 0.11)	0.17	0.92 (0.80; 0.97)	3.3 (2.5; 4.9)	0.05 (0.04; 0.07)	0.11	0.93 (0.82; 0.97)	3.0 (2.3; 4.5)	0.04 (0.03; 0.06	0.10
	Dist (m)	0.61 (0.24; 0.82)	4.3 (3.3; 6.4)	0.25 (0.19; 0.36)	0.57	0.74 (0.45; 0.89)	4.0 (3.0; 5.9)	0.23 (0.17; 0.34)	0.53	0.86 (0.67; 0.94)	2.9 (2.2; 4.2)	0.17 (0.13; 0.25)	0.39
Deceleration	Time (s)	0.68 (0.35; 0.86)	5.0 (3.8; 7.4)	0.05 (0.04; 0.08)	0.12	0.65 (0.30; 0.84)	4.7 (3.6; 6.9)	0.05 (0.04; 0.08)	0.12	0.82 (0.60; 0.93)	3.9 (2.9; 5.8)	0.04 (0.03; 0.06)	0.09
	Dist (m)	0.58 (0.20; 0.81)	6.7 (5.0; 9.9)	0.25 (0.19; 0.37)	0.58	0.68 (0.35; 0.86)	5.7 (4.3; 8.5)	0.22 (0.17; 0.32)	0.51	0.77 (0.51; 0.91)	4.8 (3.6; 7.3)	0.18 (0.13; 0.26)	0.41
	Dec_avg_ (m·s^−2^)	0.82 (0.60; 0.92)	5.6 (4.2; 8.2)	0.29 (0.22; 0.42)	0.67	0.83 (0.61; 0.93)	5.5 (4.2; 8.1)	0.27 (0.21; 0.40)	0.63	0.84 (0.64; 0.93)	4.7 (3.5; 7.0)	0.27 (0.20; 0.40)	0.62
	Dec_max_ (m·s^−2^)	0.62 (0.25; 0.83)	8.9 (6.7; 13.3)	0.92 (0.70; 1.34)	2.14	0.88 (0.72; 0.95)	5.0 (3.8; 7.5)	0.50 (0.38; 0.73)	1.16	0.89 (0.74; 0.96)	3.9 (2.9; 5.8)	0.48 (0.36; 0.70)	1.11
	Time to Dec_max_ (s)	0.59 (0.21; 0.81)	8.4 (6.3; 12.5)	0.08 (0.06; 0.12)	0.19	0.64 (0.28; 0.84)	7.8 (5.9; 11.6)	0.08 (0.06; 0.11)	0.18	0.89 (0.74; 0.96)	4.6 (3.5; 6.9)	0.04 (0.03; 0.06)	0.10
	Dist to Dec_max_ (m)	0.39 (-0.06; 0.70)	0.8 (0.6; 1.2)	0.08 (0.06; 0.11)	0.18	0.34 (-0.11; 0.67)	0.9 (0.6; 1.2)	0.08 (0.06; 0.12)	0.19	0.66 (0.30; 0.85)	0.7 (0.5; 1.1)	0.07 (0.05. 0.10)	0.16
	HBF_avg_ (N)	0.93 (0.83; 0.97)	5.3 (4.0; 7.9)	22.7 (17.3; 33.2)	52.8	0.92 (0.82; 0.97)	5.5 (4.1; 8.1)	23.1 (17.6; 33.8)	53.8	0.94 (0.85; 0.98)	4.5 (3.4; 6.7)	21.1 (16.0; 31.2)	49.2
	HBF_max_ (N)	0.83 (0.61; 0.93)	8.6 (6.5; 12.8)	69.0 (52.5; 101)	160	0.94 (0.86; 0.98)	5.2 (3.9; 7.6)	39.2 (29.8; 57.3)	91.2	0.94 (0.86; 0.98)	3.9 (3.0; 5.9)	39.2 (29.6; 57.9)	91.1
	HBI_avg_ (N·s)	0.99 (0.97; 1.00)	1.4 (1.1; 2.1)	6.87 (5.22; 10.0)	16.0	0.99 (0.97; 1.00)	1.4 (1.1; 2.1)	6.84 (5.20; 9.99)	15.9	0.99 (0.97; 1.00)	1.4 (1.1; 2.1)	6.78 (5.12; 10.0)	15.8
	HBP_avg_ (W)	0.94 (0.85; 0.97)	6.1 (4.6; 9.1)	77.8 (59.1; 114)	181	0.93 (0.84; 0.97)	6.3 (4.8; 9.4)	77.8 (59.2; 114)	181	0.94 (0.86; 0.98)	5.4 (4.0; 8.0)	73.7 (55.7; 109)	171
	HBP_max_ (W)	0.85 (0.66; 0.94)	9.1 (6.9; 13.6)	186 (142; 272)	433	0.94 (0.86; 0.98)	6.9 (5.2; 10.3)	120 (91.4; 176)	280	0.95 (0.87; 0.98)	6.5 (4.8; 9.7)	124 (93.7; 183)	288
Early deceleration	Time (s)	0.59 (0.21; 0.81)	6.7 (5.1; 10.0)	0.05 (0.04; 0.08)	0.12	0.67 (0.33; 0.85)	6.2 (4.7; 9.2)	0.05 (0.04; 0.07)	0.11	0.83 (0.61; 0.93)	5.1 (3.8; 7.7)	0.04 (0.03; 0.05)	0.09
	Dist (m)	0.57 (0.18; 0.80)	7.2 (5.4; 10.7)	0.25 (0.19; 0.36)	0.58	0.70 (0.39; 0.87)	6.2 (4.7; 9.1)	0.21 (0.16; 0.31)	0.49	0.78 (0.51; 0.91)	5.3 (4.0; 8.0)	0.18 (0.13; 0.26)	0.41
	V_avg_ (m·s^−1^)	0.92 (0.81; 0.97)	1.8 (1.4; 2.7)	0.09 (0.07; 0.13)	0.21	0.96 (0.91; 0.99)	1.2 (0.9; 1.8)	0.06 (0.04; 0.08)	0.13	0.94 (0.85; 0.98)	1.5 (1.1; 2.2)	0.07 (0.05; 0.11)	0.17
	Dec_avg_ (m·s^−2^)	0.78 (0.52; 0.90)	7.2 (5.4; 10.7)	0.25 (0.19; 0.37)	0.58	0.80 (0.56; 0.91)	6.9 (5.2; 10.3)	0.24 (0.18; 0.35)	0.56	0.80 (0.56; 0.92)	6.0 (4.5; 8.9)	0.23 (0.18; 0.35)	0.54
	HBF_avg_ (N)	0.90 (0.76; 0.96)	6.8 (5.2; 10.1)	19.5 (14.9; 28.6)	45.5	0.90 (0.76; 0.96)	6.7 (5.0; 9.9)	19.8 (15.0; 28.9)	46.0	0.91 (0.78; 0.96)	5.6 (4.2; 8.5)	18.5 (14.0; 27.3)	43.0
	HBI_avg_ (N·s)	0.99 (0.98; 1.00)	1.3 (1.0; 1.9)	3.15 (2.40; 4.60)	7.33	0.99 (0.98; 1.00)	1.2 (0.9; 1.8)	3.01 (2.29; 4.40)	7.01	0.99 (0.98; 1.00)	1.2 (0.9; 1.9)	2.98 (2.25; 4.40)	6.92
	HBP_avg_ (W)	0.92 (0.81; 0.97)	7.6 (5.7; 11.3)	95.3 (72.5; 139)	222	0.91 (0.79; 0.96)	7.6 (5.8; 11.4)	99.9 (75.9; 146)	232	0.92 (0.80; 0.97)	6.6 (4.9; 9.9)	94.0 (71.0; 139)	219
Late deceleration	Time (s)	0.73 (0.43; 0.88)	7.7 (5.8; 11.4)	0.02 (0.02; 0.03)	0.05	0.78 (0.52; 0.90)	6.3 (4.7; 9.3)	0.02 (0.01; 0.03)	0.04	0.91 (0.79; 0.97)	4.1 (3.0; 6.1)	0.01 (0.01; 0.02)	0.03
	Dist (m)	0.72 (0.41; 0.88)	11.2 (8.4; 16.8)	0.04 (0.03; 0.06)	0.09	0.67 (0.34; 0.85)	10.6 (8.0; 15.8)	0.04 (0.03; 0.05)	0.09	0.89 (0.73; 0.95)	6.5 (4.9. 9.8)	0.02 (0.01; 0.03)	0.04
	V_avg_ (m·s^−1^)	0.80 (0.56; 0.91)	3.3 (2.5; 4.9)	0.05 (0.04; 0.07)	0.12	0.86 (0.67; 0.94)	2.8 (2.1; 4.1)	0.04 (0.03; 0.06)	0.10	0.85 (0.65; 0.94)	2.6 (1.9; 3.8)	0.04 (0.03; 0.06)	0.09
	Dec_avg_ (m·s^−2^)	0.76 (0.49; 0.90)	8.0 (6.1; 12.0)	0.75 (0.57; 1.10)	1.75	0.85 (0.65; 0.93)	6.4 (4.8; 9.4)	0.57 (0.43; 0.83)	1.32	0.89 (0.75; 0.96)	4.2 (3.1; 6.2)	0.48 (0.36; 0.71)	1.11
	HBF_avg_ (N)	0.88 (0.71; 0.95)	7.6 (5.7; 11.3)	57.7 (43.8; 84.2)	134	0.91 (0.80; 0.96)	6.3 (4.7; 9.3)	47.4 36.1; 69.2)	110	0.95 (0.87; 0.98)	3.9 (3.0; 5.9)	37.6 (28.4; 55.7)	87.6
	HBI_avg_ (N·s)	0.98 (0.96; 0.99)	1.7 (1.3; 2.5)	4.09 (3.11; 5.97)	9.52	0.98 (0.96; 0.99)	1.7 (1.3; 2.5)	4.04 (3.08; 5.91)	9.41	0.99 (0.96; 0.99)	1.7 (1.3; 2.5)	3.91 (2.96; 5.78)	9.10
	HBP_avg_ (W)	0.89 (0.76; 0.96)	8.1 (6.1; 12.0)	92.6 (70.4; 135)	215	0.93 (0.84; 0.97)	6.6 (5.0; 9.8)	71.8 (54.6; 105)	167	0.95 (0.88; 0.98)	4.5 (3.4; 6.7)	64.3 (48.6; 95.0)	150
Re-acceleration	Time (s)	0.87 (0.70; 0.95)	3.1 (2.4; 4.6)	0.04 (0.03; 0.06)	0.10	0.96 (0.91; 0.99)	1.6 (1.2; 2.4)	0.02 (0.02; 0.03)	0.05	0.94 (0.84; 0.97)	2.2 (1.7; 3.3)	0.03 (0.02; 0.04)	0.07
	Dist (m)	0.44 (0.01; 0.73)	1.6 (1.2; 2.4)	0.08 (0.06; 0.11)	0.18	0.34 (-0.16; 0.68)	2.0 (1.5; 3.0)	0.10 (0.07; 0.14)	0.22	0.67 (0.31; 0.86)	1.6 (1.2; 2.4)	0.08 (0.06; 0.11)	0.18
	V_avg_ (m·s^−1^)	0.81 (0.59; 0.92)	3.0 (2.3; 4.4)	0.10 (0.08; 0.15)	0.23	0.97 (0.92; 0.99)	1.2 (0.9; 1.8)	0.04 (0.03; 0.06)	0.09	0.92 (0.80; 0.97)	1.7 (1.3; 2.6)	0.06 (0.05; 0.09)	0.14
	V_max_ (m·s^−1^)	0.90 (0.77; 0.96)	2.5 (1.9; 3.7)	0.13 (0.10; 0.19)	0.30	0.94 (0.86; 0.98)	2.0 (1.5; 3.0)	0.10 (0.07; 0.15)	0.23	0.95 (0.87; 0.98)	1.8 (1.3; 2.7)	0.09 (0.07; 0.14)	0.21
	Acc_avg_ (m·s^−2^)	0.88 (0.73; 0.95)	5.1 (3.8; 7.5)	0.20 (0.15; 0.29)	0.46	0.97 (0.93; 0.99)	2.5 (1.9; 3.8)	0.09 (0.07; 0.14)	0.22	0.95 (0.87; 0.98)	3.3 (2.4; 4.9)	0.13 (0.10; 0.19)	0.30
	Acc_max_ (m·s^−2^)	0.61 (0.25; 0.83)	12.2 (9.2; 18.3)	1.14 (0.87; 1.67)	2.66	0.86 (0.68; 0.94)	7.9 (5.8; 12.0)	0.69 (0.52; 1.04)	1.61	0.88 (0.72; 0.95)	6.6 (4.9; 10.0)	0.65 (0.49; 0.98)	1.52
	HAF_avg_ (N)	0.94 (0.85; 0.97)	4.5 (3.4; 6.7)	16.1 (12.3; 23.6)	37.5	0.99 (0.96; 0.99)	2.3 (1.7; 3.4)	7.76 (5.82; 11.6)	18.1	0.98 (0.94; 0.99)	2.7 (2.0; 4.0)	9.85 (7.39; 14.8)	22.9
	HAF_max_ (N)	0.75 (0.47; 0.89)	11.3 (8.5; 17.0)	85.1 (64.7; 124)	198	0.91 (0.77; 0.96)	7.5 (5.6; 11.4)	52.9 (39.7; 79.3)	123	0.92 (0.81; 0.97)	5.9 (4.4; 9.0)	49.1 (36.9; 73.7)	114
	HAI_avg_ (N·s)	0.98 (0.94; 0.99)	2.0 (1.6; 3.0)	9.55 (7.26; 14.0)	22.2	0.98 (0.96; 0.99)	1.8 (1.3; 2.6)	8.05 (6.04; 12.1)	18.7	0.98 (0.96; 0.99)	1.6 (1.2; 2.5)	7.85 (5.89; 11.8)	18.3
	HAP_avg_ (W)	0.91 (0.80; 0.97)	6.8 (5.1; 10.1)	66.9 (50.9; 97.8)	156	0.98 (0.95; 0.99)	3.6 (2.7; 5.5)	33.3 (25.0; 49.9)	77.4	0.97 (0.92; 0.99)	4.1 (3.0; 6.2)	40.5 (30.4; 60.7)	94.1
	HAP_max_ (W)	0.92 (0.80; 0.97)	7.3 (5.5; 10.8)	87.9 (66.9; 128)	205	0.93 (0.82; 0.97)	6.6 (4.9; 10.0)	85.9 (64.4; 129)	200	0.91 (0.78; 0.96)	6.6 (4.9; 10.0)	89.5 (67.2; 134)	208
Early re-acceleration	Time (s)	0.47 (0.05; 0.75)	10.1 (7.6; 15.1)	0.04 (0.03; 0.05)	0.09	0.71 (0.38; 0.88)	8.1 (6.0; 12.4)	0.03 (0.02; 0.04)	0.07	0.69 (0.35; 0.87)	7.7 (5.7; 11.7)	0.03 (0.02; 0.04)	0.06
	Dist (m)	0.50 (0.08; 0.76)	8.3 (6.2; 12.3)	0.05 (0.04; 0.08)	0.13	0.61 (0.21; 0.83)	9.0 (6.7; 13.7)	0.05 (0.04; 0.08)	0.13	0.65 (0.28; 0.85)	6.7 (5.0; 10.2)	0.04 (0.03; 0.06)	0.10
	V_avg_ (m·s^−1^)	0.80 (0.57; 0.92)	3.7 (2.8; 5.4)	0.05 (0.04; 0.08)	0.13	0.85 (0.65; 0.94)	3.3 (2.5; 5.0)	0.05 (0.04; 0.07)	0.11	0.92 (0.80; 0.97)	2.5 (1.9; 3.8)	0.04 (0.03; 0.05)	0.08
	Acc_avg_ (m·s^−2^)	0.61 (0.24; 0.82)	11.0 (8.3; 16.5)	0.73 (0.56; 1.07)	1.70	0.87 (0.68; 0.95)	7.1 (5.3; 10.9)	0.43 (0.32; 0.64)	1.00	0.75 (0.45; 0.89)	8.2 (6.1; 12.5)	0.58 (0.43; 0.87)	1.35
	HAF_avg_ (N)	0.74 (0.44; 0.89)	10.1 (7.6; 15.2)	58.2 (44.3; 85.1)	136	0.92 (0.80; 0.97)	6.4 (4.8; 9.7)	33.0 (24.8; 49.5)	76.8	0.85 (0.64; 0.94)	7.4 (5.5; 11.4)	45.3 (34.0; 68.0)	105
	HAI_avg_ (N·s)	0.98 (0.94; 0.99)	2.3 (1.7; 3.3)	4.81 (3.65; 7.02)	11.2	0.98 (0.94; 0.99)	2.4 (1.8; 3.6)	4.82 (3.61; 7.22)	11.2	0.98 (0.96; 0.99)	1.9 (1.4. 2.8)	4.00 (3.00; 5.99)	9.30
	HAP_avg_ (W)	0.78 (0.52. 0.91)	11.6 (8.7; 17.4)	90.7 (68.9; 132)	211	0.97 (0.91; 0.99)	5.6 (4.2; 8.5)	36.3 (27.2; 54.4)	84.4	0.88 (0.71; 0.95)	8.1 (6.1; 12.5)	68.2 (51.2; 102)	159
Late re-acceleration	Time (s)	0.94 (0.85; 0.97)	2.5 (1.9; 3.7)	0.02 (0.02; 0.03)	0.06	0.92 (0.80; 0.97	3.0 (2.2; 4.5)	0.03 (0.02; 0.04)	0.07	0.96 (0.91; 0.99)	1.9 (1.4; 2.9)	0.02 (0.01; 0.03)	0.04
	Dist (m)	0.45 (0.02; 0.74)	1.9 (1.5; 2.8)	0.08 (0.06; 0.11)	0.18	0.41 (-0.08; 0.72)	2.7 (2.0; 4.1)	0.11 (0.08; 0.16)	0.25	0.75 (0.44; 0.89)	1.9 (1.4; 2.8)	0.08 (0.06; 0.11)	0.18
	V_avg_ (m·s^−1^)	0.91 (0.78; 0.96)	2.3 (1.8; 3.4)	0.10 (0.07; 0.14)	0.22	0.95 (0.88; 0.98)	1.7 (1.2; 2.5)	0.07 (0.05; 0.10)	0.15	0.97 (0.92; 0.99)	1.2 (0.9; 1.8)	0.05 (0.04; 0.07)	0.11
	Acc_avg_ (m·s^−2^)	0.92 (0.80; 0.97)	4.7 (3.6; 7.0)	0.13 (0.10; 0.18)	0.29	0.94 (0.85; 0.98)	4.4 (3.3; 6.6)	0.11 (0.08; 0.16)	0.25	0.97 (0.92; 0.99)	2.9 (2.2; 4.4)	0.07 (0.06; 0.11)	0.17
	HAF_avg_ (N)	0.96 (0.89; 0.98)	3.9 (3.0; 5.8)	10.3 (7.82; 15.0)	23.9	0.97 (0.93; 0.99)	3.5 (2.6; 5.2)	8.43 (6.32; 12.6)	19.6	0.99 (0.97; 1.00)	2.1 (1.6; 3.2)	5.28 (3.96; 7.92)	12.3
	HAI_avg_ (N·s)	0.97 (0.93; 0.99)	2.0 (1.5; 2.9)	5.05 (3.84; 7.38)	11.8	0.99 (0.96; 0.99)	1.4 (1.1; 2.1)	3.66 (2.74; 5.48)	8.51	0.98 (0.95; 0.99)	1.6 (1.2; 2.4)	4.16 (3.12; 6.24)	9.68
	HAP_avg_ (W)	0.94 (0.85; 0.97)	6.3 (4.8; 9.3)	65.0 (49.4; 94.9)	151	0.97 (0.92; 0.99)	5.1 (3.8; 7.7)	47.1 (35.3; 70.5)	110	0.98 (0.95; 0.99)	3.5 (2.6; 5.3)	34.1 (25.6; 51.1)	79.4

Definition and description of all outcome variables are presented in Table 1. ICC, intra-class correlation coefficient; CI, confidence interval; CV, coefficient of variation; TE, typical error; MDC, minimal detectable change; *n*^1^, number of participants for overall time and all re-acceleration outcome variables; *n*^2^, number of participants for all phase 1a outcome variables (i.e., overall, initial acceleration and all deceleration phase outcome variables).

For the 105R test (Table 8), when combining all three comparisons, 79.5% (136 out of 171) of all the variables displayed good or better relative reliability, with ICC values ranging from 0.28 to 0.99. Specifically, 48.5% (83 out of 171) of all variables displayed excellent, 31.0% (53 out of 171) displayed good, 18.1% (31 out of 171) displayed moderate, and 2.3% (4 out of 171) displayed poor relative reliability. With regard to the CV values, 97.1% (166 out of 171) of all the variables had acceptable or better absolute reliability, with a range from 0.8 to 13.2%. Specifically, 59.1% (101 out of 171) of all variables displayed good, 38.0% (65 out of 171) displayed acceptable, and 2.9% (5 out of 171) displayed poor absolute reliability. Consequently, and similar to the 105L test, none of the variables displayed very poor absolute reliability. Furthermore, the CV values for the comparison of sessions 1–2 ranged from 1.2 to 11.8 (30/57 good), while the CV values for the comparison of sessions 3–4 ranged from 0.8 to 13.2 (36/57 good).

**Table 8:** Test–retest reliability of overall and phase-specific outcome measurement for the 10-0-5 test with the right foot ultimate step.

Phase	Outcome variables	Test session 1–2 (*n*^1^ = 19; *n*^2^ = 21)				Test session 2–3 (*n*^1^ = 19; *n*^2^ = 20)				Test session 3–4 (*n*^1^ = 19; *n*^2^ = 19)			
		ICC (95% CI)	CV (95% CI)	TE (95% CI)	MDC_90%_	ICC (95% CI)	CV (95% CI)	TE (95% CI)	MDC_90%_	ICC (95% CI)	CV (95% CI)	TE (95% CI)	MDC_90%_
Overall	Time (s)	0.94 (0.85; 0.98)	1.9 (1.4; 2.8)	0.07 (0.05; 0.11)	0.17	0.91 (0.79; 0.97)	2.2 (1.6; 3.2)	0.08 (0.06; 0.12)	0.19	0.95 (0.86; 0.98)	1.5 (1.2; 2.3)	0.06 (0.05; 0.09)	0.14
Phase 1a	Time (s)	0.94 (0.85; 0.97)	1.8 (1.4; 2.6)	0.05 (0.03; 0.07)	0.11	0.90 (0.77; 0.96)	2.3 (1.7; 3.3)	0.06 (0.04; 0.08)	0.13	0.89 (0.73; 0.95)	2.1 (1.6; 3.2)	0.05 (0.04; 0.08)	0.13
	Dist (m)	0.51 (0.11; 0.77)	1.2 (0.9; 1.8)	0.12 (0.09; 0.17)	0.28	0.58 (0.19; 0.81)	1.1 (0.8; 1.6)	0.11 (0.08. 0.15)	0.24	0.75 (0.46; 0.89)	0.8 (0.6; 1.2)	0.08 (0.06; 0.12)	0.18
	V_avg_ (m·s^−1^)	0.94 (0.85. 0.97)	1.7 (1.3; 2.4)	0.06 (0.05; 0.09)	0.15	0.90 (0.77; 0.96)	2.1 (1.6; 3.0)	0.08 (0.06; 0.12)	0.18	0.82 (0.59; 0.93)	2.4 (1.8; 3.6)	0.09 (0.07; 0.14)	0.22
	V_max_ (m·s^−1^)	0.90 (0.77; 0.96)	2.0 (1.5. 2.9)	0.12 (0.09; 0.17)	0.28	0.91 (0.78; 0.96)	2.1 (1.6; 3.0)	0.12 (0.09; 0.18)	0.28	0.95 (0.87; 0.98)	1.4 (1.0; 2.0)	0.08 (0.06; 0.12)	0.20
Initial acceleration	Time (s)	0.94 (0.86; 0.98)	2.9 (2.2; 4.3)	0.04 (0.03; 0.06)	0.10	0.89 (0.75; 0.95)	4.0 (3.0; 5.9)	0.06 (0.05; 0.09)	0.14	0.84 (0.64: 0.94)	4.6 (3.4; 6.9)	0.07 (0.05; 0.10)	0.16
	Dist (m)	0.81 (0.58; 0.92)	3.3 (2.5; 4.8)	0.19 (0.15; 0.28)	0.45	0.82 (0.60; 0.92)	3.7 (2.8; 5.4)	0.21 (0.16; 0.30)	0.48	0.89 (0.74; 0.95)	3.1 (2.4; 4.7)	0.17 (0.13; 0.26)	0.41
Deceleration	Time (s)	0.72 (0.43; 0.88)	4.0 (3.0; 5.8)	0.04 (0.03; 0.06)	0.10	0.71 (0.40; 0.87)	3.9 (3.0; 5.8)	0.04 (0.03; 0.06)	0.10	0.83 (0.61; 0.93)	3.4 (2.5; 5.0)	0.03 (0.03; 0.05)	0.08
	Dist (m)	0.76 (0.49; 0.89)	5.0 (3.8; 7.3)	0.19 (0.14; 0.27)	0.44	0.78 (0.53; 0.91)	5.0 (3.8; 7.4)	0.19 (0.14; 0.28)	0.44	0.79 (0.53; 0.91)	5.1 (3.8; 7.6)	0.20 (0.15; 0.30)	0.47
	Dec_avg_ (m·s^−2^)	0.78 (0.52; 0.90)	5.2 (3.9; 7.6)	0.28 (0.21; 0.40)	0.65	0.85 (0.65; 0.93)	4.4 (3.3; 6.5)	0.24 (0.18; 0.35)	0.55	0.86 (0.67; 0.94)	4.0 (3.0; 6.0)	0.23 (0.17; 0.34)	0.53
	Dec_max_ (m·s^−2^)	0.79 (0.56; 0.91)	6.6 (5.0; 9.7)	0.70 (0.53; 1.00)	1.62	0.67 (0.34; 0.85)	7.0 (5.3; 10.4)	0.79 (0.60; 1.15)	1.83	0.78 (0.51; 0.91)	5.4 (4.1; 8.1)	0.62 (0.47; 0.91)	1.44
	Time to Dec_max_ (s)	0.70 (0.39; 0.86)	6.3 (4.8; 9.2)	0.06 (0.04; 0.08)	0.13	0.68 (0.35; 0.86)	6.0 (4.6; 8.9)	0.06 (0.04; 0.08)	0.13	0.88 (0.73; 0.95)	3.9 (2.9; 5.8)	0.04 (0.03; 0.05)	0.08
	Dist to Dec_max_ (m)	0.49 (0.09. 0.76)	1.2 (0.9; 1.7)	0.12 (0.09; 0.17)	0.27	0.54 (0.14; 0.79)	1.0 (0.8; 1.5)	0.10 (0.08; 0.15)	0.24	0.71 (0.39; 0.88)	0.8 (0.6; 1.1)	0.08 (0.06; 0.11)	0.17
	HBF_avg_ (N)	0.89 (0.76; 0.96)	4.9 (3.7; 7.2)	23.1 (7.66; 33.3)	53.7	0.94 (0.86; 0.98)	4.4 (3.3; 6.4)	19.0 (14.4; 27.7)	44.1	0.94 (0.86; 0.98)	4.2 (3.1; 6.2)	19.3 (14.6; 28.5)	44.9
	HBF_max_ (N)	0.90 (0.77; 0.96)	6.2 (4.7; 9.0)	53.1 (40.6; 76.6)	124	0.85 (0.66; 0.94)	6.9 (5.2; 10.2)	64.5 (49.0; 94.2)	150	0.90 (0.77; 0.96)	5.5 (4.1; 8.2)	53.2 (40.2; 78.7)	124
	HBI_avg_ (N·s)	0.98 (0.95; 0.99)	1.8 (1.4; 2.6)	9.29 (7.11; 13.4)	21.6	0.98 (0.94. 0.99)	2.1 (1.6; 3.1)	9.97 (7.58; 14.6)	23.2	0.99 (0.97; 1.00)	1.4 (1.1; 2.1)	6.90 (5.21; 10.2)	16.0
	HBP_avg_ (W)	0.88 (0.74; 0.95)	6.5 (4.9. 9.5)	92.2 (70.5; 133)	215	0.94 (0.85; 0.97)	5.7 (4.3; 8.4)	74.0 (56.2; 108)	172	0.94 (0.86; 0.98)	4.9 (3.7; 7.4)	70.0 (52.9; 104)	163
	HBP_max_ (W)	0.84 (0.64; 0.93)	9.2 (7.0; 13.6)	211 (161.7; 305)	492	0.94 (0.87; 0.98)	6.6 (5.0; 9.8)	123 (93.8; 180)	287	0.94 (0.86; 0.98)	6.3 (4.7; 9.4)	134 (101; 198)	312
Early deceleration	Time (s)	0.64 (0.30; 0.84)	5.8 (4.4; 8.5)	0.04 (0.03; 0.06)	0.10	0.80 (0.56; 0.91)	4.3 (3.3; 6.4)	0.03 (0.03; 0.05)	0.08	0.78 (0.51; 0.91)	5.0 (3.8; 7.5)	0.04 (0.03; 0.06)	0.09
	Dist (m)	0.73 (0.45; 0.88)	5.7 (4.4; 8.4)	0.20 (0.15; 0.28)	0.46	0.80 (0.57; 0.92)	5.1 (3.9; 7.5)	0.18 (0.14; 0.26)	0.42	0.74 (0.45; 0.89)	6.0 (4.5; 9.0)	0.22 (0.17; 0.32)	0.51
	V_avg_ (m·s^−1^)	0.93 (0.83; 0.97)	1.8 (1.4; 2.7)	0.09 (0.07; 0.13)	0.21	0.92 (0.82; 0.97)	2.1 (1.6; 3.0)	0.10 (0.07; 0.14)	0.22	0.94 (0.86; 0.98)	1.6 (1.2; 2.3)	0.08 (0.06; 0.11)	0.18
	Dec_avg_ (m·s^−2^)	0.63 (0.28; 0.83)	7.1 (5.4; 10.4)	0.28 (0.21; 0.40)	0.65	0.83 (0.63; 0.93)	5.4 (4.1; 7.9)	0.20 (0.15; 0.30)	0.47	0.79 (0.52; 0.91)	5.9 (4.4; 8.8)	0.23 (0.17; 0.34)	0.54
	HBF_avg_ (N)	0.79 (0.55; 0.91)	6.8 (5.1; 9.9)	23.7 (18.1; 34.2)	55.1	0.92 (0.81; 0.97)	5.3 (4.0; 7.8)	16.6 (12.6; 24.3)	38.7	0.90 (0.75; 0.96)	5.9 (4.4; 8.8)	18.9 (14.3; 28.0)	44.1
	HBI_avg_ (N·s)	0.98 (0.95; 0.99)	1.7 (1.3; 2.5)	4.49 (3.40; 6.50)	10.4	0.97 (0.94; 0.99)	2.1 (1.6; 3.1)	5.08 (3.86; 7.42)	11.8	0.99 (0.97; 0.99)	1.4 (1.1; 2.1)	3.54 (2.68; 5.24)	8.24
	HBP_avg_ (W)	0.79 (0.55; 0.91)	8.4 (6.4; 12.4)	134 (102.2; 193)	311	0.91 (0.79; 0.96)	6.8 (5.1; 10.1)	96.7 (73.6; 141)	225	0.90 (0.75; 0.96)	6.7 (5.0; 10.1)	101 (76.3; 149)	235
Late deceleration	Time (s)	0.69 (0.38; 0.86)	7.2 (5.4; 10.5)	0.02 (0.02; 0.03)	0.05	0.51 (0.09; 0.77)	8.4 (6.3; 12.5)	0.02 (0.02; 0.03)	0.05	0.63 (0.25; 0.84)	7.7 (5.7; 11.5)	0.02 (0.02; 0.03)	0.05
	Dist (m)	0.62 (0.26; 0.83)	11.8 (8.9; 17.4)	0.04 (0.03; 0.05)	0.09	0.50 (0.09; 0.77)	13.1 (9.8; 19.7)	0.04 (0.03; 0.06)	0.09	0.52 (0.09; 0.78)	13.2 (9.8; 20.1)	0.04 (0.03; 0.06)	0.10
	V_avg_ (m·s^−1^)	0.82 (0.60; 0.92)	3.0 (2.3; 4.4)	0.05 (0.04; 0.07)	0.11	0.79 (0.55; 0.91)	3.6 (2.7; 5.3)	0.05 (0.04; 0.08)	0.12	0.86 (0.67; 0.94)	2.8 (2.1; 4.2)	0.05 (0.03; 0.07)	0.11
	Dec_avg_ (m·s^−2^)	0.78 (0.53; 0.90)	7.1 (5.4; 10.4)	0.69 (0.53; 1.00)	1.61	0.63 (0.27; 0.83)	8.1 (6.1; 12.0)	0.83 (0.63; 1.21)	1.92	0.69 (0.35; 0.87)	7.4 (5.5; 11.1)	0.77 (0.58; 1.14)	1.80
	HBF_avg_ (N)	0.88 (0.72; 0.95)	6.8 (5.2; 10.0)	54.8 (41.9; 79.2)	128	0.81 (0.58; 0.92)	7.8 (5.9; 11.6)	67.9 (51.6; 99.1)	158	0.84 (0.64; 0.94)	7.4 (5.5; 11.1)	66.6 (50.3; 98.5)	155
	HBI_avg_ (N·s)	0.98 (0.94; 0.99)	1.9 (1.5; 2.8)	4.93 (3.77; 7.12)	11.5	0.98 (0.94; 0.99)	2.2 (1.6; 3.2)	5.03 (3.83; 7.35)	11.7	0.99 (0.97; 1.00)	1.5 (1.1; 2.2)	3.60 (2.70; 5.30)	8.39
	HBP_avg_ (W)	0.91 (0.80; 0.96)	7.2 (5.4; 10.5)	83.5 (63.8; 121)	194	0.86 (0.69; 0.94)	8.0 (6.1; 11.9)	104 (79.2; 152)	242	0.88 (0.72; 0.95)	7.2 (5.4; 10.9)	99.8 (75.4; 147.7)	232
Re-acceleration	Time (s)	0.92 (0.80; 0.97)	2.7 (2.1; 4.1)	0.04 (0.03; 0.05)	0.09	0.92 (0.81; 0.97)	2.5 (1.9; 3.8)	0.04 (0.03; 0.05)	0.08	0.92 (0.80; 0.97)	2.6 (2.0; 3.9)	0.04 (0.03; 0.05)	0.08
	Dist (m)	0.44 (-0.01; 0.74)	2.6 (2.0; 3.9)	0.13 (0.09; 0.19)	0.29	0.53 (0.11; 0.78)	2.3 (1.7; 3.4)	0.11 (0.08; 0.16)	0.25	0.75 (0.46; 0.89)	1.6 (1.2; 2.4)	0.08 (0.06; 0.12)	0.18
	V_avg_ (m·s^−1^)	0.93 (0.83; 0.97)	1.9 (1.4; 2.8)	0.06 (0.05; 0.09)	0.15	0.95 (0.87; 0.98)	1.6 (1.2; 2.4)	0.05 (0.04; 0.08)	0.12	0.96 (0.90; 0.98)	1.3 (1.0; 2.0	0.04 (0.03; 0.07)	0.10
	V_max_ (m·s^−1^)	0.93 (0.82; 0.97)	2.3 (1.8; 3.5)	0.12 (0.09; 0.18)	0.28	0.97 (0.93; 0.99)	1.5 (1.2; 2.3)	0.08 (0.06; 0.11)	0.17	0.96 (0.89; 0.98)	1.7 (1.3; 2.5)	0.09 (0.07; 0.13)	0.20
	Acc_avg_ (m·s^−2^)	0.94 (0.86; 0.98)	3.9 (2.9; 5.8)	0.15 (0.11; 0.22)	0.35	0.97 (0.93; 0.99)	3.0 (2.2; 4.5)	0.10 (0.08; 0.15)	0.24	0.96 (0.91; 0.99)	3.1 (2.3; 4.6)	0.11 (0.08; 0.17)	0.26
	Acc_max_ (m·s^−2^)	0.74 (0.45; 0.89)	9.9 (7.4; 14.9)	0.88 (0.67; 1.31)	2.05	0.61 (0.22; 0.83)	11.9 (8.8; 18.0)	1.08 (0.81; 1.59)	2.50	0.82 (0.59; 0.92)	8.0 (6.0; 12.1)	0.74 (0.56; 1.10)	1.72
	HAF_avg_ (N)	0.97 (0.93; 0.99)	3.3 (2.5; 4.9)	11.8 (8.91; 17.4)	27.4	0.98 (0.96; 0.99)	2.7 (2.0; 4.0)	8.79 (6.64; 13.0)	20.5	0.98 (0.94; 0.99)	2.8 (1.1; 4.1)	9.97 (7.53; 14.8)	23.2
	HAF_max_ (N)	0.83 (0.61; 0.93)	9.3 (7.0; 14.1)	70.2 (53.1; 104)	163	0.76 (0.49; 0.90)	11.2 (8.3; 16.9)	85.3 (64.5; 126)	199	0.88 (0.72; 0.95)	7.7 (5.8; 11.6)	62.6 (47.3; 92.6)	146
	HAI_avg_ (N·s)	0.98 (0.96; 0.99)	1.7 (1.3; 2.5)	8.31 (6.28; 12.3)	19.3	0.99 (0.97; 1.00)	1.6 (1.2; 2.3)	7.21 (5.44; 10.7)	16.8	0.98 (0.96; 0.99)	1.6 (1.2; 2.4)	8.11 (6.13; 12.0)	18.9
	HAP_avg_ (W)	0.96 (0.89; 0.98)	5.1 (3.8; 7.6)	53.0 (40.0; 78.3)	123	0.98 (0.96; 0.99)	3.6 (2.7; 5.4)	30.2 (22.8; 44.7)	70.3	0.98 (0.94; 0.99)	3.7 (2.8; 5.5)	35.4 (26.8; 52.4)	82.4
	HAP_max_ (W)	0.92 (0.81; 0.97)	6.2 (4.7; 9.3)	79.4 (60.0; 117)	185	0.94 (0.86; 0.98)	5.3 (4.0; 8.0)	63.2 (47.7; 93.5)	147	0.96 (0.91; 0.99)	4.1 (3.1; 6.2)	45.9 (34.7; 67.8)	107
Early re-acceleration	Time (s)	0.74 (0.44; 0.89)	6.6 (4.9; 9.9)	0.03 (0.02; 0.04)	0.06	0.74 (0.44; 0.89)	7.1 (5.4; 10.7)	0.03 (0.02; 0.04)	0.06	0.51 (0.08; 0.77)	9.5 (7.1; 14.3)	0.03 (0.03; 0.05)	0.08
	Dist (m)	0.65 (0.29; 0.85)	6.4 (4.8; 9.7)	0.04 (0.03; 0.07)	0.10	0.84 (0.64; 0.93)	5.0 (3.8; 7.5)	0.03 (0.03; 0.05)	0.08	0.28 (-0.19; 0.64)	9.7 (7.3; 14.7)	0.06 (0.05. 0.10)	0.15
	V_avg_ (m·s^−1^)	0.84 (0.64; 0.93)	2.9 (2.2; 4.4)	0.04 (0.03; 0.06)	0.10	0.93 (0.82; 0.97)	2.2 (1.7; 3.3)	0.03 (0.02; 0.05)	0.07	0.82 (0.60; 0.93)	3.5 (2.7; 5.3)	0.05 (0.04; 0.08)	0.12
	Acc_avg_ (m·s^−2^)	0.89 (0.74; 0.95)	6.2 (4.6; 9.3)	0.41 (0.31; 0.61)	0.96	0.84 (0.64; 0.94)	7.3 (5.4; 10.9)	0.47 (0.35; 0.69)	1.09	0.75 (0.45; 0.89)	8.4 (6.3; 12.7)	0.57 (0.43. 0.84)	1.32
	HAF_avg_ (N)	0.93 (0.83; 0.97)	5.8 (4.4; 8.7)	33.4 (25.2; 49.3)	77.6	0.91 (0.77; 0.96)	6.7 (5.1; 10.1)	37.7 (28.5; 55.7)	87.7	0.83 (0.60; 0.93)	7.9 (5.9; 11.9)	48.8 (36.9; 72.1)	114
	HAI_avg_ (N·s)	0.98 (0.95; 0.99)	2.0 (1.5; 2.9)	4.51 (3.41; 6.66)	10.5	0.99 (0.97; 0.99)	1.8 (1.4; 2.7)	3.91 (2.96; 5.79)	9.10	0.97 (0.92; 0.99)	2.4 (1.8; 3.5)	5.46 (4.12; 8.07)	12.7
	HAP_avg_ (W)	0.95 (0.87; 0.98)	6.1 (4.6; 9.1)	48.6 (36.7; 71.9)	113	0.93 (0.84; 0.97)	7.2 (5.4; 10.8)	52.1 (39.4; 77.1)	121	0.90 (0.76; 0.96)	7.4 (5.5; 11.1)	61.2 (46.3; 90.5)	142
Late re-acceleration	Time (s)	0.90 (0.76; 0.96)	3.5 (2.6; 5.2)	0.03 (0.03; 0.05)	0.08	0.96 (0.90; 0.98)	2.0 (1.5; 3.0)	0.02 (0.02; 0.03)	0.05	0.96 (0.89; 0.98)	2.1 (1.6; 3.1)	0.02 (0.02; 0.03)	0.05
	Dist (m)	0.50 (0.06; 0.77)	2.8 (2.1; 4.1)	0.11 (0.08; 0.17)	0.26	0.64 (0.27; 0.84)	2.2 (1.7; 3.3)	0.09 (0.07; 0.14)	0.21	0.86 (0.67; 0.94)	1.4 (1.0; 2.0)	0.06 (0.04; 0.09)	0.13
	V_avg_ (m·s^−1^)	0.91 (0.79; 0.96)	2.3 (1.7; 3.4)	0.10 (0.07; 0.14)	0.22	0.96 (0.89; 0.98)	1.7 (1.3; 2.5)	0.07 (0.05; 0.10)	0.15	0.96 (0.91; 0.99)	1.3 (1.0; 2.0)	0.05 (0.04. 0.08)	0.13
	Acc_avg_ (m·s^−2^)	0.92 (0.81; 0.97)	5.0 (3.8; 7.5)	0.14 (0.10; 0.20	0.32	0.98 (0.95; 0.99)	2.6 (2.0; 3.9)	0.06 (0.05; 0.09)	0.15	0.96 (0.89; 0.98)	3.7 (2.8; 5.4)	0.09 (0.07; 0.13)	0.21
	HAF_avg_ (N)	0.95 (0.89; 0.98)	4.0 (3.0; 6.0)	11.2 (8.49; 16.6)	26.1	0.99 (0.97; 1.00)	2.3 (1.7; 3.4)	5.61 (4.24; 8.29)	13.0	0.97 (0.94. 0.99)	2.9 (2.2; 4.3)	7.57 (5.72; 11.2)	17.6
	HAI_avg_ (N·s)	0.98 (0.96; 0.99)	1.5 (1.2; 2.3)	4.04 (3.05; 5.97)	9.39	0.99 (0.97; 0.99)	1.4 (1.1; 2.1)	3.51 (2.65; 5.19)	8.16	0.99 (0.98; 1.00)	1.1 (0.8; 1.6)	2.94 (2.22; 4.34)	6.83
	HAP_avg_ (W)	0.94 (0.84; 0.97)	6.2 (4.6; 9.3)	71.7 (54.2; 106)	167	0.98 (0.96; 0.99)	3.6 (2.7; 5.3)	34.7 (26.3; 51.4)	80.8	0.97 (0.92; 0.99)	4.4 (3.3; 6.6)	45.6 (34.5; 67.5)	106

Definition and description of all outcome variables are presented in Table 1. ICC, intra-class correlation coefficient; CI, confidence interval; CV, coefficient of variation; TE, typical error; MDC, minimal detectable change; *n*^1^, number of participants for overall time and all re-acceleration outcome variables; *n*^2^, number of participants for all phase 1a outcome variables (i.e., overall, initial acceleration and all deceleration phase outcome variables).

### 3.3. 155 test

For the 155L test (Table 9), when combining all three comparisons, 84.2% (144 out of 171) of all the variables displayed good or better relative reliability, with ICC values ranging from 0.33 to 0.995. Specifically, 54.4% (93 out of 171) of all variables displayed excellent, 29.8% (51 out of 171) displayed good, 8.8% (15 out of 171) displayed moderate, and 7.0% (12 out of 171) displayed poor relative reliability. With regard to the CV values, 96.5% (165 out of 171) of all the variables had acceptable or better absolute reliability, with a range from 0.5 to 16.9%. Specifically, 70.8% (121 out of 171) of all variables displayed good, 25.7% (44 out of 171) displayed acceptable, 2.9% (5 out of 171) displayed poor, and 0.6% (1 out of 171) displayed very poor absolute reliability. Furthermore, the CV values for the comparison of sessions 1–2 ranged from 0.8 to 16.9 (39/57 good), while the CV values for the comparison of sessions 3–4 ranged from 0.5 to 8.1 (41/57 good).

**Table 9:** Test–retest reliability of overall and phase-specific outcome measurement for the 15-0-5 test with the left foot ultimate step.

Phase	Outcome variables	Test session 1 to 2 (*n*^1^ = 18; *n*^2^ = 21)				Test session 2-3 (*n*^1^ = 18; *n*^2^ = 20)				Test session 3-4 (*n*^1^ = 17; *n*^2^ = 19)			
		ICC (95% CI)	CV (95% CI)	TE (95% CI)	MDC_90%_	ICC (95% CI)	CV (95% CI)	TE (95% CI)	MDC_90%_	ICC (95% CI)	CV (95% CI)	TE (95% CI)	MDC_90%_
Overall	Time (s)	0.93 (0.82; 0.97)	1.9 (1.4; 2.9)	0.09 (0.06; 0.13)	0.20	0.95 (0.87; 0.98)	1.5 (1.2; 2.3)	0.07 (0.06; 0.11)	0.17	0.96 (0.90; 0.99)	1.3 (0.9; 1.9)	0.06 (0.04; 0.09)	0.14
Phase 1a	Time (s)	0.88 (0.73; 0.95)	2.4 (1.8; 3.5)	0.08 (0.06; 0.11)	0.18	0.94 (0.85; 0.98)	1.6 (1.2; 2.3)	0.06 (0.04; 0.08)	0.13	0.93 (0.82; 0.97)	1.7 (1.3; 2.5)	0.06 (0.04; 0.08)	0.13
	Dist (m)	0.35 (-0.08; 0.68)	0.8 (0.6; 1.1)	0.11 (0.09; 0.16)	0.26	0.48 (0.06. 0.75)	0.6 (0.4; 0.8)	0.08 (0.06; 0.12)	0.19	0.64 (0.27; 0.84)	0.5 (0.4; 0.8)	0.08 (0.06. 0.11)	0.18
	V_avg_ (m·s^−1^)	0.87 (0.72; 0.95)	2.3 (1.7; 3.3)	0.10 (0.08; 0.15)	0.24	0.94 (0.85; 0.97)	1.6 (1.2; 2.4)	0.07 (0.05; 0.10)	0.16	0.91 (0.78; 0.96)	1.7 (1.2; 2.5)	0.07 (0.06. 0.11)	0.17
	V_max_ (m·s^−1^)	0.97 (0.93; 0.99)	1.2 (0.9; 1.8)	0.08 (0.06; 0.12)	0.20	0.93 (0.84; 0.97)	2.0 (1.5; 2.9)	0.13 (0.10; 0.19)	0.30	0.98 (0.95; 0.99)	1.0 (0.7; 1.4)	0.07 (0.05; 0.10)	0.15
Initial acceleration	Time (s)	0.90 (0.77; 0.96)	3.2 (2.5; 4.7)	0.06 (0.05; 0.09)	0.14	0.93 (0.84; 0.97)	2.6 (2.0; 3.8)	0.05 (0.04; 0.08)	0.13	0.90 (0.76; 0.96)	3.1 (2.4; 4.7)	0.06 (0.05; 0.09)	0.15
	Dist (m)	0.77 (0.51; 0.90)	2.8 (2.1; 4.1)	0.25 (0.19; 0.36)	0.57	0.84 (0.64; 0.93)	2.4 (1.8; 3.5)	0.21 (0.16; 0.30)	0.48	0.86 (0.68; 0.94)	2.3 (1.7; 3.4)	0.20 (0.15; 0.30)	0.46
Deceleration	Time (s)	0.65 (0.31; 0.84)	4.4 (3.4; 6.5)	0.06 (0.04; 0.08)	0.13	0.83 (0.61; 0.93)	3.2 (2.4; 4.7)	0.04 (0.03; 0.06)	0.10	0.86 (0.68; 0.94)	3.0 (2.2; 4.4)	0.04 (0.03; 0.06)	0.09
	Dist (m)	0.78 (0.54; 0.91)	3.7 (2.8; 5.4)	0.21 (0.16; 0.31)	0.49	0.79 (0.55; 0.91)	3.8 (2.9; 5.5)	0.22 (0.16; 0.32)	0.50	0.85 (0.66; 0.94)	3.4 (2.5; 5.1)	0.19 (0.14; 0.28)	0.44
	Dec_avg_ (m·s^−2^)	0.85 (0.67; 0.94)	4.9 (3.7; 7.2)	0.25 (0.19; 0.36)	0.58	0.86 (0.68; 0.94)	4.7 (3.5; 6.9)	0.24 (0.18; 0.35)	0.55	0.91 (0.79; 0.96)	3.5 (2.6; 5.2)	0.19 (0.14; 0.28)	0.44
	Dec_max_ (m·s^−2^)	0.62 (0.26; 0.82)	8.5 (6.5; 12.6)	0.85 (0.65; 1.23)	1.99	0.85 (0.66; 0.94)	4.9 (3.7; 7.3)	0.56 (0.43; 0.82)	1.30	0.78 (0.52; 0.91)	6.7 (5.0; 10.0)	0.73 (0.55; 1.08)	1.70
	Time to Dec_max_ (s)	0.59 (0.22; 0.81)	7.9 (6.0; 11.5)	0.09 (0.07; 0.13)	0.21	0.81 (0.58; 0.92)	4.6 (3.5; 6.9)	0.06 (0.04; 0.08)	0.13	0.81 (0.57; 0.92)	4.3 (3.2; 6.4)	0.05 (0.04; 0.08)	0.12
	Dist to Dec_max_ (m)	0.33 (-0.11; 0.66)	0.7 (0.5; 1.0)	0.10 (0.08; 0.15)	0.24	0.48 (0.05. 0.75)	0.6 (0.4; 0.8)	0.08 (0.06; 0.12)	0.19	0.63 (0.26; 0.84)	0.5 (0.4; 0.7)	0.07 (0.05; 0.10)	0.16
	HBF_avg_ (N)	0.94 (0.86; 0.98)	4.7 (3.6; 6.8)	19.4 (14.9; 28.1)	45.2	0.94 (0.86; 0.98)	4.7 (3.6; 6.9)	19.4 (14.7; 28.3)	45.0	0.96 (0.91; 0.99)	3.4 (2.5; 5.0)	15.4 (11.6; 22.7)	35.7
	HBF_max_ (N)	0.79 (0.56; 0.91)	7.9 (6.0; 11.7)	67.3 (51.5; 97.2)	157	0.92 (0.82; 0.97)	4.8 (3.7; 7.2)	47.6 (36.2; 69.5)	111	0.91 (0.78; 0.96)	6.5 (4.9; 9.8)	58.2 (44.0; 86.1)	136
	HBI_avg_ (N·s)	0.99 (0.99; 1.00)	1.1 (0.8; 1.5)	5.87 (4.49; 8.47)	13.7	0.98 (0.95; 0.99)	2.1 (1.6; 3.1)	11.0 (8.34; 16.0)	25.5	1.00 (0.99; 1.00)	0.9 (0.7; 1.4)	5.15 (3.89; 7.62)	12.0
	HBP_avg_ (W)	0.95 (0.88; 0.98)	5.4 (4.1; 8.0)	78.7 (60.2; 114)	183	0.93 (0.83; 0.97)	6.4 (4.9; 9.5)	89.6 (68.1; 131)	208	0.96 (0.91; 0.99)	4.1 (3.1; 6.1)	64.2 (48.5; 94.9)	149
	HBP_max_ (W)	0.77 (0.52; 0.90)	11.3 (8.6; 16.8)	255 (195; 368)	593	0.92 (0.81; 0.97)	8.0 (6.0; 11.8)	147 (112.1; 215)	343	0.97 (0.93; 0.99)	4.6 (3.4; 6.8)	85.4 (64.5; 126)	199
Early deceleration	Time (s)	0.82 (0.61; 0.92)	4.0 (3.1; 5.9)	0.04 (0.03; 0.06)	0.09	0.74 (0.46; 0.89)	4.9 (3.7; 7.2)	0.05 (0.03; 0.07)	0.11	0.83 (0.62; 0.93)	4.1 (3.1; 6.1)	0.04 (0.03; 0.06)	0.09
	Dist (m)	0.81 (0.59; 0.92)	3.7 (2.8; 5.3)	0.19 (0.14; 0.27)	0.44	0.73 (0.43; 0.88)	4.5 (3.4; 6.7)	0.24 (0.18; 0.35)	0.55	0.84 (0.63; 0.93)	3.8 (2.9; 5.7)	0.20 (0.15; 0.29)	0.45
	V_avg_ (m·s^−1^)	0.95 (0.89; 0.98)	1.5 (1.2; 2.2)	0.08 (0.06; 0.12)	0.19	0.94 (0.86; 0.98)	1.8 (1.3; 2.6)	0.09 (0.07; 0.13)	0.21	0.97 (0.92; 0.99)	1.1 (0.8; 1.7)	0.06 (0.05; 0.09)	0.15
	Dec_avg_ (m·s^−2^)	0.90 (0.77; 0.96)	4.8 (3.6; 6.9)	0.17 (0.13; 0.24)	0.39	0.77 (0.51; 0.90)	6.5 (4.9; 9.6)	0.23 (0.17; 0.33)	0.53	0.87 (0.70; 0.95)	4.7 (3.6; 7.1)	0.17 (0.13; 0.25)	0.39
	HBF_avg_ (N)	0.95 (0.88; 0.98)	4.6 (3.5; 6.7)	13.0 (9.90; 18.7)	30.2	0.89 (0.74; 0.95)	6.4 (4.8; 9.5)	18.4 (14.0; 26.9)	42.9	0.93 (0.83; 0.97)	4.6 (3.4. 6.8)	13.8 (10.4; 20.4)	32.1
	HBI_avg_ (N·s)	0.99 (0.98; 1.00)	1.1 (0.8; 1.6)	3.02 (2.31; 4.36)	7.03	0.98 (0.95. 0.99)	2.0 (1.5; 3.0)	5.24 (3.99; 7.65)	12.2	1.00 (0.99; 1.00)	0.9 (0.7; 1.4)	2.52 (1.91; 3.73)	5.87
	HBP_avg_ (W)	0.95 (0.89; 0.98)	5.4 (4.1; 7.9)	80.6 (61.6; 116)	187	0.89 (0.74; 0.95)	8.0 (6.1; 12.0)	119 (90.7; 174)	277	0.94 (0.85; 0.98)	5.3 (4.0; 7.9)	82.7 (62.5; 122)	193
Late deceleration	Time (s)	0.37 (-0.07; 0.68)	10.5 (7.9; 15.5)	0.04 (0.03; 0.05)	0.09	0.85 (0.66; 0.94)	5.2 (3.9; 7.7)	0.02 (0.01; 0.03)	0.04	0.81 (0.57; 0.92)	6.2 (4.7; 9.4)	0.02 (0.02; 0.03)	0.05
	Dist (m)	0.42 (-0.01; 0.71)	16.9 (12.7; 25.3)	0.09 (0.07; 0.13)	0.20	0.78 (0.52; 0.90)	9.8 (7.4; 14.7)	0.05 (0.04; 0.08)	0.12	0.84 (0.63; 0.93)	8.9 (6.6; 13.4)	0.04 (0.03; 0.06)	0.10
	V_avg_ (m·s^−1^)	0.82 (0.61; 0.92)	3.7 (2.8; 5.4)	0.07 (0.05; 0.10)	0.16	0.83 (0.62; 0.93)	3.6 (2.7; 5.3)	0.07 (0.05; 0.10)	0.15	0.91 (0.79; 0.97)	2.3 (1.8; 3.5)	0.04 (0.03; 0.06)	0.10
	Dec_avg_ (m·s^−2^)	0.49 (0.09; 0.76)	10.6 (8.0; 15.6)	0.93 (0.71; 1.34)	2.16	0.88 (0.72; 0.95)	5.0 (3.8; 7.4)	0.49 (0.37; 0.71)	1.13	0.85 (0.66; 0.94)	6.1 (4.6; 9.1)	0.59 (0.44; 0.87)	1.37
	HBF_avg_ (N)	0.74 (0.46; 0.88)	10.2 (7.7; 15.0)	76.6 (58.6; 111)	178	0.94 (0.86; 0.98)	5.0 (3.8; 7.4)	41.3 (31.4; 60.3)	96.0	0.94 (0.80; 0.98)	5.8 (4.4; 8.8)	45.8 (34.6; 67.7)	107
	HBI_avg_ (N·s)	0.99 (0.99; 1.00)	1.1 (0.8; 1.6)	3.03 (2.32; 4.38)	7.06	0.98 (0.95; 0.99)	2.2 (1.7; 3.3)	5.81 (4.42; 8.49)	13.5	0.99 (0.99; 1.00)	1.0 (0.8; 1.5)	2.80 (2.12; 4.15)	6.52
	HBP_avg_ (W)	0.81 (0.59; 0.92)	10.3 (7.8; 15.3)	135 (103; 195)	315	0.95 (0.88; 0.98)	5.6 (4.2; 8.2)	76.4 (58.1; 112)	178	0.95 (0.88; 0.98)	5.9 (4.5; 8.9)	81.3 (61.5; 120)	189
Re-acceleration	Time (s)	0.92 (0.80; 0.97)	2.3 (1.8; 3.5)	0.03 (0.02; 0.05)	0.07	0.93 (0.83; 0.97)	2.3 (1.7; 3.5)	0.03 (0.02; 0.05)	0.07	0.96 (0.90; 0.99)	1.7 (1.3; 2.7)	0.02 (0.02; 0.03)	0.05
	Dist (m)	0.38 (-0.11; 0.70)	2.4 (1.8; 3.6)	0.11 (0.09; 0.17)	0.26	0.48 (0.02; 0.76)	1.8 (1.3; 2.7)	0.08 (0.06; 0.13)	0.19	0.64 (0.23; 0.84)	1.6 (1.2; 2.5)	0.08 (0.06; 0.12)	0.18
	V_avg_ (m·s^−1^)	0.94 (0.84; 0.97)	1.7 (1.2; 2.5)	0.06 (0.04; 0.08)	0.13	0.97 (0.92; 0.99)	1.3 (1.0; 1.9)	0.04 (0.03; 0.06)	0.10	0.96 (0.90; 0.99)	1.3 (0.9; 1.9)	0.04 (0.03; 0.06)	0.10
	V_max_ (m·s^−1^)	0.95 (0.88; 0.98)	1.7 (1.3; 2.6)	0.09 (0.07; 0.13)	0.20	0.96 (0.90; 0.99)	1.8 (1.4; 2.8)	0.09 (0.06; 0.13)	0.20	0.97 (0.92; 0.99)	1.5 (1.1; 2.2)	0.07 (0.05. 0.11)	0.17
	Acc_avg_ (m·s^−2^)	0.96 (0.90; 0.98)	2.9 (2.2; 4.4)	0.11 (0.09; 0.17)	0.27	0.97 (0.92; 0.99)	3.0 (2.3; 4.6)	0.11 (0.08; 0.16)	0.24	0.98 (0.94; 0.99)	2.4 (1.8; 3.6)	0.09 (0.07; 0.14)	0.21
	Acc_max_ (m·s^−2^)	0.79 (0.52; 0.91)	7.5 (5.6; 11.4)	0.71 (0.53; 1.06)	1.65	0.83 (0.61; 0.93)	6.6 (4.9; 10.4)	0.66 (0.49; 0.99)	1.53	0.76 (0.46; 0.90)	8.1 (5.9; 12.5)	0.81 (0.61; 1.24)	1.89
	HAF_avg_ (N)	0.98 (0.94; 0.99)	2.5 (1.9; 3.8)	9.15 (6.87; 13.7)	21.3	0.98 (0.96; 0.99)	2.7 (2.0; 4.1)	8.61 (6.46; 12.9)	20.0	0.99 (0.96; 0.99)	2.1 (1.5; 3.2)	7.61 (5.67; 11.6)	17.7
	HAF_max_ (N)	0.84 (0.62; 0.93)	7.2 (5.3; 10.9)	57.4 (43.1; 86.1)	134	0.89 (0.74; 0.96)	6.3 (4.7; 9.5)	54.7 (41.1; 82.1)	127	0.88 (0.69; 0.95)	7.6 (5.6; 11.8)	63.0 (46.9; 95.9)	147
	HAI_avg_ (N·s)	0.99 (0.97; 1.00)	1.4 (1.1; 2.1)	6.6 (4.94; 9.87)	15.3	0.99 (0.96; 0.99)	1.8 (1.4; 2.8)	7.74 (5.81; 11.6)	18.0	0.99 (0.97; 1.00)	1.4 (1.1; 2.2)	6.61 (4.93; 10.1)	15.4
	HAP_avg_ (W)	0.97 (0.93; 0.99)	3.7 (2.7; 5.5)	37.1 (27.9; 55.6)	86.3	0.98 (0.96; 0.99)	3.8 (2.9; 5.8)	30.3 (22.7; 45.4)	70.5	0.98 (0.95; 0.99)	3.0 (2.3; 4.7)	29.8 (22.2; 45.4)	69.4
	HAP_max_ (W)	0.96 (0.89; 0.98)	4.6 (3.5; 7.1)	57.0 (42.8; 85.5)	133	0.94 (0.86; 0.98)	5.8 (4.4; 8.9)	68.3 (51.3; 102)	159	0.92 (0.79; 0.97)	6.7 (4.9; 10.3)	82.0 (61.1; 125)	191
Early re-acceleration	Time (s)	0.62 (0.23; 0.83)	8.2 (6.1; 12.5)	0.03 (0.02; 0.04)	0.07	0.53 (0.09; 0.79)	8.8 (6.5; 13.4)	0.03 (0.03; 0.05)	0.08	0.55 (0.10; 0.80)	7.5 (5.6; 11.7)	0.03 (0.02; 0.04)	0.06
	Dist (m)	0.44 (-0.04; 0.74)	9.5 (7.0; 14.5)	0.06 (0.04; 0.09)	0.14	0.45 (-0.01; 0.75)	9.1 (6.7; 13.9)	0.06 (0.04; 0.08)	0.13	0.57 (0.13; 0.81)	7.5 (5.6; 11.7)	0.05 (0.04; 0.07)	0.11
	V_avg_ (m·s^−1^)	0.76 (0.47; 0.90)	4.1 (3.1; 6.3)	0.06 (0.04; 0.09)	0.14	0.87 (0.70; 0.95)	3.4 (2.5; 5.1)	0.05 (0.04; 0.07)	0.11	0.90 (0.76; 0.96)	2.9 (2.2; 4.5)	0.04 (0.03; 0.07)	0.10
	Acc_avg_ (m·s^−2^)	0.80 (0.55; 0.92)	7.7 (5.7; 11.7)	0.50 (0.38; 0.75)	1.16	0.83 (0.60; 0.93)	7.9 (5.9; 12.1)	0.49 (0.37; 0.73)	1.14	0.77 (0.47; 0.90)	7.3 (5.4; 11.4)	0.50 (0.37; 0.76)	1.17
	HAF_avg_ (N)	0.85 (0.65; 0.94)	6.9 (5.1; 10.5)	39.6 (29.7; 59.4)	92.2	0.89 (0.74; 0.96)	7.2 (5.3; 10.9)	38.3 (28.7; 57.4)	89.1	0.86 (0.67; 0.95)	6.7 (5.0; 10.4)	40.3 (30.0; 61.3)	93.7
	HAI_avg_ (N·s)	0.98 (0.95; 0.99)	2.0 (1.5; 3.1)	4.26 (3.20; 6.39)	9.91	0.98 (0.94; 0.99)	2.6 (1.9; 3.9)	4.84 (3.63; 7.26)	11.3	0.99 (0.96; 0.99)	1.8 (1.4; 2.8)	3.86 (2.87; 5.87)	8.97
	HAP_avg_ (W)	0.92 (0.80; 0.97)	6.6 (4.9; 10.1)	51.0 (38.3; 76.5)	119	0.94 (0.85. 0.98)	7.0 (5.2; 10.7)	49.8 (37.3; 74.6)	116	0.92 (0.79; 0.97)	6.7 (4.9; 10.3)	54.5 (40.6; 82.9)	127
Late re-acceleration	Time (s)	0.88 (0.72; 0.95)	3.3 (2.4; 4.9)	0.03 (0.02; 0.05)	0.07	0.92 (0.79; 0.97)	2.8 (2.1; 4.2)	0.03 (0.02; 0.04)	0.07	0.95 (0.87; 0.98)	2.3 (1.7; 3.5)	0.02 (0.02; 0.04)	0.05
	Dist (m)	0.34 (-0.16; 0.68)	2.9 (2.2; 4.4)	0.12 (0.09; 0.18)	0.27	0.59 (0.18; 0.82)	2.0 (1.5; 3.1)	0.08 (0.06; 0.13)	0.20	0.61 (0.20; 0.83)	2.3 (1.7; 3.5)	0.09 (0.07; 0.14)	0.21
	V_avg_ (m·s^−1^)	0.96 (0.90; 0.98)	1.4 (1.1; 2.1)	0.06 (0.04; 0.09)	0.13	0.98 (0.95; 0.99)	1.1 (0.8; 1.7)	0.04 (0.03; 0.06)	0.10	0.98 (0.94; 0.99)	1.0 (0.8; 1.6)	0.04 (0.03; 0.06)	0.10
	Acc_avg_ (m·s^−2^)	0.93 (0.84; 0.97)	4.2 (3.1; 6.4)	0.11 (0.08; 0.16)	0.25	0.96 (0.90; 0.98)	4.2 (3.1; 6.3	0.09 (0.07; 0.13)	0.21	0.97 (0.92; 0.99)	3.1 (2.3; 4.8)	0.08 (0.06; 0.12)	0.18
	HAF_avg_ (N)	0.97 (0.92; 0.99)	3.3 (2.5; 5.0)	8.63 (6.48; 12.9)	20.1	0.98 (0.95; 0.99)	3.2 (2.4; 4.9)	7.15 (5.36; 10.7)	16.6	0.98 (0.95; 0.99)	2.4 (1.8; 3.7)	6.43 (4.79; 9.78)	15.0
	HAI_avg_ (N·s)	0.99 (0.97; 1.00)	1.2 (0.9; 1.8)	3.04 (2.28; 4.56)	7.07	0.99 (0.97; 1.00)	1.4 (1.1; 2.2)	3.39 (2.54; 5.08)	7.88	0.99 (0.96; 0.99)	1.3 (1.0; 2.0)	3.33 (2.48; 5.07)	7.74
	HAP_avg_ (W)	0.96 (0.91; 0.99)	4.6 (3.5; 7.0)	47.1 (35.4; 70.6)	110	0.98 (0.95; 0.99)	4.7 (3.5; 7.1)	37.0 (27.8; 55.5)	86.1	0.98 (0.94; 0.99)	3.5 (2.6; 5.4)	36.1 (26.9; 54.9)	83.9

Definition and description of all outcome variables are presented in Table 1. ICC, intra-class correlation coefficient; CI, confidence interval; CV, coefficient of variation; TE, typical error; MDC, minimal detectable change; *n*^1^, number of participants for overall time and all re-acceleration outcome variables; *n*^2^, number of participants for all phase 1a outcome variables (i.e., overall, initial acceleration and all deceleration phase outcome variables).

For the 155R test (Table 10), when combining all three comparisons, 75.4% (129 out of 171) of all the variables displayed good or better relative reliability, with ICC values ranging from 0.13 to 0.99. Specifically, 53.2% (91 out of 171) of all variables displayed excellent, 22.2% (38 out of 171) displayed good, 13.5% (23 out of 171) displayed moderate, and 11.1% (19 out of 171) displayed poor relative reliability. With regard to the CV values, 95.3% (163 out of 171) of all the variables had acceptable or better absolute reliability, with a range from 0.6 to 14.0%. Specifically, 57.9% (99 out of 171) of all variables displayed good, 37.4% (64 out of 171) displayed acceptable, and 4.7% (8 out of 171) displayed poor absolute reliability. None of the variables displayed very poor absolute reliability (i.e., a CV value of ≥15%). Furthermore, the CV values for the comparison of sessions 1–2 ranged from 0.7 to 12.9 (29/57 good), while the CV values for the comparison of sessions 3–4 ranged from 0.6 to 13.4 (42/57 good).

**Table 10:** Test–retest reliability of overall and phase-specific outcome measurement for the 15-0-5 test with the right foot ultimate step.

Phase	Outcome variables	Test session 1 to 2 (*n*^1^ = 19; *n*^2^ = 21)				Test session 2-3 (*n*^1^ = 19; *n*^2^ = 20)				Test session 3-4 (*n*^1^ = 19; *n*^2^ = 19)			
		ICC (95% CI)	CV (95% CI)	TE (95% CI)	MDC_90%_	ICC (95% CI)	CV (95% CI)	TE (95% CI)	MDC_90%_	ICC (95% CI)	CV (95% CI)	TE (95% CI)	MDC_90%_
Overall	Time (s)	0.94 (0.86; 0.98)	1.7 (1.3; 2.5)	0.08 (0.06; 0.12)	0.19	0.95 (0.88; 0.98)	1.6 (1.2; 2.4)	0.08 (0.06; 0.11)	0.18	0.96 (0.91; 0.99)	1.3 (1.0; 1.9)	0.06 (0.05; 0.09)	0.14
Phase 1a	Time (s)	0.92 (0.80; 0.96)	2.0 (1.5; 2.8)	0.06 (0.05; 0.09)	0.15	0.95 (0.88; 0.98)	1.5 (1.2; 2.3)	0.05 (0.04. 0.08)	0.12	0.92 (0.81; 0.97)	1.7 (1.3; 2.6)	0.06 (0.04; 0.08)	0.13
	Dist (m)	0.69 (0.37; 0.86)	0.7 (0.6; 1.0)	0.11 (0.08; 0.15)	0.25	0.40 (-0.05; 0.70)	0.8 (0.6; 1.1)	0.11 (0.09; 0.17)	0.27	0.65 (0.30; 0.85)	0.6 (0.5; 0.9)	0.09 (0.07; 0.14)	0.22
	V_avg_ (m·s^−1^)	0.91 (0.79; 0.96)	1.8 (1.4; 2.7)	0.08 (0.06; 0.12)	0.19	0.96 (0.89; 0.98)	1.4 (1.0; 2.0)	0.06 (0.04; 0.09)	0.14	0.89 (0.73; 0.95)	1.9 (1.4; 2.8)	0.08 (0.06; 0.12)	0.19
	V_max_ (m·s^−1^)	0.95 (0.88; 0.98)	1.7 (1.3; 2.5)	0.11 (0.09; 0.16)	0.26	0.95 (0.87; 0.98)	1.8 (1.4; 2.6)	0.12 (0.09; 0.17)	0.27	0.96 (0.90; 0.98)	1.4 (1.0; 2.1)	0.09 (0.07; 0.13)	0.21
Initial acceleration	Time (s)	0.79 (0.54; 0.91)	4.7 (3.6; 6.9)	0.09 (0.07; 0.14)	0.22	0.79 (0.55; 0.91)	5.1 (3.9; 7.6)	0.10 (0.07; 0.14)	0.23	0.92 (0.80; 0.97)	3.1 (2.3; 4.6)	0.06 (0.04; 0.09)	0.14
	Dist (m)	0.24 (-0.21; 0.60)	5.6 (4.3; 8.2)	0.49 (0.38; 0.71)	1.15	0.44 (0.01; 0.73)	5.2 (3.9; 7.7)	0.45 (0.34; 0.66)	1.05	0.93 (0.83; 0.97)	2.1 (1.6; 3.2)	0.19 (0.15; 0.29)	0.45
Deceleration	Time (s)	0.18 (-0.26; 0.56)	6.9 (5.3; 10.2)	0.09 (0.07; 0.13)	0.21	0.42 (-0.02; 0.72)	6.0 (4.6; 8.9)	0.08 (0.06; 0.12)	0.19	0.87 (0.69; 0.95)	3.3 (2.5; 5.0)	0.04 (0.03; 0.06)	0.10
	Dist (m)	0.18 (-0.27; 0.56)	8.5 (6.4; 12.5)	0.48 (0.36; 0.69)	1.11	0.43 (0.00; 0.73)	7.5 (5.7; 11.2)	0.42 (0.32; 0.62)	0.98	0.94 (0.86; 0.98)	2.6 (2.0. 3.9)	0.14 (0.11; 0.21)	0.33
	Dec_avg_ (m·s^−2^)	0.59 (0.23; 0.81)	8.1 (6.2; 11.9)	0.39 (0.30. 0.56)	0.91	0.78 (0.52; 0.90)	6.5 (4.9; 9.7)	0.31 (0.24; 0.45)	0.72	0.90 (0.76; 0.96)	4.1 (3.1; 6.1)	0.22 (0.16; 0.32)	0.51
	Dec_max_ (m·s^−2^)	0.84 (0.65; 0.93)	5.4 (4.1; 7.8)	0.59 (0.45; 0.85)	1.37	0.88 (0.73; 0.95)	4.3 (3.3; 6.4)	0.49 (0.37; 0.71)	1.13	0.82 (0.60; 0.93)	5.1 (3.8; 7.6)	0.58 (0.44; 0.86)	1.35
	Time to Dec_max_ (s)	0.20 (-0.24; 0.57)	9.8 (7.4; 14.4)	0.12 (0.09; 0.17)	0.27	0.36 (-0.09; 0.68)	8.7 (6.5; 12.9)	0.10 (0.08; 0.15)	0.24	0.85 (0.64; 0.94)	5.3 (4.0; 8.0)	0.06 (0.04; 0.09)	0.14
	Dist to Dec_max_ (m)	0.64 (0.29; 0.83)	0.7 (0.6; 1.1)	0.11 (0.08; 0.16)	0.25	0.38 (-0.07; 0.69)	0.7 (0.6; 1.1)	0.11 (0.08; 0.16)	0.25	0.50 (0.07; 0.77)	0.7 (0.5; 1.0)	0.10 (0.07; 0.15)	0.23
	HBF_avg_ (N)	0.80 (0.57; 0.91)	7.8 (5.9; 11.5)	32.6 (24.9; 47.0)	75.8	0.91 (0.78; 0.96)	6.2 (4.7. 9.1)	24.4 (18.6; 35.7)	56.8	0.96 (0.90; 0.98)	3.8 (2.8; 5.6)	17.2 (13.0; 25.4)	40.0
	HBF_max_ (N)	0.92 (0.81; 0.97)	5.1 (3.9; 7.5)	47.9 (36.6; 69.1)	111	0.95 (0.87; 0.98)	4.5 (3.4; 6.7)	39.8 (30.3; 58.2)	92.7	0.93 (0.82; 0.97)	4.9 (3.7; 7.4)	46.4 (35.0; 68.6)	108
	HBI_avg_ (N·s)	0.99 (0.97; 0.99)	1.6 (1.3; 2.4)	8.70 (6.70; 12.6)	20.3	0.99 (0.97; 0.99)	1.8 (1.4; 2.6)	9.00 (6.84; 13.2)	20.9	0.99 (0.98; 1.00)	1.1 (0.9; 1.7)	5.98 (4.52; 8.84)	13.9
	HBP_avg_ (W)	0.84 (0.64; 0.93)	9.0 (6.8; 13.2)	128 (97.5; 184)	297	0.93 (0.83; 0.97)	6.9 (5.2; 10.3)	90.0 (68.5; 132)	210	0.96 (0.90; 0.98)	4.8 (3.6; 7.2)	72.8 (55.0; 108)	169
	HBP_max_ (W)	0.78 (0.53; 0.90)	10.8 (8.1; 15.9)	250 (191; 361)	581	0.90 (0.76; 0.96)	8.1 (6.1; 12.0)	164 (125; 239)	381	0.85 (0.66. 0.94)	9.3 (6.9; 14.0)	211 (159.0; 311)	490
Early deceleration	Time (s)	0.13 (-0.31; 0.52)	9.0 (6.8; 13.2)	0.09 (0.07; 0.13)	0.20	0.50 (0.09; 0.77)	7.2 (5.4; 10.7)	0.07 (0.05; 0.10)	0.17	0.92 (0.81; 0.97)	3.2 (2.4; 4.7)	0.03 (0.02. 0.04)	0.07
	Dist (m)	0.18 (-0.27; 0.56)	9.1 (6.9; 13.5)	0.47 (0.36; 0.67)	1.08	0.50 (0.08; 0.76)	7.6 (5.8; 11.3)	0.39 (0.30; 0.57)	0.91	0.94 (0.86; 0.98)	2.8 (2.1; 4.1)	0.14 (0.10; 0.20)	0.32
	V_avg_ (m·s^−1^)	0.92 (0.81; 0.97)	2.1 (1.6; 3.1)	0.12 (0.09; 0.17)	0.27	0.93 (0.82; 0.97)	2.0 (1.6; 3.0)	0.11 (0.08; 0.16)	0.25	0.92 (0.80; 0.97)	1.9 (1.5; 2.9)	0.11 (0.08; 0.16)	0.25
	Dec_avg_ (m·s^−2^)	0.52 (0.12; 0.77)	10.3 (7.8; 15.2)	0.32 (0.25; 0.47)	0.75	0.78 (0.52; 0.90)	8.0 (6.0; 11.9)	0.25 (0.19; 0.36)	0.57	0.93 (0.83; 0.97)	4.0 (3.0; 5.9)	0.14 (0.11; 0.21)	0.33
	HBF_avg_ (N)	0.73 (0.45; 0.88)	9.8 (7.4; 14.4)	26.7 (20.4; 38.5)	62.1	0.89 (0.75; 0.96)	7.4 (5.6; 11.0)	19.1 (14.5; 27.9)	44.5	0.97 (0.93; 0.99)	3.4 (2.6; 5.1)	10.4 (7.88; 15.4)	24.3
	HBI_avg_ (N·s)	0.99 (0.98; 1.00)	1.4 (1.0; 2.0)	3.60 (2.80; 5.30)	8.50	0.99 (0.97; 0.99)	1.7 (1.3; 2.4)	4.28 (3.25; 6.25)	9.96	0.99 (0.98. 1.00)	1.2 (0.9; 1.8)	3.23 (2.44; 4.78)	7.52
	HBP_avg_ (W)	0.79 (0.55; 0.91)	11.1 (8.4; 16.4)	154 (118; 222)	357	0.92 (0.81; 0.97)	8.5 (6.4; 12.6)	106 (80.5; 155)	246	0.97 (0.92; 0.99)	4.5 (3.4; 6.7)	67.4 (50.9; 99.6)	157
Late deceleration	Time (s)	0.58 (0.21; 0.81)	7.5 (5.7; 11.0)	0.03 (0.02; 0.04)	0.06	0.60 (0.23; 0.82)	7.4 (5.6; 11.0)	0.03 (0.02; 0.04)	0.06	0.63 (0.26; 0.84)	7.9 (5.9; 11.9)	0.03 (0.02; 0.04)	0.06
	Dist (m)	0.52 (0.13; 0.78)	12.9 (9.7; 19.1)	0.06 (0.05; 0.09)	0.15	0.53 (0.13; 0.78)	14.0 (10.5; 21.1)	0.07 (0.05; 0.10)	0.17	0.60 (0.21; 0.82)	13.4 (10.0; 20.5)	0.07 (0.05; 0.11)	0.17
	V_avg_ (m·s^−1^)	0.81 (0.59; 0.92)	3.6 (2.7; 5.2)	0.06 (0.05; 0.09)	0.15	0.82 (0.60; 0.92)	3.8 (2.9; 5.6)	0.07 (0.05; 0.10)	0.16	0.77 (0.50; 0.90)	4.4 (3.3; 6.6)	0.08 (0.06; 0.11)	0.18
	Dec_avg_ (m·s^−2^)	0.81 (0.59; 0.92)	7.3 (5.5; 10.7)	0.61 (0.46; 0.88)	1.41	0.83 (0.61; 0.93)	6.7 (5.1; 10.0)	0.59 (0.45; 0.86)	1.38	0.71 (0.38; 0.87)	8.3 (6.2; 12.6)	0.81 (0.61; 1.20)	1.89
	HBF_avg_ (N)	0.90 (0.78; 0.96)	6.9 (5.2; 10.1)	45.8 (35.0; 66.1)	107	0.92 (0.80; 0.97)	6.4 (4.8; 9.5)	45.9 (34.9; 67.0)	107	0.85 (0.66; 0.94)	8.1 (6.1; 12.3)	66.9 (50.5; 98.9)	156
	HBI_avg_ (N·s)	0.98 (0.96; 0.99)	2.0 (1.5; 2.9)	5.17 (3.96; 7.47)	12.0	0.99 (0.96; 0.99)	2.0 (1.5; 3.0)	4.87 (3.70; 7.11)	11.3	0.99 (0.98; 1.00)	1.1 (0.9; 1.7)	2.99 (2.26; 4.42)	6.95
	HBP_avg_ (W)	0.93 (0.83; 0.97)	7.0 (5.3; 10.3)	83.8 (64.1; 121)	195	0.94 (0.87; 0.98)	6.1 (4.6; 9.0)	76.9 (58.5; 112)	179	0.87 (0.70; 0.95)	8.7 (6.5; 13.1)	126 (95.1; 186)	293
Re-acceleration	Time (s)	0.92 (0.81; 0.97)	2.5 (1.9; 3.7)	0.04 (0.03; 0.05)	0.08	0.92 (0.80; 0.97)	2.7 (2.0; 4.0)	0.04 (0.03; 0.05)	0.09	0.97 (0.93; 0.99)	1.6 (1.2; 2.4)	0.02 (0.02; 0.03)	0.05
	Dist (m)	0.64 (0.27; 0.84)	2.3 (1.7; 3.4)	0.11 (0.08; 0.16)	0.25	0.38 (-0.08; 0.70)	2.2 (1.7; 3.3)	0.11 (0.08; 0.16)	0.25	0.65 (0.29; 0.85)	1.9 (1.5; 2.9)	0.09 (0.07; 0.14)	0.22
	V_avg_ (m·s^−1^)	0.96 (0.89; 0.98)	1.5 (1.1; 2.2)	0.05 (0.04. 0.07)	0.11	0.97 (0.92; 0.99)	1.3 (1.0; 2.0)	0.04 (0.03; 0.07)	0.10	0.95 (0.88; 0.98)	1.4 (1.1; 2.1)	0.05 (0.04; 0.07)	0.11
	V_max_ (m·s^−1^)	0.96 (0.91; 0.98)	1.8 (1.4; 2.7)	0.09 (0.07; 0.13)	0.20	0.98 (0.95; 0.99)	1.3 (1.0; 1.9)	0.06 (0.05; 0.09)	0.15	0.97 (0.92; 0.99)	1.4 (1.1; 2.1)	0.07 (0.06; 0.11)	0.17
	Acc_avg_ (m·s^−2^)	0.96 (0.91; 0.98)	3.6 (2.7; 5.4)	0.12 (0.09. 0.17)	0.27	0.96 (0.90; 0.98)	3.5 (2.6; 5.2)	0.12 (0.09; 0.18)	0.29	0.98 (0.95; 0.99)	2.3 (1.7; 3.4)	0.09 (0.07; 0.13)	0.20
	Acc_max_ (m·s^−2^)	0.67 (0.32; 0.86)	9.3 (7.0; 14.1)	0.89 (0.68. 1.32)	2.08	0.78 (0.51; 0.91)	7.7 (5.8; 11.7)	0.77 (0.58; 1.14)	1.79	0.75 (0.47; 0.90)	9.2 (6.9; 14.0)	0.83 (0.63; 1.23)	1.93
	HAF_avg_ (N)	0.98 (0.95; 0.99)	3.0 (2.3; 4.5)	9.33 (7.05; 13.8)	21.7	0.98 (0.94; 0.99)	3.1 (2.3; 4.6)	10.6 (8.01; 15.7)	24.7	0.99 (0.97; 1.00)	2.1 (1.6; 3.1)	7.41 (5.60; 11.0)	17.2
	HAF_max_ (N)	0.80 (0.56; 0.92)	8.7 (6.5; 13.2)	71.4 (54.0; 106)	166	0.87 (0.70; 0.95)	7.4 (5.5; 11.1)	60.2 (45.5; 89.0)	140	0.85 (0.65; 0.94)	8.6 (6.4; 12.9)	65.5 (49.5; 96.9)	153
	HAI_avg_ (N·s)	0.99 (0.98; 1.00)	1.3 (1.0; 2.0)	5.80 (4.38; 8.57)	13.5	0.99 (0.99; 1.00)	1.0 (0.7; 1.4)	4.53 (3.42; 6.69)	10.5	0.99 (0.97; 1.00)	1.3 (1.0; 2.0)	6.49 (4.90. 9.59)	15.1
	HAP_avg_ (W)	0.98 (0.95; 0.99)	4.0 (3.0; 6.0)	32.6 (24.6; 48.2)	75.8	0.98 (0.94; 0.99)	3.8 (2.9; 5.7)	35.0 (26.5; 51.8)	81.5	0.98 (0.96; 0.99)	3.0 (2.3; 4.5)	30.3 (22.9; 44.8)	70.5
	HAP_max_ (W)	0.89 (0.74; 0.95)	7.6 (5.7; 11.5)	83.8 (63.4; 124)	195	0.91 (0.78; 0.96)	6.3 (4.7; 9.4)	78.6 (59.4; 116)	183	0.97 (0.92; 0.99)	3.9 (3.0; 5.9)	47.6 (36.0; 70.4)	111
Early re-acceleration	Time (s)	0.48 (0.03; 0.75)	10.1 (7.6; 15.3)	0.04 (0.03; 0.05)	0.08	0.48 (0.04; 0.76)	9.7 (7.2; 14.7)	0.03 (0.03; 0.05)	0.08	0.80 (0.55; 0.92)	5.7 (4.3; 8.6)	0.02 (0.02; 0.03)	0.05
	Dist (m)	0.42 (-0.04; 0.72)	10.6 (7.9; 16.0)	0.06 (0.05; 0.10)	0.15	0.42 (-0.03; 0.73)	8.4 (6.3; 12.7)	0.05 (0.04; 0.08)	0.12	0.58 (0.19; 0.81)	6.5 (4.9; 9.7)	0.04 (0.03; 0.06)	0.10
	V_avg_ (m·s^−1^)	0.77 (0.50; 0.90)	3.8 (2.9; 5.7)	0.05 (0.04; 0.08)	0.13	0.95 (0.88; 0.98)	1.8 (1.4; 2.7)	0.03 (0.02; 0.04)	0.06	0.85 (0.66; 0.94)	3.3 (2.5; 4.9)	0.05 (0.03. 0.07)	0.11
	Acc_avg_ (m·s^−2^)	0.73 (0.42; 0.88)	9.5 (7.1; 14.4)	0.62 (0.47; 0.92)	1.44	0.73 (0.42; 0.88)	9.8 (7.3; 14.9)	0.65 (0.49; 0.96)	1.51	0.89 (0.74; 0.96)	5.5 (4.2; 8.3)	0.39 (0.29; 0.57)	0.90
	HAF_avg_ (N)	0.84 (0.64; 0.93)	8.7 (6.5; 13.1)	47.1 (35.6; 69.6)	110	0.82 (0.59; 0.93)	9.2 (6.9; 13.9)	52.4 (39.6; 77.5)	122	0.93 (0.82; 0.97)	5.1 (3.8; 7.6)	31.3 (23.6; 46.3)	72.8
	HAI_avg_ (N·s)	0.98 (0.96; 0.99)	2.2 (1.7; 3.3)	4.27 (3.22; 6.31)	9.92	0.99 (0.98; 1.00)	1.3 (1.0; 2.0)	2.82 (2.13; 4.18)	6.57	0.98 (0.96; 0.99)	1.7 (1.3; 2.6)	3.83 (2.89. 5.66)	8.90
	HAP_avg_ (W)	0.92 (0.81; 0.97)	8.0 (6.0; 12.0)	55.8 (42.1; 82.5)	130	0.89 (0.73; 0.95)	9.3 (6.9; 14.0)	69.6 (52.6; 103)	162	0.95 (0.87; 0.98)	5.2 (3.9; 7.8)	44.2 (33.4; 65.3)	103
Late re-acceleration	Time (s)	0.87 (0.70; 0.95)	3.5 (2.7; 5.3)	0.04 (0.03; 0.05)	0.09	0.94 (0.86; 0.98)	2.5 (1.9; 3.7)	0.02 (0.02; 0.04)	0.06	0.96 (0.90; 0.98)	2.1 (1.6; 3.2	0.02 (0.02; 0.03)	0.05
	Dist (m)	0.40 (-0.06; 0.71)	2.9 (2.2; 4.3)	0.12 (0.09; 0.18)	0.28	0.49 (0.06; 0.77)	2.2 (1.7; 3.3)	0.09 (0.07; 0.14)	0.22	0.72 (0.40; 0.88)	2.1 (1.6; 3.2)	0.09 (0.07; 0.13)	0.20
	V_avg_ (m·s^−1^)	0.96 (0.90; 0.98)	1.6 (1.2; 2.4)	0.06 (0.05; 0.09)	0.15	0.96 (0.90; 0.98)	1.6 (1.2. 2.3)	0.06 (0.05; 0.09)	0.14	0.95 (0.89; 0.98)	1.4 (1.1; 2.1)	0.06 (0.04; 0.09)	0.14
	Acc_avg_ (m·s^−2^)	0.93 (0.84; 0.97)	5.1 (3.8; 7.6)	0.12 (0.09; 0.17)	0.27	0.96 (0.91; 0.99)	3.5 (2.6; 5.1)	0.08 (0.06; 0.13)	0.20	0.97 (0.92; 0.99)	2.9 (2.2; 4.4)	0.08 (0.06; 0.11)	0.18
	HAF_avg_ (N)	0.97 (0.92; 0.99)	3.8 (2.8; 5.6)	8.73 (6.60; 12.9)	20.3	0.98 (0.95; 0.99)	2.7 (2.0; 4.0)	6.90 (5.20; 10.2)	16.0	0.98 (0.96; 0.99)	2.4 (1.8; 3.6)	6.30 (4.76; 9.31)	14.7
	HAI_avg_ (N·s)	0.99 (0.99; 1.00)	0.9 (0.7; 1.3)	2.21 (1.67; 3.27)	5.15	0.99 (0.98; 1.00)	1.0 (0.7; 1.4)	2.47 (1.86; 3.65)	5.74	0.99 (0.97; 1.00)	1.2 (0.9; 1.7)	3.00 (2.27; 4.43)	6.97
	HAP_avg_ (W)	0.97 (0.92; 0.99)	5.3 (4.0; 8.0)	46.8 (35.4; 69.2)	109	0.98 (0.95; 0.99)	3.8 (2.8; 5.6)	36.6 (27.6; 54.1)	85.1	0.98 (0.95; 0.99)	3.5 (2.6; 5.2)	36.4 (27.5; 53.8)	84.7

Definition and description of all outcome variables are presented in Table 1. ICC, intra-class correlation coefficient; CI, confidence interval; CV, coefficient of variation; TE, typical error; MDC, minimal detectable change; *n*^1^, number of participants for overall time and all re-acceleration outcome variables; *n*^2^, number of participants for all phase 1a outcome variables (i.e., overall, initial acceleration and all deceleration phase outcome variables).

### 3.4. Learning effect

Overall, the results from the RM ANOVAs/Friedman tests exhibited a statistically significant difference (defined as an adjusted *p*-value of <0.10) between the test sessions for 11 variables out of a total of 342 variables, based on the Holm–Bonferroni adjusted *p*-values (results not shown). Out of the 11 statistically significant variables (Table 11), 7 belonged to m505L, 2 to m505R, 1 to 105L, and 1 to 105R. Thus, no statistically significant results were found for any variables from the 155 tests. Test statistics [*F*-value with corresponding degrees of freedom, adjusted *p*-value, and omega-squared effect size (*ω*²) with a 95% CI] from each RM ANOVA are presented in Table 11 in the left column below their given variable name. However, based on MDC_90%_ comparisons, no “real” differences were found between the sessions for the 11 identified variables. Of note, only test statistics from the RM ANOVAs are presented in Table 11 as none of the Friedman tests demonstrated a statistically significant difference.

**Table 11:** *Post hoc* pairwise comparison of variables where a statistically significant result was found from the RM ANOVA/Friedman tests.

			Mean difference (95% CI)	*t*	Holm-B adj. *p*-value	Mean diff > MDC_90%_		Hedges’g_av_ (95% CI)
m505_L__L-Dec_Phase_HBP_avg_	S1	S2	−45.6 (−93.4; 2.2)	−2.83	0.045*	140	No	−0.21 (−0.38; −0.07)
(*F*_(2.98, 53.75)_ = 11.14,	* *	S3	−90.9 (−156; −26.1)	−4.16	0.003**		No	−0.44 (−0.72; −0.23)
adjusted *p* = <0.001,		S4	−112 (−177; −47.4)	−5.13	0.0004***		No	−0.53 (−0.81; −0.31)
*ω*^2^ = 0.34, 95% CI [0.12, 0.52])	S2	S3	−45.3 (−110; 19.0)	−2.09	0.103		No	−0.21 (−0.44; −0.01)
		S4	−66.6 (−139; 5.85)	−2.72	0.045*		No	−0.30 (−0.56; −0.08)
	S3	S4	−21.3 (−80.2; 37.6)	−1.07	0.297		No	0.10 (−0.09; 0.30)
m505_L__L-Dec_Phase_Dec_avg_	S1	S2	−0.53 (−1.19; 0.12)	−2.42	0.086	1.57	No	−0.32 (−0.62; −0.6)
(*F*_(2.95, 53.15)_ = 10.37,		S3	−1.03 (−1.76; −0.30)	−4.17	0.003**		No	−0.68 (−1.08; −0.36)
adjusted *p* = 0.001,		S4	−1.25 (−2.06; −0.45)	−4.60	0.001**		No	−0.76 (−1.20; −0.42)
*ω*^2^ = 0.33, 95% CI [0.11, 0.50])	S2	S3	−0.49 (−1.19; 0.20)	−2.11	0.098		No	−0.33 (−0.67; −0.03)
		S4	−0.72 (−1.56; 0.13)	−2.52	0.086		No	−0.44 (−0.84; −0.09)
	S3	S4	−0.22 (−0.81; 0.36)	−1.14	0.27		No	−0.15 (−0.43; 0.10)
m505_L__L-Dec_HBF_avg_	S1	S2	−45.0 (−98.9; 8.93)	−2.47	0.095	129	No	−0.26 (−0.49; −0.05)
(*F*_(2.83, 50.86)_ = 9.32,		S3	−81.5 (−145; −17.9)	−3.80	0.007*		No	−0.49 (−0.82; −0.23)
adjusted *p* = 0.004,		S4	−100 (−168; −32.8)	−4.40	0.002**		No	−0.57 (−0.90; −0.30)
*ω*^2^ = 0.30, 95% CI [0.08, 0.47]),	S2	S3	−36.5 (−95.3; 22.2)	−1.84	0.164		No	−0.21 (−0.47; 0.01)
		S4	−55.4 (−126; 15.2)	−2.32	0.096		No	−0.3 (−0.60; −0.04)
	S3	S4	−18.9 (−65.6; 15.8)	−1.20	0.246		No	−0.11 (−0.30; 0.07)
m505_L__Dec_Phase_Dec_max_	S1	S2	−0.55 (−1.22; 0.13)	−2.41	0.108	1.53	No	−0.38 (−0.74; −0.07)
(*F*_(3, 54)_ = 8.39,		S3	−0.91 (−1.61; −0.20)	−3.82	0.006**		No	−0.66 (−1.09; −0.32)
adjusted *p* = 0.007,		S4	−1.12 (−1.91; −0.34)	−4.24	0.003**		No	−0.74 (−1.19; −0.38)
*ω*^2^ = 0.28, 95% CI [0.07, 0.45])	S2	S3	−0.36 (−1.02; 0.30)	−1.63	0.243		No	−0.28 (−0.65; 0.06)
		S4	−0.58 (−1.41; 0.26)	−2.05	0.167		No	−0.39 (−0.83; −0.01)
	S3	S4	−0.22 (−0.79; 0.36)	−1.11	0.282		No	−0.15 (−0.44; 0.11)
m505_L__Dec_Phase_Time	S1	S2	0.03 (0.004; 0.056)	3.44	0.015*	0.070	No	0.46 (0.21; 0.76)
(*F*_(3, 54)_ = 7.90,		S3	0.03 (−0.00; 0.06)	2.92	0.037*		No	0.49 (0.16; 0.87)
adjusted *p* = 0.011		S4	0.05 (0.02; 0.08)	5.37	0.0003***		No	0.79 (0.48; 1.19)
*ω*^2^ = 0.27, 95% CI [0.06, 0.44])	S2	S3	0.001 (−0.03; 0.03)	0.087	0.931		No	0.01 (−0.30; 0.33)
		S4	0.02 (−0.02; 0.05)	1.62	0.276		No	0.27 (−0.06; 0.63)
	S3	S4	0.02 (−0.01; 0.05)	1.78	0.276		No	0.26 (−0.03; 0.59)
m505_L__Dec_Phase_HBF_max_	S1	S2	−45.7 (−102; 10.4)	−2.41	0.107	126	No	−0.28 (−0.55; −0.05)
(*F*_(2.96, 53.21)_ = 7.66,		S3	−73.0 (−134; −12.5)	−3.58	0.011*		No	−0.46 (−0.78; −0.20)
adjusted *p* = 0.014,		S4	−90.3 (−157; −24.0)	−4.04	0.005**		No	−0.53 (−0.86; −0.27)
*ω*^2^ = 0.26, 95% CI [0.05, 0.43])	S2	S3	−27.3 (−81.8; 27.2)	−1.49	0.31		No	−0.17 (−0.41; 0.06)
		S4	−44.6 (−115; 25.5)	−1.89	0.227		No	−0.26 (−0.56; 0.01)
	S3	S4	−17.3 (−64.7; 30.1)	−1.08	0.31		No	−0.1 (−0.29; 0.08)
m505_L__Dec_Phase_Dec_avg_	S1	S2	−0.22 (−0.50; 0.07)	−2.27	0.143	0.69	No	−0.31 (−0.60; −0.06)
(*F*_(3, 54)_ = 6.02,		S3	−0.27 (−0.54; 0.004)	−2.92	0.045*		No	−0.37 (−0.64; −0.15)
adjusted *p* = 0.055,		S4	−0.39 (−0.66; −0.13)	−4.43	0.002**		No	−0.60 (−0.94; −0.33)
*ω*^2^ = 0.21, 95% CI [0.02, 0.38])	S2	S3	−0.05 (−0.33; 0.23)	−0.51	0.617		No	−0.06 (−0.30; 0.18)
		S4	−0.18 (−0.47; 0.12)	−1.77	0.283		No	−0.23 (−0.52; 0.03)
	S3	S4	−0.13 (−0.42; 0.16)	−1.31	0.415		No	−0.17 (−0.44; 0.08)
m505_R__L-Dec_Phase_Dec_avg_	S1	S2	−0.16 (−0.98; 0.67)	−0.57	1	1.61	No	−0.10 (−0.43; 0.22)
(*F*_(2.59, 46.65)_ = 7.82,		S3	−0.91 (−1.63; −0.18)	−3.70	0.008**		No	−0.54 (−0.88; −0.26)
adjusted *p* = 0.026,		S4	−0.89 (−1.81; 0.03)	−2.85	0.032*		No	−0.54 (−0.97; −0.18)
*ω*^2^ = 0.26, 95% CI [0.06, 0.43])	S2	S3	−0.75 (−1.32; −0.18)	−3.87	0.007**		No	−0.55 (−0.90; −0.27)
		S4	−0.73 (−1.33; −0.13)	−3.61	0.008**		No	−0.56 (−0.94; −0.25)
	S3	S4	0.02 (−0.54; 0.58)	0.097	1		No	0.01 (−0.26; 0.29)
m505_R__ReAcc_Phase_Acc_max_	S1	S2	0.10 (−0.85; 1.05)	0.32	1	1.88	No	0.007 (−0.38; 0.53)
(*F*_(3, 45)_ = 6.24,		S3	−0.84 (−1.64; −0.04)	−3.20	0.036*		No	−0.59 (−1.05; −0.22)
adjusted *p* = 0.056,		S4	−0.83 (−1.81; 0.15)	−2.57	0.064		No	−0.57 (−1.10; −0.13)
*ω*^2^ = 0.24, 95% CI [0.03, 0.43])	S2	S3	−0.94 (−1.85; −0.03)	−3.14	0.036*		No	−0.73 (−1.31; −0.26)
		S4	−0.93 (−1.85; −0.02)	−3.09	0.036*		No	−0.70 (−1.27; −0.24)
	S3	S4	0.009 (−0.70; 0.72)	0.039	1		No	0.01 (−0.36; 0.38)
105_L__L-Dec_Phase_Time (s)	S1	S2	0.01 (−0.02; 0.03)	0.75	0.461	0.044	No	0.14 (−0.23; 0.52)
(*F*_(2.22, 37.71)_ = 7.83,		S3	0.02 (−0.003; 0.05)	2.61	0.059		No	0.54 (0.13; 1.01)
adjusted *p* = 0.057,		S4	0.031 (0.01; 0.06)	3.93	0.007**		No	0.76 (0.37; 1.25)
*ω*^2^ = 0.27, 95% CI [0.06, 0.45])	S2	S3	0.02 (−0.004; 0.04)	2.32	0.066		No	0.39 (0.07; 0.77)
		S4	0.03 (0.002; 0.05)	3.25	0.024*		No	0.62 (0.24; 1.09)
	S3	S4	0.01 (−0.001; 0.02)	2.72	0.059		No	0.27 (0.07; 0.5)
105_R__Dec_Phase_Dec_max_	S1	S2	−0.13 (−0.81; 0.55)	−0.56	0.583	1.64	No	−0.009 (−0.41; 0.22)
(*F*_(3, 54)_ = 6.03,		S3	−0.58 (−1.34; 0.19)	−2.22	0.157		No	−0.39 (−0.79; −0.04)
adjusted *p* = 0.057,		S4	−0.94 (−1.71; −0.17)	−3.62	0.012*		No	−0.67 (−1.11; −0.31)
*ω*^2^ = 0.21, 95% CI [0.02, 0.38])	S2	S3	−0.45 (−1.21; 0.32)	−1.73	0.257		No	−0.33 (−0.75; 0.05)
		S4	−0.81 (−1.62; −0.01)	−2.98	0.040*		No	−0.63 (−1.13; −0.21)
	S3	S4	−0.36 (−0.96; 0.23)	−1.82	0.257		No	−0.29 (−0.63; 0.03)

Definition and description of all outcome variables are presented in Table 1. *F*, *F*-value (with corresponding degrees of freedom in parentheses) from repeated measures analysis of variance (RM ANOVA); adjusted *p*, Holm-Bonferroni method adjusted *p*-value from the RM ANOVA; *ω*^2^, omega squared effect size with 95% confidence interval; *t*, *t*-value from *post hoc t*-tests; mean difference (95% CI), mean difference with a 95% confidence interval; Holm-B adj. *p*-value, Holm-Bonferroni method adjusted *p*-value; mean diff, mean difference; MDC_90%_, minimal detectable change calculated with a 90% confidence interval; Hedges’g_av_ (95% CI), Hedges’g_av_ standardized effect size with a 95% confidence interval.

* *p* < .05.

** *p* < .01.

*** *p* < .001.

## 4. Discussion

This study aims to explore the test–retest reliability, MDC, and differences between the test sessions of phase-specific outcome measurements of CoD tests using an MRD. Overall, the values of test–retest relative and absolute reliability observed were mostly good to excellent and mostly acceptable to good, respectively. These findings were similar or better than those presented using other technology and slightly different tests (12, 15). Based on the MDC from the reliability statistics, no learning effects were observed between the sessions for the different outcome measurements.

The phase-specific analysis applied to CoD tests in the present study was based on the work of Harper and co-authors (15). In their study, they analyzed a maximum horizontal deceleration following a maximal 20-m sprint using a radar technology. Therefore, the players in the study by Harper and co-authors reached higher speeds prior to commencing deceleration than those observed in the present study. Furthermore, in the study by Harper et al. (2020), the deceleration phase was followed by a backpedal and not a CoD whereby the athlete rotated their body to maintain a forward-facing motion like in the present study. The CV values for the deceleration phase in the study by Harper et al. (2020) ranged from acceptable (5.2%, average deceleration) to poor (20.4%, time-to-maximum deceleration) for intra-day reliability and acceptable (6.2%, maximum horizontal braking power) to poor (21.6%, average late horizontal braking power) for inter-day reliability (15). In the present study, almost all deceleration variables across all CoD tests had good to acceptable absolute reliability, which demonstrated the potential of MRD during various 180-degree CoD tests for assessing deceleration ability. Since good to acceptable levels of reliability were also demonstrated with both left and right foot turns across most deceleration-phase variables, inter-limb asymmetries based on the turning foot may also be explored.

It is interesting to note that a minimum of 31 of 57 outcome variables had a good absolute reliability for the comparison of sessions 3–4, with longer 155 CoD tests (41 and 42 variables) having more than the shorter m505 tests (32 and 31 variables). Specifically, phase 1a and initial acceleration (time, distance, and velocity) had consistently good absolute reliability across tests (Tables 5–10), while the overall deceleration and early deceleration outcome measurements (time, distance, velocity, acceleration, force, power, and momentum) had mostly good absolute reliability in the longer tests (155), with mostly acceptable values in the shorter tests (m505 and 105). On the contrary, no such differences were observed for the same outcome variables during the late deceleration phase with mostly acceptable absolute reliability across tests. Furthermore, both the overall and late re-acceleration phases had mostly good absolute reliability for time, distance, velocity, acceleration, force, power, and impulse except for maximum acceleration and force (acceptable absolute reliability). A similar pattern for early re-acceleration as for the late deceleration was observed with mostly acceptable absolute reliability for the different outcome measurements for all tests. Overall, it appeared that longer tests improved reliability, with late deceleration and re-acceleration not being impacted. This observation is possibly attributed to both greater distance and time available for possible pacing strategies in preparation for both the overall and early deceleration phases. However, this must be substantiated by future research. Nevertheless, apart from distance, acceptable reliability was observed during the late deceleration and re-acceleration phases. This possibly indicated that the participants chose slightly different late deceleration strategies between sessions, which in turn could influence early re-acceleration. See Tables 5–10 for details.

Phase-specific information has also been previously explored using two synchronized laser guns (12). In the study of Hader et al. (2015), the phase-specific information was explored using 45- and 90-degree CoD angles. This was different compared with the present study that used CoD tests involving 180-degree turns. Specifically, Hader and co-authors explored the reliability of maximum and distance-to-maximum acceleration, deceleration, and speed along with minimum speed and speed between 8 m and 12 m that represented the distance 2 m prior and 2 m after the CoD. The CV values for speed around the 8–12 m distance were ∼5%, and the CV values for phase-specific information ranged from 6.6% to 8.5% for peak acceleration, while peak and distance-to-peak deceleration ranged from 117% to 12.6%. Thus, the results observed for both speed and acceleration in their study were comparable with the present results, while a better absolute reliability was observed for deceleration-specific outcome measurements (good to acceptable vs. poor to very poor) in the present study (12). However, it is important to note that Hader and co-workers explored intra-session reliability, while inter-session reliability was explored in the current study making values not directly comparable. Nevertheless, having reliable assessment of deceleration specific outcome measurements is important considering the importance of deceleration in team and individual multi-directional speed sports (35).

Similar to the study of Harper et al. (2020), the protocols adopted in the present study permitted a more detailed analysis of the deceleration phase than that previously investigated during the CoD maneuvers. This could have a notable importance, since the deceleration phase has been associated with a superior CoD performance and major injuries such as anterior cruciate ligament ruptures in many multi-directional sports (34, 37). In the study by Harper and co-authors (15), the average *V*_max_ defining the start of the deceleration phase was between 7.19 m·s^−1^ and 7.36 m·s^−1^, which was most comparable with the values observed during the 155 test (6.78 m·s^−1^), but not to those observed during neither the m505 (4.46 m·s^−1^) nor the 105 test (5.94 m·s^−1^). Thus, the comparisons with the deceleration outcome measurements were most appropriate for the 155 tests. Specifically, the overall and early deceleration outcome measurements (deceleration, force, and power) were slightly higher in magnitude in the present study compared with those presented by Harper and co-authors (15). However, large differences in deceleration are observed during the late deceleration phase. Specifically, Harper and co-authors reported a Dec_avg_ during the late deceleration of 5.53 m·s^−2^−5.55 m·s^−2^, while our present findings were 9.80 m·s^−1^ and 10.26 m·s^−1^ for the left and right foot turn, respectively, during session 4. This is an interesting finding which could be due to the differences in both distance and time for the deceleration phase. Harper and co-authors reported a longer distance (6.53 m−6.71 m), while 5.70 m and 5.66 m were observed in the present study. In addition, the participants used a shorter time in the deceleration phase in the present study (1.29 s and 1.32 s) compared with 1.47 s–1.49 s reported by Harper and co-authors (15). Overall, it appeared that the deceleration from a 20-m sprint, compared with performing a CoD test, provided a relatively lower demand on the late deceleration phase. One plausible reason for the late deceleration differences was possibly that a CoD task had a different intent in that one was to complete a course in the shortest amount of time with a clearly defined turning point, whereas in a maximum deceleration test, the focus was to maximally decelerate from a 20-m sprint, thereby removing the skill required to change direction. Furthermore, an assisted deceleration prior to a CoD, as provided by an external load with a MRD, placed increased demands on the late deceleration. In addition, it is also possible that decelerating to a stop while facing the same direction has a decreased deceleration demand compared with the use of a plant step in a 180-degree CoD whereby a more horizontally orientated body posture may facilitate braking and thus deceleration prior to the CoD. This may be particularly the case when using the assisted load. Nevertheless, these findings have crucial implications on how one possibly uses different deceleration tasks in both training and rehabilitation.

Performance times of the m505 test in the present study are comparable with those that are previously reported (8). Specifically, slightly higher times for the m505 test (3.01 s to 3.04 s) were observed compared with the unloaded m505 in professional rugby players (2.73 s) (38) and netball players (2.82 s–2.88 s) (39). Furthermore, slightly lower times were observed compared with the same loaded conditions using the MRD (3.26 s) (14). However, it is important to consider that the measurements in the present study were obtained using the MRD with a 0.2 m·s^−1^ start trigger, which is possibly more sensitive to capturing the start of the test compared with the data obtained from the other studies using timing gates. Specifically, the same MRD device had been found to measure greater 0 m–5 m sprint split times compared with photocells with a bias of 0.33–0.35 s (17). Furthermore, it is important to consider that the external load provided by the MRD will impact the overall CoD test performance time through providing “assistance” and “resistance” during different phases of the CoD. For example, during the initial acceleration-to-deceleration phase (1a) and due to the positioning of the MRD, the athlete is “assisted” as they accelerate and then decelerate prior to CoD. In contrast, during the re-acceleration phase (1b), the athlete is faced with a resisted load that would essentially reduce whole-body acceleration. This will make both deceleration and re-acceleration more demanding. Furthermore, the inclusion of both males and females and different sports (soccer, handball, and floorball) improves the reliability to encompass different ball sports and gender.

### 4.1. Limitations and implications for future research

Both limitations and possibilities for future research have been highlighted in the present study. One limitation is that a total of 57 variables were assessed for each test in three different tests. The variables in the different phases of a CoD task that are essential to the performance need to be analyzed, as documenting or monitoring 57 variables is impractical. However, the reliability statistics of all 57 variables allows for future exploration of which variables are important and how different phases determine the CoD performance. Furthermore, it is possible that phase-specific variables possibly dictate what phases to target in individualized training, which in turn could lead to phase-specific interventions (i.e., deceleration). Then, phase-specific outcome variables can be used to determine if there was a “real” change in performance and which variables responded to different types of training. In addition, three different tests were further assessed that increased the number of variables included. The reason for exploring these three different tests was to manipulate the entry velocity and thereby the momentum to progressively increase the deceleration-phase demands. Considering that the limited deceleration assessments are currently available, it is possible that using more than one test to assess the deceleration capacity is possibly important in future CoD assessment. Another limitation is that only a 180-degree CoD was used for the analysis of the CoD performance. However, the 180-degree CoD tasks require the players to perform the maximum deceleration prior to re-acceleration permitting the evaluation of the initial acceleration, deceleration, and re-acceleration capabilities of the players. Nonetheless, a future research should consider the evaluation of phase-specific CoD information during the performance of other CoD tasks that require different angles of CoD. Another limitation is that the CoD tasks were only performed with an assisted start, which increases the demands on the deceleration and re-acceleration phases due to the direction of the external load provided by the MRD. Future research should explore different external loads for both and assisted and resisted start relative to the MRD to allow for a better generalizability of the present findings as well as to further assess how this may change the demands in different subphases of CoD (i.e., early and late deceleration). Moreover, testing was performed in an indoor sports hall environment. Obtaining data from an outdoor setting and from an artificial turf would have improved the generalizability since applications to the CoD testing and training using an MRD apply to those environments. However, many team and individual ball sports are performed in indoor environments, and we wanted to explore the reliability in this environment.

## 5. Conclusion

The phase-specific outcome measurements of different CoD tests obtained and calculated from the data captured by using the MRD are mainly reliable. The analysis yielded mostly good to excellent ICC values and mostly acceptable to good CV values for the test–retest reliability across multiple sessions. The test–retest reliability of different CoD tests provides coaches and researchers alike with new opportunities to further assess and understand this important athletic quality.

## 6. Practical applications

Our findings may influence not only the lab but also the field-based testing and training of CoD. In combination with the previously established validity of velocity measurements of the MRD (14), the present findings provide coaches with important information that was previously only available in a laboratory setting. Based on the valid and reliable information that can be obtained by the MRD, we now have an opportunity to obtain more in-depth insights into an athletes CoD performance that will enable more advanced athlete specific CoD training prescriptions.

## Data Availability

The raw data supporting the conclusions of this article will be made available by the authors, without undue reservation.
